# Amazonian Bioactives in Biopolymer-Based Systems for Biomedical Applications: Current Advances and Future Perspectives

**DOI:** 10.3390/ma19163559

**Published:** 2026-08-21

**Authors:** Márcia Raquel Felix da Costa, Yuliana Padron-Antonio, Sara Caroline Pacheco de Oliveira, Lucas de Souza Falcão, Patrícia Melchionna Albuquerque, Mariana Agostini de Moraes

**Affiliations:** 1 Department of Materials and Bioprocess Engineering, School of Chemical Engineering, Universidade Estadual de Campinas, Campinas 13083-852, Brazil; marci.raquel@hotmail.com (M.R.F.d.C.); s289287@dac.unicamp.br (S.C.P.d.O.); 2 Grupo de Pesquisa Química Aplicada à Tecnologia, Escola Superior de Tecnologia, Universidade do Estado do Amazonas, Manaus 69050-020, Brazil; yuliana.padron@hotmail.com (Y.P.-A.); lucas.sfalcao@hotmail.com (L.d.S.F.); palbuquerque77@gmail.com (P.M.A.)

**Keywords:** Amazon biodiversity, bioactives extraction, biopolymers, biomaterials

## Abstract

The Amazon region harbors one of the richest biodiversities on the planet, representing a vast reservoir of bioactive compounds with high therapeutic potential. Although several Amazonian bioactives have already been incorporated into biopolymeric matrices, the effects of their incorporation on the mechanical, barrier, and biological properties of these materials remain insufficiently systematized. This review addresses this knowledge gap by critically analyzing reported Amazonian bioactive–biopolymer systems, with emphasis on how bioactive incorporation influences matrix properties, release behavior, and biological performance, particularly in biomedical applications. Emphasis is placed on the role of polymer–bioactive interactions in modulating physicochemical, mechanical, and transport properties, as well as in controlling release kinetics and biological performance. Different incorporation strategies, such as encapsulation and matrix embedding, are critically discussed in terms of their impact on stability and functionality. Overall, the reported studies demonstrate that Amazonian bioactives can substantially modulate the physicochemical, mechanical, barrier, and biological performance of biopolymeric systems, depending on the bioactive, polymeric matrix, and incorporation strategy. However, challenges related to variability in raw materials, lack of standardization, and limited reproducibility remain key barriers to translation and large-scale application.

## 1. Introduction

The Amazon, which spans nine South American countries and harbors approximately 10% of the world’s known plant species, stands out as one of the largest reservoirs of bioactive compounds with pharmacological, cosmetic, and biomedical potential [1,2]. Fruits, leaves, barks, and microorganisms associated with Amazonian flora present a wide diversity of bioactive compounds [3,4]. Among polyphenols, noteworthy examples include stilbenes, flavonoids such as anthocyanins, methoxyflavones, flavonols, and flavones [5,6]. Phenolic compounds and derivatives include ellagic acid and spilanthol [7,8]. Within carotenoids and liposoluble vitamins, tocopherols are prominent [9,10]. For unsaturated fatty acids, oleic acid and linoleic acid stand out [11]. Finally, among alkaloids and terpenoids, andirobin, theobromine, and theophylline can be highlighted [12,13]. These bioactives have demonstrated antimicrobial, antioxidant, cytotoxic, wound healing, and immunomodulatory properties, making them promising targets for the development of functionalized biomaterials [14].

Despite its enormous potential, much of the Amazonian biodiversity remains unexplored, with many plant species and microorganisms yet to be cataloged, particularly regarding the secondary metabolites of native species and their endophytic microorganisms [15]. Nevertheless, ensuring the stability and bioavailability of these compounds remains a challenge, as they are highly susceptible to degradation by factors such as light, oxygen, and temperature, as well as seasonal variations [2].

In this scenario, scientific interest has increasingly focused on strategies that enable the incorporation of Amazonian bioactivity for biomedical, pharmaceutical, and cosmetic applications, aiming not only to preserve functional properties but also to enhance efficacy through controlled release approaches [16,17]. Within this context, biopolymers—a class of materials derived from renewable sources that combine biocompatibility, biodegradability, and tunable physicochemical properties—have emerged as matrices for the development of systems capable of protecting the bioactive from degradation and modulating release profiles [18,19]. Biopolymers such as chitosan, alginate, starch, gelatin, xanthan gum, pectin, and silk fibroin, among others, have been widely explored for their ability to form hydrogels [20], films [17,21,22], scaffolds [23,24,25], particles [26,27,28], and other structures that interact safely and functionally with biological systems [29,30,31].

The association of these natural compounds with biopolymeric matrices allows for the modulation of release profiles [32,33,34], protection against degradation, and the preservation of the active compound’s stability [18,19,24,35,36], while also improving structural properties such as mechanical strength [23,37], and thermal stability [20,38]. In parallel, the bioactive–biopolymer association also enhances the biological effects of the compounds, promoting antimicrobial [10], antioxidant [21,38,39], anti-inflammatory [40,41], and cell viability maintenance activities [19,21,24,42,43].

An important advantage of these technological platforms lies in their remarkable versatility. Depending on the biopolymer–bioactive combination, specific properties are achieved that can be modulated according to the intended application, including distinct release profiles, responsiveness to environmental stimuli (such as pH variations, temperature, and the presence of enzymes), and the ability to interact with biological tissues. Hydrogels, for instance, mimic the hydrated environment of the extracellular matrix, promoting cell adhesion and proliferation, and are widely used in tissue engineering and wound healing applications [21]. Films offer benefits such as strong adhesion to mucosal surfaces, controlled local release of active compounds, and protection against environmental degradation, making them suitable for topical and transmucosal applications [44,45]. Nanostructures, particularly nanoparticles and nanoemulsions, significantly enhance the stability and bioavailability of bioactive compounds due to their reduced particle size and increased surface area, which facilitates more efficient cellular penetration and targeted delivery [42,46,47]. This functional modularity enables the development of smart and personalized biomaterials capable of dynamically responding to the biological microenvironment, thereby expanding their potential in advanced therapeutic applications in the medical, pharmaceutical, and cosmetic fields.

Although reviews addressing the potential of Amazonian compounds already exist, most are limited to describing a single fruit, plant, or microorganism, or present general compilations of bioactive properties without exploring their technological applications in detail. It is important to note that extensive reviews have covered the field of essential oils and other well-characterized bioactive compounds in macromolecular systems. However, for Amazonian plant extracts specifically, a significant gap can be observed in the literature regarding the integrated organization of information on their bioactive properties and potential applications across different technological platforms, with particular emphasis on biopolymeric systems designed for therapeutic and biomedical uses.

This review aims to fill that gap by critically compiling and analyzing the main scientific evidence on bioactive compounds derived from Amazonian biodiversity and their incorporation into functional biomaterials. This work integrates updated information on chemical composition, biological properties, and technological applications, providing a solid basis to drive the development of innovative biomaterials with high therapeutic and biotechnological potential.

To support this review, a systematic literature search was conducted across the Web of Science, ScienceDirect, and PubMed databases, covering publications from 2010 to 2025. The search terms included bioactive, compound, Amazon OR Amazonian, biomaterials, biopolymers, and applications. Additional searches combining each Amazonian species with with biopolymer retrieved studies on their integration into technological platforms.

**Figure 1:**
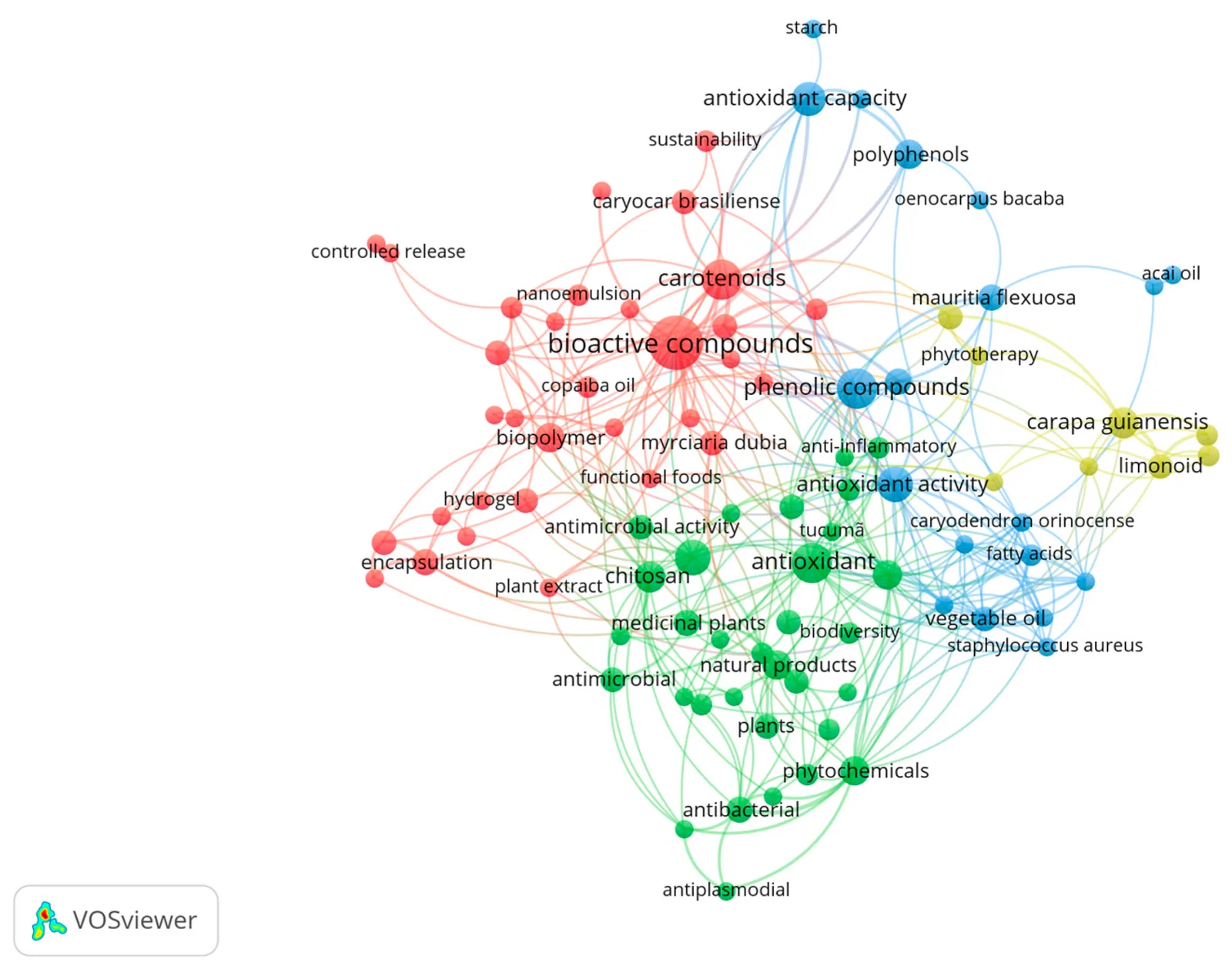
A co-occurrence network of author keywords derived from the bibliometric analysis. Four main thematic clusters can be identified. The first cluster (red) encompasses terms related to bioactive compounds (e.g., antioxidant, antimicrobial, phenolic compounds, carotenoids) and their biological activities. The second cluster (green) is associated with biopolymeric materials, particularly chitosan and its applications in encapsulation and wound healing. The third cluster (blue) includes topics related to medicinal plants and natural products from the Amazon. The fourth cluster (yellow) groups terms related to cytotoxicity and pharmacological applications. The size of each node reflects the frequency of the keyword, while the links indicate co-occurrence patterns, suggesting that research on Amazonian bioactive compounds is strongly linked to their incorporation into biopolymeric matrices for biomedical and pharmaceutical applications.

To complement this mapping and identify key thematic clusters, a bibliometric co-occurrence analysis was performed on the author keywords of the retrieved documents using VOSviewer (version 1.6.20). The analysis was based on 272 documents, and a threshold of minimum occurrences was applied to select the most representative terms. The resulting network visualization (Figure 1) grouped keywords into thematic clusters, revealing the main research fronts and their interconnections. This approach helped to identify the predominant topics (e.g., antioxidant activity, antimicrobial activity, chitosan, and bioactive compounds) and to detect emerging trends and gaps in the literature, which will guide the scope and structure of this review.

## 2. Amazonian Biodiversity and Its Bioactive Compounds

**Figure 2:**
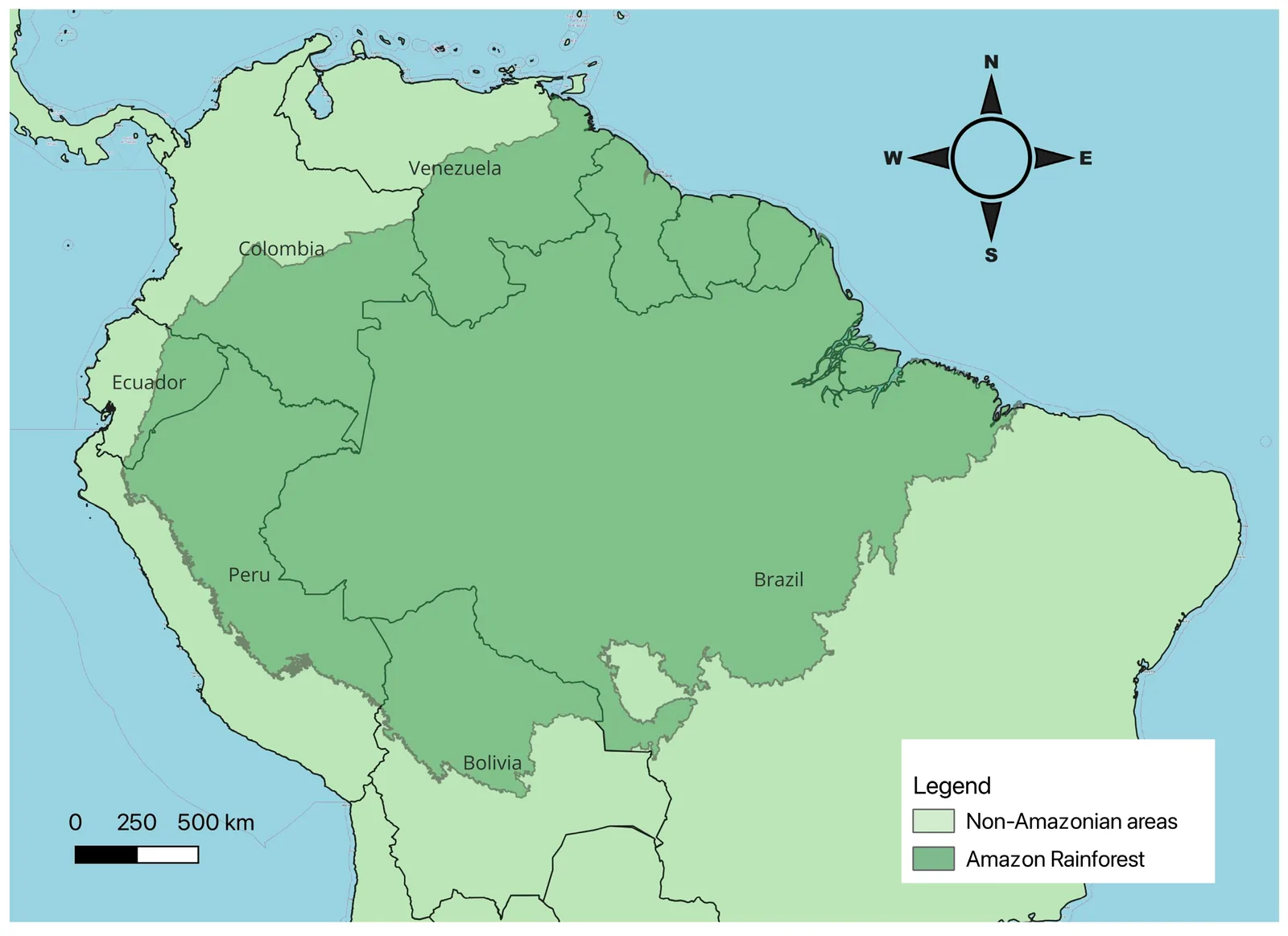
Geographic distribution of Amazon region across South America. Geospatial data were obtained from PRODES/INPE, MapBiomas Amazonia, and RAISG. Data processing using MAAP platform. Map generated with QGIS v3.32. See Acknowledgments for details.

With an area close to 6 million square kilometers, the Amazon region represents approximately 4.9% of the planet’s continental surface. This vast area extends across several South American countries, including Brazil, Bolivia, Colombia, Ecuador, Guyana, Peru, Suriname, Venezuela, and French Guiana [48] (Figure 2).

The Amazon stands out for its remarkable plant diversity. A study published in 2017 identified a total of 14,003 plant species in the region, 6727 of which are tree species [15]. This extraordinary biodiversity has long served as an invaluable source of traditional knowledge, especially among Indigenous communities, whose ancestral medicinal practices have attracted scientific interest over the centuries [13].

The Amazon region hosts immense biological wealth, including numerous plants of high pharmacological, nutritional, and cosmetic interest [49,50,51]. These plants produce a wide range of secondary metabolites with diverse biological activities, including antimicrobial, antioxidant, antitumor, antiviral, and anti-inflammatory effects [43,52,53,54].

Recent advances in extraction techniques, analytical methods, and phytochemical analysis have enabled more efficient characterization and identification of bioactive compounds present in medicinal plants [55]. These developments have contributed to a deeper understanding of the chemical composition and biological activities of such plants, opening new possibilities for the development of pharmacological, nutritional, and cosmetic formulations [56,57,58].

The production of secondary metabolites in plants depends on energy-intensive physiological processes, as well as on the availability of essential elements that participate in enzyme-regulated metabolic pathways within the cellular metabolism [59]. Secondary metabolites produced by plants are often associated with biological activities of pharmacological potential, and their ability to synthesize these compounds allows them to be classified as medicinal plants [60].

Among the chemical compounds found in plants, three main classes are recognized, terpenoids, phenolic compounds, and alkaloids, along with other nitrogen-containing plant-derived metabolites [61]. Additionally, there are many others classes of bioactive compounds, such as fatty acids, essential oils, pigments, and vitamins, which also have significant biotechnological value [62,63,64].

This is why Amazonian bioactives present significant biotechnological potential, which has generated growing interest in their study. Consequently, the following sections will present some of the main plant sources from the Amazon region, organized according to their botanical classification to provide a systematic and ecologically relevant overview. The bioactive compounds identified in these species will be discussed accordingly. The data are also consolidated in Table S1, including the main identified compounds, biological activities and biopolymeric matrices studied for incorporation.

### 2.1. Arecaceae

**Figure 3:**
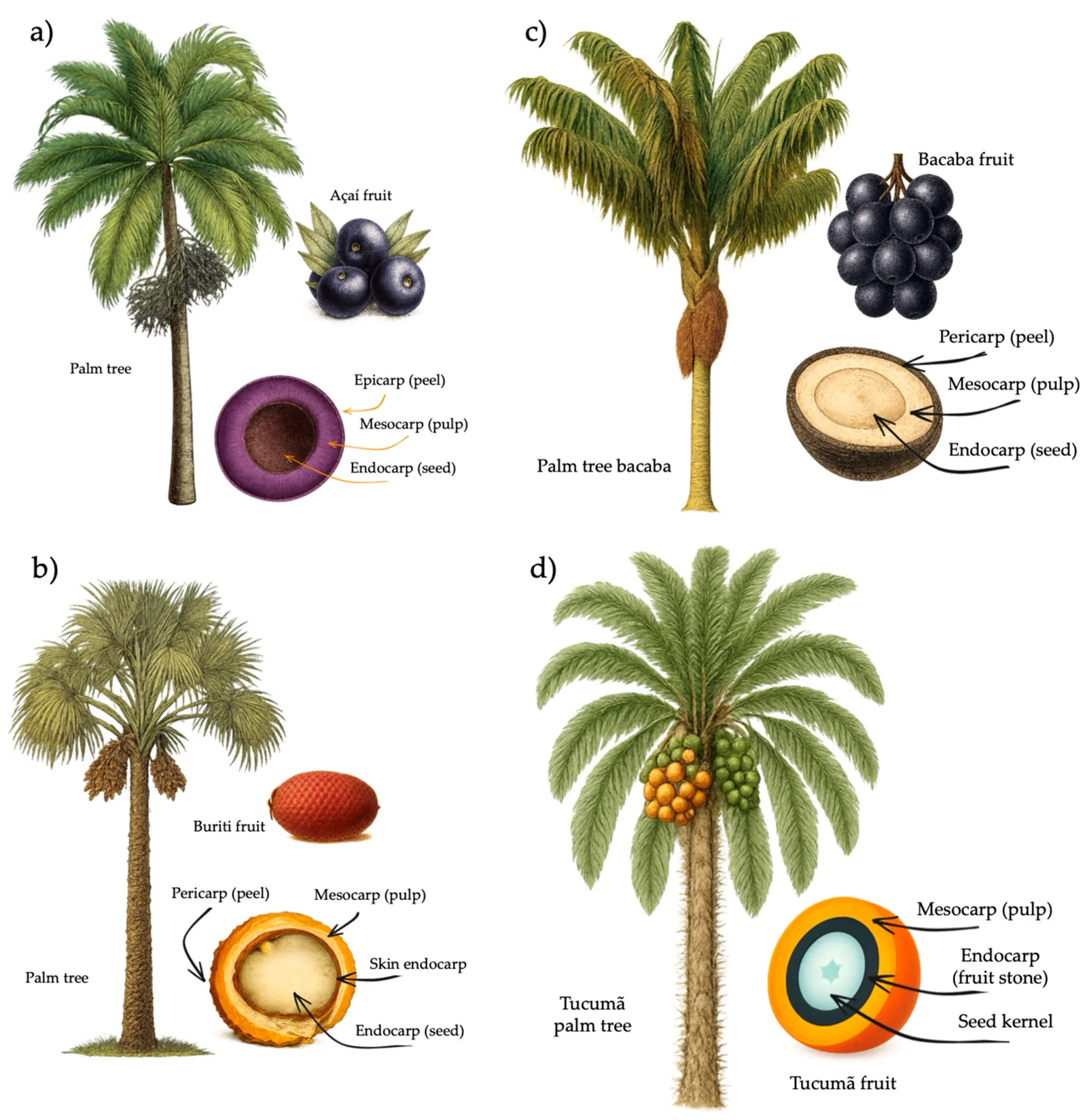
Representative Arecaceae species from the Amazon: (**a**) açaí (*Euterpe oleracea*), (**b**) buriti (*Mauritia flexuosa*), (**c**) bacaba (*Oenocarpus bacaba*), and (**d**) tucumã (*Astrocaryum aculeatum* and *A. vulgare*). Illustrations generated using the Illustrae platform. See the Acknowledgments for details.

The Arecaceae species analyzed in this study are shown in Figure 3a–d, including *Euterpe oleracea* (açaí), *Mauritia flexuosa* (buriti), *Oenocarpus bacaba* (bacaba), and *Astrocaryum* spp. (tucumã).

#### 2.1.1. Açaí (*Euterpe oleracea*)

*Euterpe oleracea* is a palm native to the tropical regions of South America, particularly the Amazon region of Brazil, Colombia, Ecuador, Peru, Suriname, and Venezuela. Its fruit, commonly known as açaí (Figure 3a) [65], is a small drupe with a thin pulp surrounding a large seed. The pulp is widely consumed and has recently been promoted as a “superfruit” due to its richness in antioxidants and bioactive compounds with potential health benefits [50].

Açaí has been extensively studied because of its richness in bioactive compounds, such as flavonoids, phenolic acids, anthocyanins, fatty acids, and phytosterols. These compounds exhibit a variety of biological activities, including antioxidant, anti-inflammatory, and cardioprotective effects, which contribute to cardiovascular health and may help prevent neurodegenerative diseases [66,67,68].

The oil extracted from the seed of açaí fruit was applied as a bioactive component in therapeutic delivery systems across different studies, offering a potential treatment against cervical cancer [69] and melanoma [70]. Finally, açaí extract in emulsions has also been investigated. Owing to its high polyphenol content and antioxidant activity, its use has been reported in anti-aging cosmetic formulations, where the extract contributed to physicochemical and microbiological stability, as well as protection against UV radiation [71].

#### 2.1.2. Buriti (*Mauritia flexuosa*)

*Mauritia flexuosa*, commonly known as buriti, aguaje, moriche, or canangucho depending on the region, is a palm species that thrives in the tropical regions of South America, particularly in the Amazon areas of Brazil, Peru, Colombia, and Venezuela [72], and its chemical composition may vary depending on the climatic conditions specific to each cultivation zone [73]. The fruit, as illustrated in Figure 3b, is a drupe covered with overlapping scales whose edible mesocarp is notable for its high carotenoid content, especially β-carotene, and surrounds a large, hard, ellipsoid seed [65,74].

Among the reported biological activities of *M. flexuosa* (buriti) fruit are acaricidal properties attributed to the presence of fatty acids [75], as well as antiplatelet and antithrombotic activity associated with tocopherols. In addition, antioxidant, antimicrobial, prebiotic, antidiabetic, and anticancer properties have been described, mainly due to the presence of polyphenols and tocopherols [76,77]. Furthermore, Koolen et al. [78] analyzed *M. flexuosa* roots and reported the presence of terpenes with cytotoxic and antimicrobial activity. In a subsequent study, Bataglion et al. [79] described additional biological properties, including antidiabetic, anticancer, and antioxidant activities. Abreu et al. [80] further highlighted the bioactive potential of *M. flexuosa* fruits by characterizing their phenolic profile, antioxidant capacity, and effectiveness against lipid peroxidation of cell membranes. Other studies have confirmed the presence of methoxyflavones and anthocyanins [5,6].

In terms of its biotechnological applications, the fruit has gained attention in the food industry as a natural source of pigments, being incorporated into pectin-based matrices [81]. Buriti oil has also been encapsulated with biopolymers such as gelatin and alginate through emulsification and freeze-drying, which improved its stability, water dispersibility, and antimicrobial potency, thus expanding its potential use in food or pharmaceutical products [82]. Gum arabic has likewise been used as an encapsulating agent for this oil, protecting the encapsulated compounds during storage, so far only in food matrices [83].

#### 2.1.3. Bacaba (*Oenocarpus bacaba*)

*Oenocarpus bacaba,* as shown in Figure 3c, is a palm native to the tropical regions of South America, especially the Brazilian Amazon and the Cerrado (savanna) [15]. Its fruit is known as bacaba, although in some regions it is also referred to by other local names, such as bacaba-de-leque [65]. It belongs to the Arecaceae family. The fruit is a rounded drupe, approximately 1 to 1.5 cm in diameter, which develops a dark purple or blackish color upon ripening. The pulp has a creamy consistency and mild flavor, is oily, and surrounds a single seed. The fruit presents a pulp whose color ranges from white to brown, depending on the stage of ripeness. Harvesting is carried out through extractivist systems, mainly by Indigenous and riverside communities. It is often marketed in local markets, and its consumption is similar to that of açaí, either in its pure form, as juice or wine, or as an ingredient in desserts such as ice creams and jellies [84]. Additionally, its colorless oil has a mild flavor and is used in food preparation [85].

Bacaba is appreciated both for its flavor and nutritional value, as it is rich in fiber and lipids [9,86]. It has attracted scientific interest due to its content of bioactive compounds such as anthocyanins, phenolic acids, fatty acids, carotenoids, and phytosterols. These compounds are associated with antioxidant, anti-inflammatory, and neuroprotective activities, positioning this fruit as a functional food with potential for preventing chronic diseases, including cardiovascular and neurodegenerative disorders [7,39,87,88,89,90]. Its use in biopolymer matrices has been reported, especially in studies that aim to improve bioactive stability and bioavailability. Its incorporation into biopolymers has so far been restricted to food-oriented systems, such as films with the phenolic compounds of *Oenocarpus* sp. combined with chitosan and acetylated cashew gum for active packaging [39,91]. The composition and the biological activities described above, however, suggest that this species could also be exploited in biopolymer-based pharmaceutical systems.

#### 2.1.4. Tucumã (*Astrocaryum aculeatum* and *A. vulgare*)

The palm trees of the genus *Astrocaryum*, belonging to the family Arecaceae, comprise around forty species distributed across South and Central America. Among them, two Amazonian species stand out for their economic relevance and medicinal potential: *Astrocaryum aculeatum*, commonly known as tucumã-do-Amazonas, and *Astrocaryum vulgare*, referred to as tucumã-do-Pará [89]. The latter is a palm native to tropical regions of South America, especially the Amazon biome in Brazil, where it is widely distributed in the states of Pará, Amazonas, and Maranhão. It is also found, though to a lesser extent, in Suriname, French Guiana, and Colombia [92]. The fruits of tucumã, as shown in Figure 3d, exhibit a shape that ranges from ellipsoidal to globose with a pulp that is firm, fibrous, and oily, with a characteristic sweet flavor. It is particularly rich in carotenoids, especially beta-carotene, which gives it a high nutritional value. In the Amazon region, tucumã is an important component of the traditional diet and is widely consumed both in its natural form and in products such as juices, ice creams, artisanal wines, and traditional dishes [93].

Tucumã has garnered growing scientific interest due to its lipid profile and the presence of bioactive compounds with antioxidant and anti-inflammatory potential, mainly associated with its carotenoid and phenolic compound content [94]. This fruit stands out as a rich source of macronutrients, such as fatty acids and dietary fiber, as well as bioactive compounds in lower concentrations, including flavonoids, taninns, carotenoids and phenolic compounds [93,95,96,97]. Among its biological potentials, the antioxidant, anti-inflammatory, antimicrobial, genotoxic/antigenotoxic, and antidiabetic activities are noteworthy [98,99].

### 2.2. Asteraceae

#### Jambu (*Acmella oleracea*)

**Figure 4:**
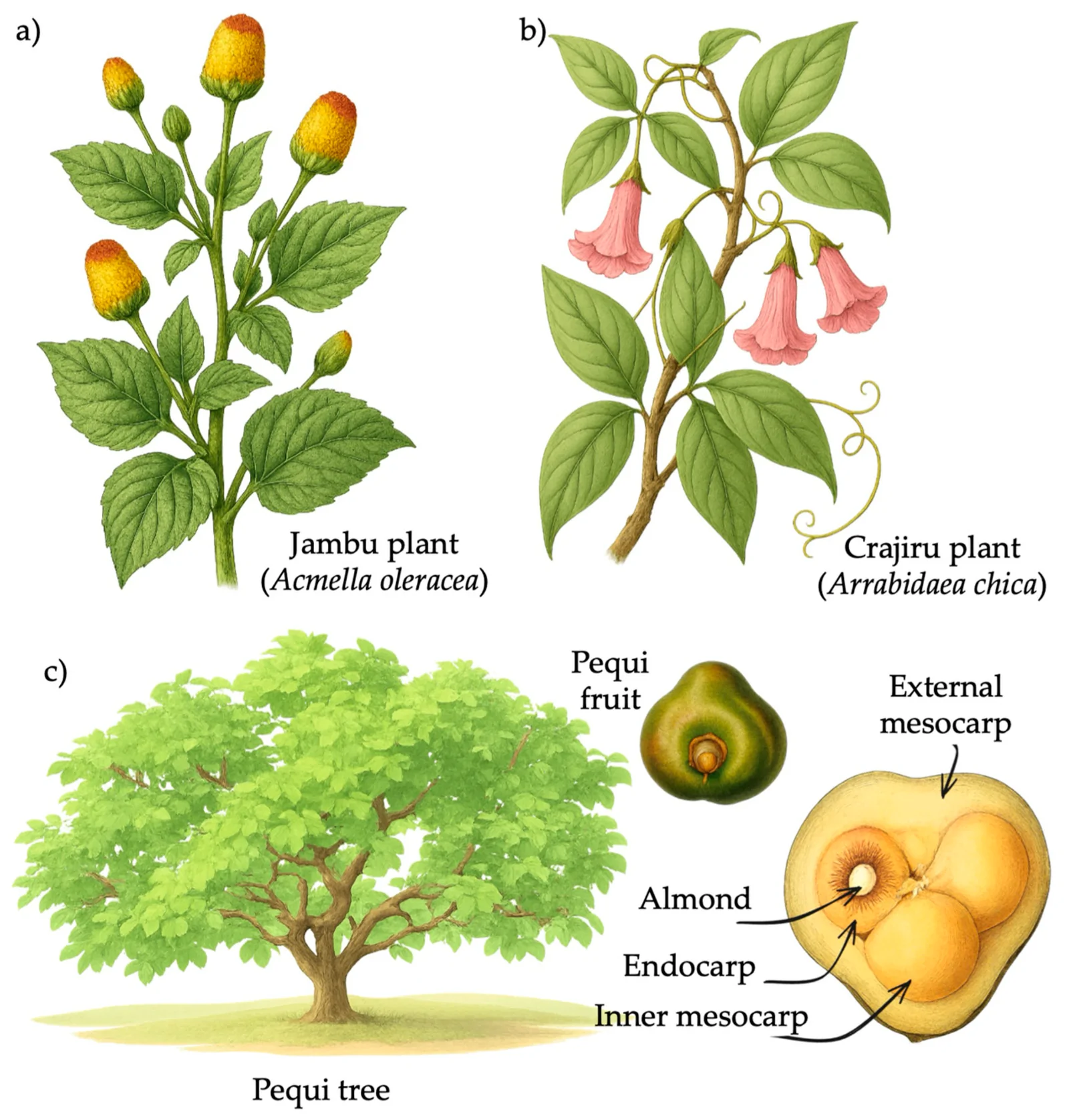
Representative Amazonian species from the Asteracea, Bignoniaceae, and Caryocaraceae families: (**a**) *Acmella oleracea* (jambu), (**b**) *Arrabidaea chica* (crajiru) and (**c**) *Caryocar brasiliense* (pequi). Illustrations generated using the Illustrae platform. See the Acknowledgments for details.

*Acmella oleracea*, as shown in Figure 4a, commonly known as jambu, is a plant that has been widely used in culinary applications and studied for its medicinal properties. Native to the Amazon region and belonging to the Asteraceae family, jambu plays a significant role in traditional medicine, being used to relieve toothaches and treat bacterial and fungal infections. Additionally, extracts from jambu leaves have been shown to increase fibroblast migration, indicating the plant’s potential in tissue repair treatments [100].

Jerônimo et al. [4] evaluated the chemical composition, antioxidant potential, and cytotoxic effects of essential oils from *A. oleracea*. These oils exhibited antiproliferative activity against three human cancer cell lines. It is important to note that E-caryophyllene is the main compound found in *A. oleracea* essential oil, a substance known for its cytotoxic activity against epithelial cells with cancerous mutations [4]. Another significant molecule present in *A. oleracea* is spilanthol, a recognized alkamide with biological activity, including anti-inflammatory, bacteriostatic, analgesic, and antioxidant effects [8]. Additionally, spilanthol has a muscle-relaxing effect with excellent dermal penetration, known as “herbal botox” [101].

### 2.3. Bignoniaceae

#### Crajiru (*Arrabidaea chica*)

The plant *Arrabidaea chica*, as shown in Figure 4b, commonly known as crajiru, belongs to the family Bignoniaceae, being an ivy that has been used in the treatment of wounds and skin diseases, infections in the urinary tract. Studies suggest that it has activity against the trypomastigote form of *Trypanosoma cruzi*. The interest in the biological activities is justified by its chemical composition, being rich in anthocyanins and flavonoids, demonstrating the potential that *A. chica* has for antioxidant and antimicrobial activity [102].

Jorge et al. [103] evaluated the potential of *A. chica* for wound healing activity, using in vitro tests for fibroblast growth stimulation and also in vivo with Wistar rats. The cells treated with *A. chica* crude extract reported a three-fold increase in collagen synthesis. Additionally, *A. chica* extract showed in vivo activity analogous to the allantoin, used as positive control. Amaral et al. [102] registered antioxidant activity for the leaves of *A. chica*, especially the fraction that contained apigenin and luteolin, the last one was isolated and reached positive results for diuretic activity. Another demonstration of the promising bioactivities of *A. chica* is the work of Mafioleti et al. [104], which evaluated the antimicrobial activity present in the leaves of *A. chica*, and reported positive results against *Enterococcus faecalis* and *Helicobacter pylori*, probably due to the presence of phenolic compounds detected in the extract, where it was detected tannins, anthocyanins and flavonoids. In the phenolic compound found in *A. chica*, kaempferol was highlighted by the author as a molecule of potent antimicrobial effect.

### 2.4. Caryocaraceae

#### Pequi (*Caryocar brasiliense*)

Pequi, illustrated in Figure 4c, is produced by *Caryocar brasiliense*, a perennial tropical tree of the Caryocaraceae family found across several Brazilian biomes, including the Amazon, Caatinga, Cerrado, and Atlantic Forest [105], although it is primarily considered to have originated from the Brazilian Cerrado [106]. Its fruit is a drupe whose creamy, aromatic inner mesocarp, of intense yellow-orange color, envelops one to four seeds [107]. The oil extracted from this pulp is of significant importance for the pharmaceutical and food industries [108].

Representative Amazonian species from the Asteraceae, Bignoniaceae, and Caryocaraceae families are shown in Figure 4a–c, providing a comparative overview of plants with distinct morphologies and bioactive profiles. These species exemplify the phytochemical diversity of the Amazon flora, encompassing essential oils, phenolic compounds, and carotenoids with remarkable therapeutic and cosmetic potential.

Pequi pulp is rich in bioactive compounds such as carotenoids, particularly beta-carotene, tocopherols, phenolic compounds, and high concentrations of oleic acid, in addition to omega-3 and omega-6 fatty acids and vitamins A, C, and E [109,110,111,112]. It also contains carotenoids, phenolic acids, hydrolyzable tannins, ellagic acid and its derivatives, and flavonoids, which further contribute to its biological activities [113]. Moreover, pequi exhibits saturated (SFA), monounsaturated (MUFA), and polyunsaturated fatty acid (PUFA) profiles [113,114]. Moreover, pequi exhibits a complex profile of volatile organic compounds such as esters, aldehydes, ketones, and terpenes, particularly sesquiterpenes responsible for its characteristic aroma. These compounds are crucial for its sensory profile and its applications in the food and cosmetic industries [112].

Several studies have reported that pequi pulp and oil exhibit antioxidant, anti-inflammatory, gastroprotective, wound healing, and potential cardioprotective activities [115]. Its anti-inflammatory effects have been demonstrated in models of atherosclerosis, ulcerative colitis, pleuritis, and pulmonary inflammation [116,117]. Its influence on inflammatory markers has also been evaluated in murine models of systemic lupus erythematosus [118] and in studies on insulin resistance [119]. In agriculture, pequi essential oil has shown nematicidal activity against *Meloidogyne javanica*, representing a sustainable alternative to pest control [120].

Regarding biotechnological applications, emulsions loaded with pequi oil have been designed for topical cosmetic products [112], and a biomaterial composed of collagen, gelatin, and pequi oil has been developed to reduce foreign body responses and stimulate collagen production, thereby improving biocompatibility and accelerating wound healing in tissue engineering applications [121]. Protein hydrolysates from pequi almonds have also been produced via ultrasound-assisted hydrolysis, exhibiting functional properties such as solubility and foaming capacity, along with antioxidant activity, which are of interest for the food and nutraceutical industries. Additionally, the microencapsulation of pequi’s carotenoids by complex coacervation using gelatin and gum arabic has been explored to enhance stability against heat, light, and oxygen, increasing their preservation and potential applications in food and nutraceutical formulations [122]. Furthermore, pequi shell extracts have been investigated as natural photosensitizers for antimicrobial photodynamic therapy (aPDT) [123].

### 2.5. Euphorbiaceae

**Figure 5:**
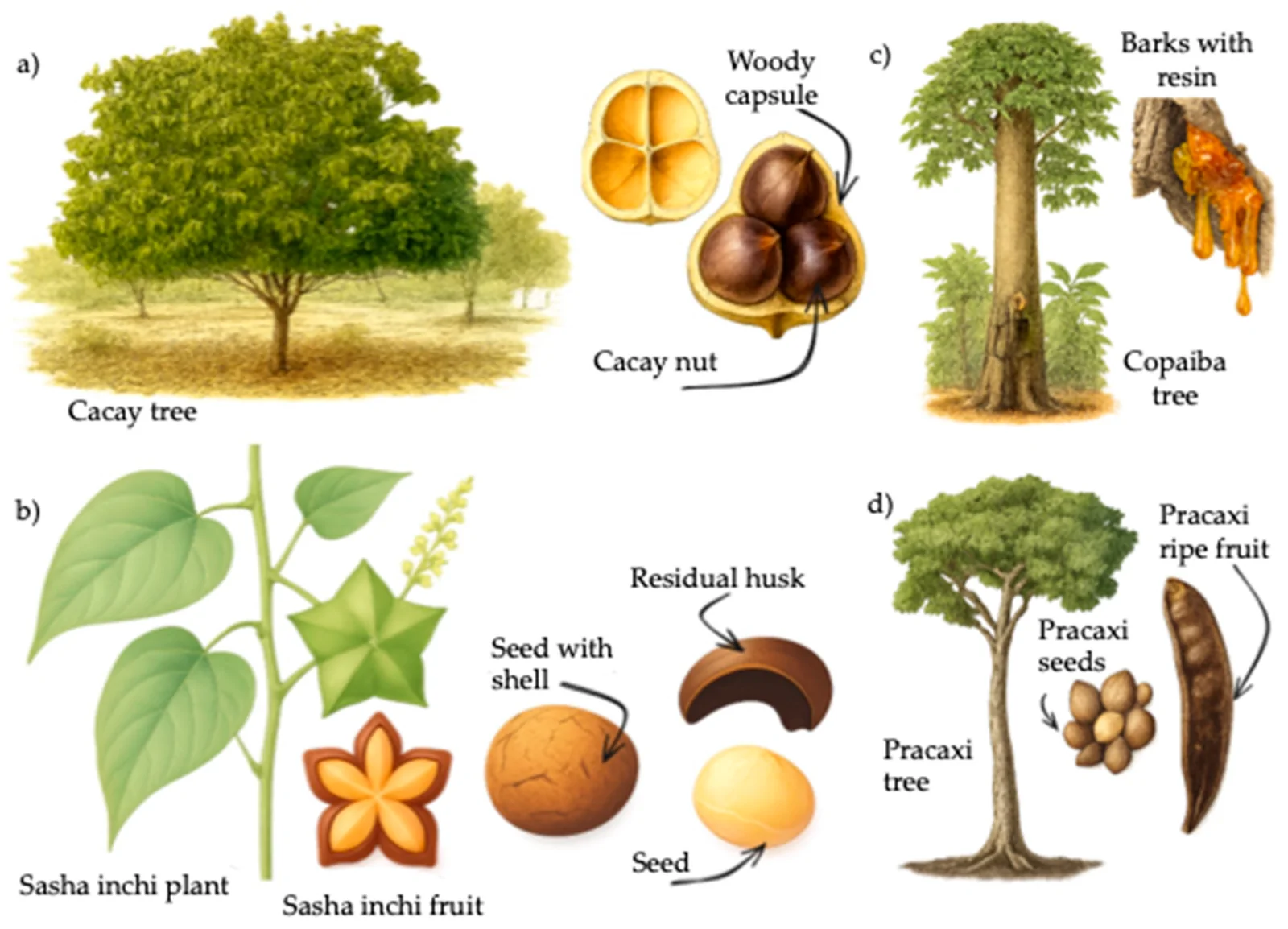
Representative Amazonian species from the Euphorbiaceae and Fabaceae families: (**a**) *Caryodendron orinocense* (cacay), (**b**) *Plukenetia volubilis* (sacha inchi), (**c**) *Copaifera* L. (copaíba) and (**d**) *Pentaclethra macroloba* (pracaxi). Illustrations generated using the Illustrae platform. See the Acknowledgments for details.

Two species of the family Euphorbiaceae are presented (Figure 5a,b): cacay (*Caryodendron orinocense*) and sacha inchi (*Plukenetia volubilis*).

#### 2.5.1. Cacay (*Caryodendron orinocense*)

*Caryodendron orinocense*, commonly known as cacay, is a tree belonging to the Euphorbiaceae family that can reach approximately 20 m in height. Its fruits grow at the ends of branches and contain seeds from which cacay oil is extracted. Traditionally, this oil has been utilized in ethnobotanical practices as an option for wound treatment [124].

The antioxidant and antimicrobial activities of cacay oil were evaluated by de Azevedo et al. [125], who compared it with coconut oil and cacay butter, a commercial product composed of 70% coconut oil. Cacay oil exhibited the highest total phenolic content and antioxidant activity among the samples tested. Additionally, it demonstrated potential to inhibit the growth of Gram-positive bacteria. The authors highlighted that this oil has potential applications in skincare and topical formulations aimed at skin repair. In terms of lipid composition, linoleic acid was the most abundant polyunsaturated fatty acid, representing the major constituent of the lipid composition of cacay oil. This composition of polyunsaturated fatty acids represents good potential for pharmaceutical and cosmetic products.

#### 2.5.2. Sacha Inchi (*Plukenetia volubilis*)

It is important to note that the common name “pracaxi” may refer to different species depending on the region. In Andean–Amazonian regions, the term is also used to refer to *Plukenetia volubilis*, known as sacha inchi, a climbing plant notable for its high content of unsaturated fatty acids. However, in Brazil, the name pracaxi is mainly associated with the tree *Pentaclethra macroloba*, valued for its oil rich in behenic acid. Therefore, both species will be addressed separately.

*Plukenetia volubilis*, commonly known as pracaxi or sacha inchi, is a climbing plant native to the tropical regions of South America, especially the Amazon areas of Peru, Brazil, and Colombia [126]. It belongs to the Euphorbiaceae family and is characterized as a semi-woody species that reaches approximately two meters in height, with vigorous growth and the ability to produce both flowers and fruits [127].

Its fruit is a star-shaped capsule measuring 3 to 5 cm in diameter, which turns from green to dark brown as it matures. Each capsule contains between 4 and 6 oval seeds, dark brown in color and about 1.5 to 2 cm in length [128]. Once processed, these seeds are edible and highly valued for their lipid profile. On average, the dried capsule is composed of 30–35% shell (non-edible) and 65–70% seeds (edible portion) [129].

Among the reported bioactive compounds are ω-3 and ω-6 fatty acids, which explain the use of its oil as a dietary supplement. It also contains tocopherols and phytosterols, compounds with antimicrobial activity, including effects on the adherence of *Staphylococcus aureus* to human skin explants and keratinocytes, which may be beneficial in the treatment of skin lesions [130]. Furthermore, supplementation with α-linolenic acid has been shown to reduce tumor growth [131].

The presence of various phenolic compounds has also been reported, known for their antioxidant, anti-inflammatory, antiproliferative, analgesic, and immunomodulatory activities, thereby contributing to their overall health benefits [132,133].

### 2.6. Fabaceae

Figure 5c,d show the species of the family Fabaceae: copaíba (*Copaifera* L.) and pracaxi (*Pentaclethra macroloba*).

#### 2.6.1. Copaíba (*Copaifera* L.)

The genus *Copaifera* L., commonly known as copaiba, includes several species of trees native to South America, particularly the Amazon region, although they are also found in Central America and parts of the Caribbean. These trees belong to the Fabaceae family (subfamily Caesalpinioideae) and can reach heights ranging from 20 to 40 m. They grow primarily in humid tropical forests, both in non-flooded forest areas and in seasonally flooded regions [134].

One of the main products obtained from these trees is the oil resin known as copaiba balsam, which is extracted by making incisions in the trunk. This product has been widely used in traditional medicine and has various therapeutic applications. Gomes et al. [135] found that copaiba oleoresin exhibits a complex profile largely dominated by sesquiterpenes, with 29 compounds identified by GC–MS. Trans-caryophyllene is the major constituent, accounting for 35.5% of the total composition, followed by α-bergamotene (7.3%), α-humulene (7.0%), and γ-muurolene (7.7%). These components are widely recognized for their anti-inflammatory, antioxidant, analgesic, and antimicrobial activities, supporting the therapeutic relevance of the extract. The high proportion of sesquiterpenes imparts notable characteristics to the oleoresin, including marked hydrophobicity and relative chemical stability, which favor its incorporation into biomaterial systems. For example, it has been considered suitable for topical, oral, and transmucosal applications, particularly in systems designed for rapid or modulated release of bioactive compounds [135,136]. In addition to its therapeutic applications, copaiba oil is also widely used in the cosmetic industry due to its medicinal properties and the characteristic aroma of its essential oils [137]. Currently, it is used as a fixative in perfumes and scented soaps. Thanks to its emollient, bactericidal, and anti-inflammatory properties, copaiba oil is incorporated into the formulation of products such as soaps, lotions, moisturizers, bath foams, shampoos, and conditioners. Furthermore, its use has proven effective in treating conditions such as dandruff and acne [138,139].

#### 2.6.2. Pracaxi (*Pentaclethra macroloba*)

*Pentaclethra macroloba*, commonly known as pracaxi, pracachy, paracaxi, gavilán, or koloballi depending on the country, is a plant species native to the Amazon region, including Brazil, Guyana, and parts of Central and South America. This tree, from the Fabaceae family (subfamily Mimosoideae), reaches an average height of 14 m. It is one of the most abundant species in the Amazon River estuary [140]. The fruits are green pods that, when dried and matured, turn brown and dehisce naturally. The seeds are dark brown with a matte surface, showing shallow indentations that form prominent lines, sometimes with a reticulated pattern, particularly near the base [141].

The bark of pracaxi is traditionally used for tanning hides and, medicinally, in the treatment of diseases such as dysentery, diarrhea, and various microbial infections [142]. The oil extracted from its seeds is notable for its high content of behenic acid, a long-chain fatty acid with emollient, moisturizing, and regenerating properties, making it a valuable ingredient for the cosmetic and pharmaceutical industries. Additionally, its rich composition in oleic and linoleic acids provides antioxidant and anti-inflammatory effects [11,143]. Pracaxi oil has also been reported to contain high levels of β- and γ-tocopherols, as well as other tocopherols and phenolic compounds, which contribute to its antioxidant activity [144,145,146].

### 2.7. Lecythidaceae

#### Brazil Nut (*Bertholletia excelsa*)

**Figure 6:**
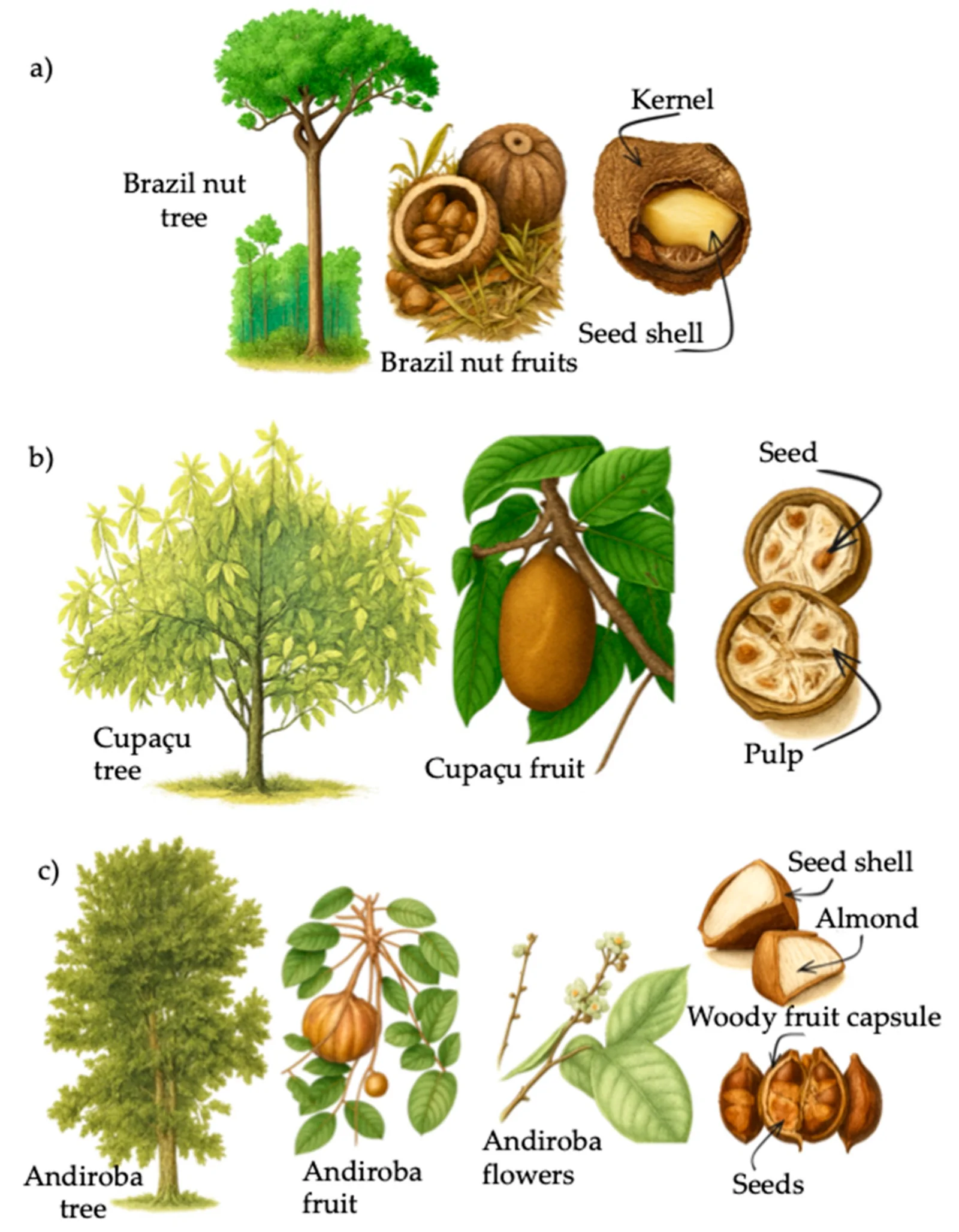
Representative Amazonian tree species from the Lecythidaceae, Malvaceae, and Meliaceae families: (**a**) *Bertholletia excelsa* (Brazil nut); (**b**) *Theobroma grandiflorum* (cupuaçu); (**c**) *Carapa guianensis* (andiroba). Illustrations generated using the Illustrae platform. See the Acknowledgments for details.

The Brazil nut is a commercially important product in Brazil, obtained from the *Bertholletia excelsa*. Its consumption is often associated with a reduced risk of cardiovascular diseases and type 2 diabetes. In addition to the commercialization of the whole nut, its oil can be extracted, typically using cold or expeller pressing methods. Even its by-product, Brazil nut cake, has been shown to be a potential source of phenolic acids and flavonoids, exhibiting antioxidant activity [147]. Figure 6a shows a species of the family Lecythidaceae: the Brazil nut (*B. excelsa*).

Beyond phenolic compounds, Brazil nuts contain phytosterols and tocopherols, with phenolics being the most abundant—present in higher concentrations than in other nuts such as almonds, cashews, and macadamia nuts [148]. Brazil nuts have also demonstrated nutrigenomic modulation capacity over antioxidant enzyme genes, yielding beneficial effects on superoxide-hydrogen peroxide imbalance [149]. Its oil has been studied by Filogônio et al. [150] for improving a commercial dentifrice for biofilm control, where supplementation with Brazil nut oil achieved a result statistically superior to the group treated with just the commercial dentifrice.

### 2.8. Malvaceae

#### Cupuaçu (*Theobroma grandiflorum*)

Cupuaçu (*Theobroma grandiflorum*) is a tropical tree species belonging to the Malvaceae family, native to the Brazilian Amazon, although it is also found in other regions of the Amazon basin, including Peru, Colombia, and Bolivia [151]. The fruit of cupuaçu is large and oblong, with an oval or ellipsoidal shape, its epicarp is rigid, woody, and dark brown when ripe. Inside, it contains a white, aromatic, and bittersweet pulp that surrounds numerous large seeds. Figure 6b shows a species of the family Malvaceae: cupuaçu (*T. grandiflorum*).

The pulp is highly valued for its content of bioactive compounds such as polyphenols, phenolic acids, fatty acids, and theobromine, and it is commonly used in the production of juices, ice creams, sweets, and other products [152,153]. Several studies have reported that the pulp exhibits biological activities such as skin regenerative potential, antioxidant, anti-inflammatory, antimicrobial, and antihypertensive actions, as well as psychostimulant, antidiabetic, anticancer, and cancer immunotherapy-enhancing properties [19,153,154,155,156,157,158].

Likewise, cupuaçu has a rich profile of volatile organic compounds, including organic acids, alcohols, esters, ketones, pyrazines, and terpenes, which are responsible for its characteristic aroma [159]. These compounds can be used as alternative ingredients to provide a chocolate flavor in confectionery products [160].

Biotechnological applications using the biopolymer maltodextrin have also been reported, involving encapsulation through spray drying—a useful technique for preserving bioactive compounds from Amazonian fruits such as cupuaçu [161].

In addition to its value as a superfood, cupuaçu also shows great potential in the development of innovative by-products. Barbalho et al. [19] utilized cupuaçu residues, specifically the seeds, to obtain an extract that stimulates cell proliferation and induces a gene expression profile associated with tissue repair in human dermal fibroblast cultures, highlighting its potential for skin regeneration.

### 2.9. Meliaceae

#### Andiroba (*Carapa guianensis*)

*Carapa guianensis*, commonly known as andiroba, is a tree of the Meliaceae family native to the Amazon region and widely distributed in Brazil, Peru, Colombia, and Venezuela [162]. It can reach up to 30 m in height and typically grows in flood-prone areas or near bodies of water, where it plays an important role in tropical humid ecosystems [163] (Figure 6c). Its seeds are enclosed in woody, valvate-dehiscent capsules that open at maturity to release between one and six seeds [28]. The seeds are bulky and heavy, with a high oil content that gives them a dense, waxy appearance when cut [164].

This oily characteristic form the basis for the extraction of the well-known andiroba oil, one of the most valued products derived from the species. Its composition is rich in unsaturated fatty acids, such as oleic, linoleic, and palmitic acids, as well as limonoid compounds like andirobin, which confer anti-inflammatory, healing, repellent, and analgesic properties [165]. In addition, terpenoids have also been identified in the flowers of andiroba, broadening the spectrum of bioactive metabolites associated with the species [166,167]. For this reason, andiroba oil is widely used in traditional Amazonian medicine to treat wounds, inflammation, muscle pain, and various skin conditions [40]. Similarly, other Amazonian oils, such as copaiba, have also shown anti-inflammatory activity, as demonstrated by their ability to suppress inflammation in asthmatic lungs of BALB/c mice induced with ovalbumin [168].

From a biological potential standpoint, several studies have investigated the use of *C. guianensis* extracts in pharmaceutical and cosmetic formulations, particularly due to their ability to promote collagen synthesis in normal human dermal fibroblasts [169]. Its antimicrobial, antifungal, and larvicidal activities have also been explored [170,171], along with the presence of a wide variety of fatty acids that confer therapeutic properties such as acaricidal, larvicidal, and insect-repellent activity [172].

Additionally, its traditional use has been documented for treating inflammation, contusions, and bone-related conditions [173], as well as its antiplasmodial activity against the *Plasmodium falciparum* K1 strain [174], and cytotoxic activity against leukemia cell lines [175]. Andiroba oil has also been used in biopolymer matrices such as nanoemulsions, designed to reduce the side effects of the antineoplastic drug doxorubicin [176] and incorporated into PCL (polycaprolactone) polymeric films with the aim of developing materials with potential applications in tissue engineering and regenerative medicine [177].

In the context of mucosal treatment, the study by Wanzeler et al. [178] evaluated the therapeutic effect of andiroba oil (*Carapa guianensis* Aubl.) in the treatment of oral mucositis induced by 5-fluorouracil (5-FU) in hamsters. Formulations containing natural andiroba oil at concentrations of 100% and 10%, as well as a refined version at 10%, were tested with other components composed of pectin, gelatin, nipagin, ecgonine methyl ester (EME), and purified water. The results indicated that the emulsion with 100% andiroba oil provided the best recovery from mucositis, reducing inflammation, hyperemia, and ulcerations over the 15-day treatment period.

Figure 6a–c present three emblematic Amazonian species, Brazil nut, cupuaçu, and andiroba, whose seeds and pulps are valuable sources of fatty acids, tocopherols, and phenolic compounds. Together, they exemplify the multifunctionality of Amazonian tree-derived bioactives for food, cosmetic, and biomedical applications.

### 2.10. Myristicaceae

#### Ucuuba (*Virola surinamensis*)

**Figure 7:**
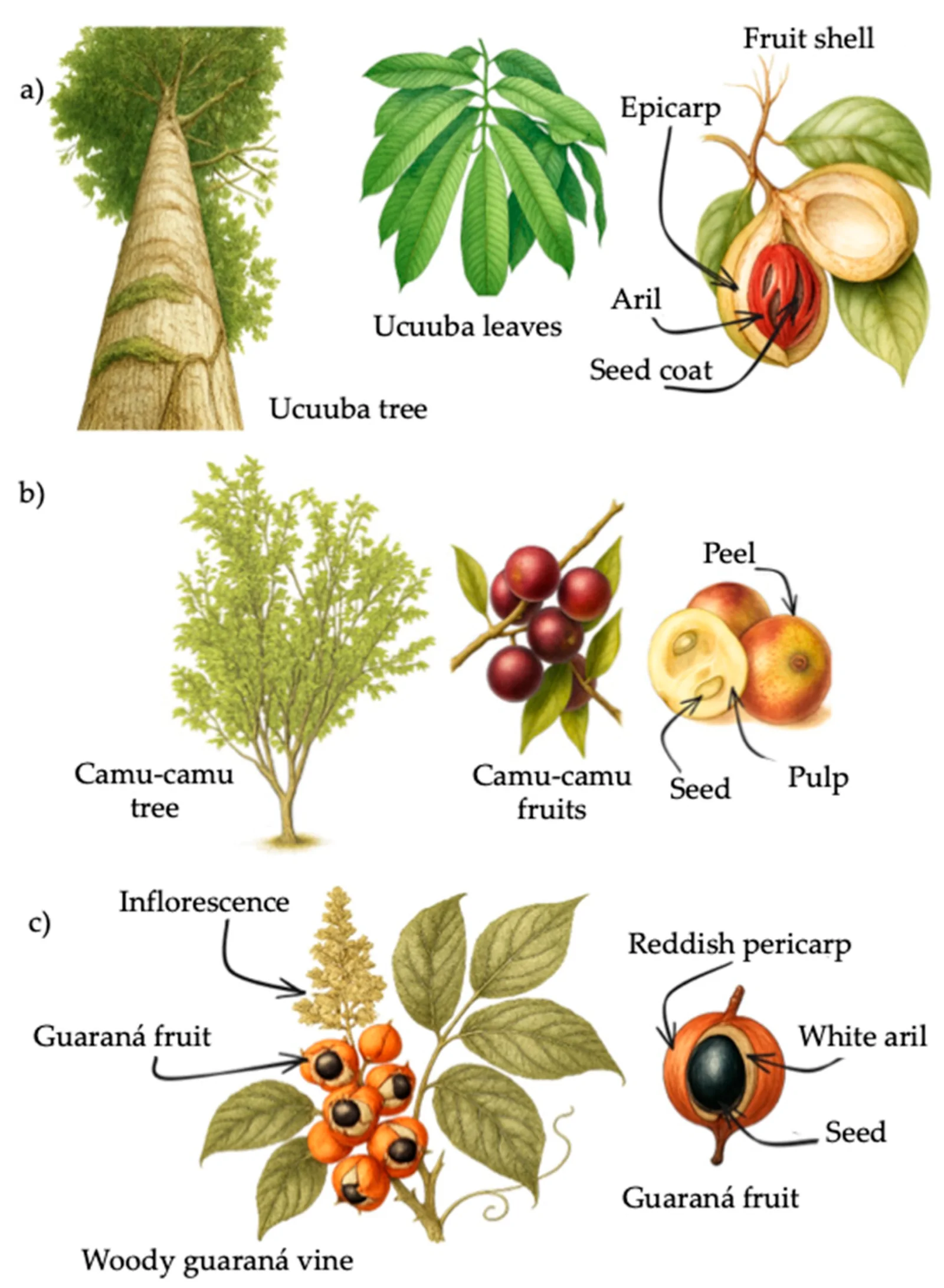
Representative Amazonian fruit-bearing species from the Myristicaceae, Myrtaceae, and Sapindaceae families: (**a**) *Virola surinamensis* (ucuuba), (**b**) *Myrciaria dubia* (camu-camu) and (**c**) *Paullinia cupana* (guaraná). Illustrations generated using the Illustrae platform. See the Acknowledgments for details.

The *Virola surinamensis*, commonly known as ucuuba, belongs to the Myristicaceae family. It is a tree that can be found in the riverbank and seasonally flooded region. Hiruma-Lima et al. [179] reported that this plant is used in the popular medicine to treat diseases in the gastrointestinal tract, besides having antimalarial and tryponosomicidal activities. The authors evaluated the antiulcerogenic activity of an extract obtained from the resin of *V. surinamensis* using in vivo models and confirmed its gastroprotective activities, probably due to the epicatechin present in the extract. Figure 7a shows the species ucuuba (*V. surinamensis*) of the family Myristicaceae.

Veiga et al. [180] highlighted the leishmanicidal activity of *V. surinamensis*’s leaves, demonstrating antipromastigote activity against *Leishmania amazonensis* and *Leishmania chagasi*. The authors attribute the activity to a synergic effect between neoligans and minor constituents of the extract. Paes et al. [52] reached similar results, but demonstrated that the (-)-5 demethoxygrandisin B present in the leaves of *V. surinamensis* as a molecule that presented potential to inhibit *L. amazonensis* in its promastigote and intracellular amastigote forms.

### 2.11. Myrtaceae

#### Camu-camu (*Myrciaria dúbia*)

*Myrciaria dubia*, commonly known as camu-camu, caçari, araçá, azedinha, algracia, guayabillo blanco, guayabito, and limoncillo, belongs to the Myrtaceae family and is native to northern Brazil, as well as neighboring countries such as Peru, Colombia, Bolivia, and Venezuela [42,181]. The camu-camu fruit has a round, globular berry shape, with an approximate diameter of 2.5 cm. When ripe, its skin takes on a color ranging from reddish-brown to dark purple. The pulp is soft and contains 2 to 4 kidney-shaped seeds. Its taste is intensely acidic and astringent, which characterizes it as a very sour fruit [182]. Figure 7b shows camu-camu (*M. dubia*), a species of the family Myrtaceae.

Nutritionally, camu-camu stands out for its high vitamin C content, with concentrations ranging from 2000 to 6500 mg per 100 g of pulp [183,184]. It also provides other beneficial compounds such as beta-carotene, phenolic acids, flavonoids, and anthocyanins. These bioactive compounds contribute to a variety of biological activities, including antioxidant, anti-obesity, antidiabetic, anticancer, anti-inflammatory, antihypertensive, antibacterial, cardioprotective, neuroprotective, and antihyperglycemic properties [185,186,187,188,189].

The biotechnological applications of the bioactive compounds from *M. dubia* include microencapsulation through spray drying, which enables the preservation of the components and their controlled release, with potential application in the nutraceutical industry—for example, as prebiotics for the treatment of periodontal diseases [190,191,192]. Its incorporation has also been highlighted in the development of antioxidant packaging materials, which show improved free radical scavenging capacity and UV-blocking properties, making them useful for the food industry [193].

### 2.12. Sapindaceae

#### Guaraná (*Paullinia cupana*)

*Paullinia cupana*, commonly known as guaraná, is a climbing plant of the Sapindaceae family native to the Amazon region, particularly northern Brazil, with Maués considered its point of origin [194]. Its fruit is a reddish capsule that opens upon ripening to reveal one to three rounded seeds, which are the parts used industrially (Figure 7c). These seeds are rich in methylxanthines, mainly caffeine (also referred to as guaranine), as well as theobromine and theophylline, compounds known for their stimulant effect on the central nervous system [12].

In addition to methylxanthines, other bioactive compounds such as phenolic acids, flavonols, and carotenoids have been identified in guaraná, contributing to its antioxidant, hepatoprotective, and antibacterial properties [195,196,197]. The importance of preserving and effectively delivering these compounds within the human body has driven the development of strategies such as the co-encapsulation of probiotics and guaraná extracts via complex coacervation, using gelatin and gum arabic, aiming for their controlled release in the gastrointestinal tract [198].

The species illustrated in Figure 7, ucuuba, camu-camu, and guaraná, highlight the extraordinary variety of Amazonian fruits containing high-value bioactive compounds, ranging from polyphenols and vitamins to alkaloids and fatty acids. Collectively, these examples emphasize the metabolic versatility of Amazonian flora.

## 3. Application of Amazonian Bioactives in Biopolymeric Matrices

**Figure 8:**
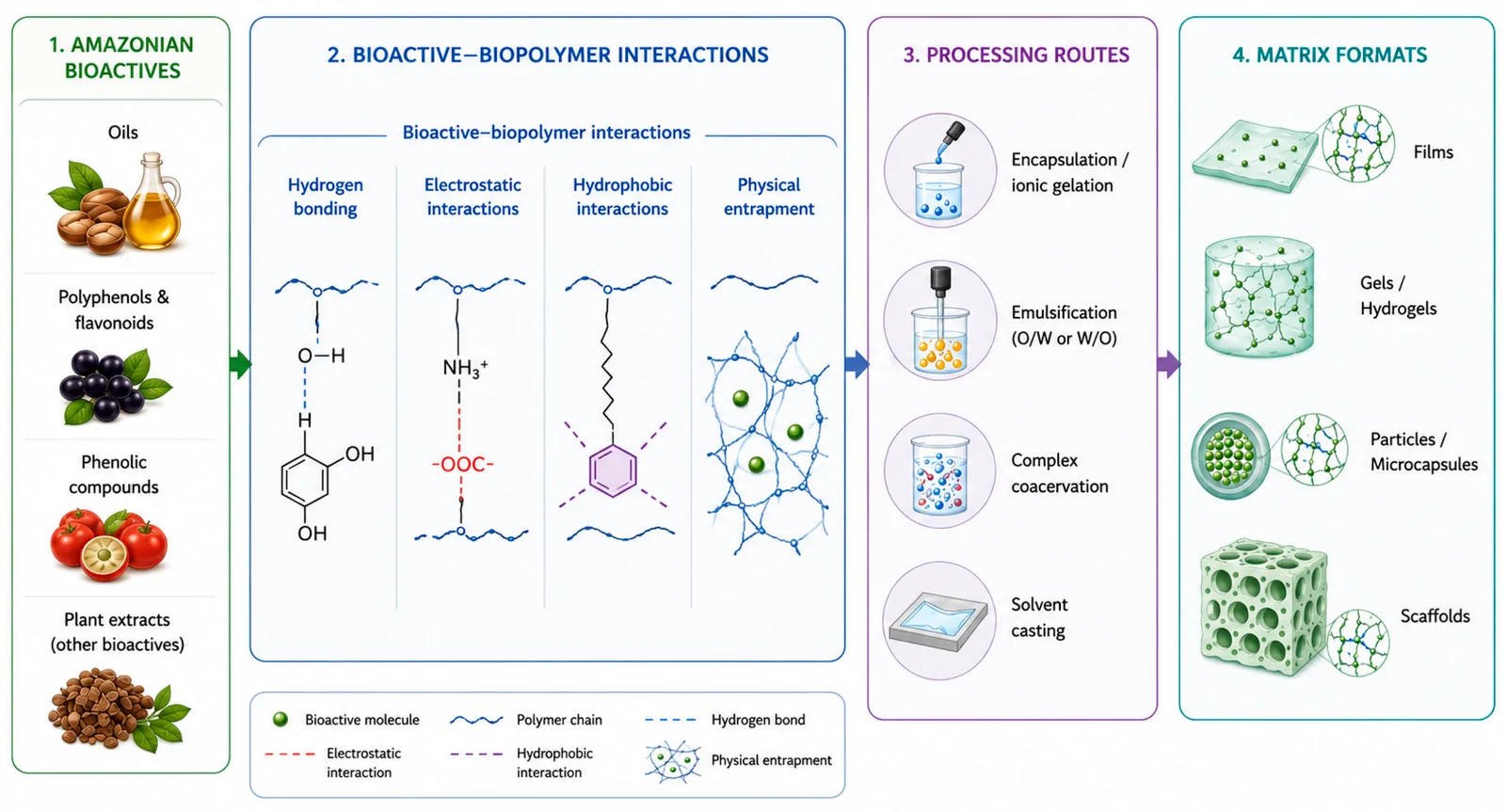
Schematic representation of different application formats for biopolymers. Image generated with assistance of OpenAI ChatGPT-5.6 Luna. See the Acknowledgments for details.

With global population growth and increasing demands to address both environmental and biomedical challenges, biomaterials derived from natural sources have gained considerable attention, particularly in the medical and healthcare sectors [199]. In this context, biopolymers have emerged as promising raw materials for the development of innovative products, owing to their biocompatibility, biodegradability, and tunable physicochemical properties. These biopolymer-based materials have been developed using different formulation strategies, including encapsulation [18,19], emulsification [40,71,176], and complex coacervation [198], resulting in a wide variety of structural formats, such as films [16,21,45,200,201], gels [20], nanostructures [202], microcapsules [81,83,203], and mucoadhesive matrices [135,204]. These systems have been successfully applied in controlled drug delivery [37], wound healing and regenerative applications [16,45], and in the design of biomaterials for orthopedic and dental purposes [25], as illustrated in Figure 8.

Biopolymers are macromolecules produced by living organisms. Their origins include animals, fungi, bacteria, plants, and even biological macromolecules such as ribonucleic acid (RNA) and deoxyribonucleic acid (DNA) [199,205,206,207].

This diversity of sources provides biopolymers with a wide range of structural and functional variations. Naturally biodegradable and biocompatible, they play essential roles in living organisms, such as energy storage, genetic information transmission, and cell structure formation, while also exhibiting great potential for technological applications, particularly in drug transport and controlled release systems [205].

In general, biopolymers are classified into three main groups—polysaccharides, proteins, and polyesters—which differ in their origin and method of production. Polysaccharides are macromolecules composed of repeating units of simple sugars and are widely extracted from plants, algae, and microorganisms. Among their main representatives is starch, obtained from grains and tubers such as corn and cassava, and composed of two other polysaccharides—amylopectin and amylose—which are made exclusively of D-glucose monomers [208]. Pectin, in turn, is isolated from the cell walls of plants and natural fibers, being a polysaccharide that is highly stable from both chemical and mechanical perspectives [209]. Gum arabic and chicá gum are natural exudates from tropical trees, whereas chitosan is obtained through the deacetylation of chitin found in crustacean shells. Another relevant example is alginate, obtained mainly from brown seaweeds, although it can also be synthesized by bacteria of the genera *Azotobacter* and *Pseudomonas*. This biopolymer is composed of varying proportions of two monomeric units, β-D-mannuronic acid (M block) and α-L-guluronic acid (G block), whose combination determines its structural and rheological properties [207].

Proteins, in turn, are natural heteropolymers composed of long chains of amino acids and are mainly derived from animal tissues and insects [210]. Among the most commonly used are gelatin, obtained by the hydrolysis of collagen from skin and bones, often employed as a multifunctional carrier for the delivery of cell growth factors [211], and silk fibroin, composed of predominantly hydrophobic amino acid chains and extracted from the cocoons of the silkworm (*Bombyx mori*), widely applied in biomaterials due to its high biocompatibility and ability to form three-dimensional matrices [212].

Polyesters are polymers formed by the linkage of biologically derived acids and alcohols. They can be synthesized through microbial fermentation routes, as in the case of polyhydroxybutyrate (PHB), or obtained by polycondensation processes that employ acids and diols from plant sources [209].

Together, these three classes of biopolymers provide a versatile and sustainable foundation for the development of functional systems. Table 1 summarizes examples of biopolymer-based systems combined with Amazonian bioactives, designed for topical, oral, bone tissue, and transmucosal applications.

Recent research has focused on incorporating bioactive compounds of plant and microbial origin into biopolymeric systems [219]. These bioactives not only enhance the mechanical and chemical properties of biomaterials but also introduce functional attributes such as antimicrobial and antioxidant activity, thereby expanding their applicability in biomedical and pharmaceutical contexts [17,42,220].

The Amazon rainforest, known for its vast biodiversity, represents a particularly rich source of bioactive compounds, as discussed in Section 2. Amazonian plants, fruits, and microorganisms have attracted growing interest due to their safety profiles, environmental sustainability, and consumer acceptance [200,215]. Their well-documented biological activities [219] make them ideal candidates for incorporation into biopolymeric matrices.

This section presents recent advances in the use of Amazonian bioactives in biopolymer-based biomaterials, highlighting their impact on both mechanical and biological performance. These include improvements in mechanical strength, flexibility, thermal stability, and biodegradability, along with enhanced antimicrobial, antioxidant, and anti-inflammatory effects. Together, these properties support the development of multifunctional and therapeutically effective biomaterials for biomedical applications [47].

### 3.1. Gels

Gels are semi-solid systems in which the organization of the polymeric or molecular network can influence hydration, mechanical behavior, stability, and the mobility of incorporated compounds. In systems containing Amazonian bioactives, these properties depend not only on the intrinsic biological activity of the incorporated compounds but also on their compatibility with the gel-forming components and on the processing conditions used to generate the three-dimensional network [20]. Consequently, the incorporation of an Amazonian oil or extract may modify the morphology, thermal behavior, crystallinity, rheological properties, and water or oil retention of the resulting system. These structure–property relationships are particularly relevant to biomedical applications such as wound healing and topical delivery.

Ferreira et al. [213] developed a wound healing gel based on chicha gum, chitosan, and buriti oil (*Mauritia flexuosa*), evaluating its antimicrobial, antioxidant, and anti-inflammatory properties. Oil incorporation resulted in a porous network, while the higher thermal stability of the material suggested interactions between the biopolymers and the oil. The results indicated that the gel exhibited a high content of phenolic compounds (81.81 µmol gallic acid/g) and flavonoids (57.92 µmol quercetin/g), contributing to its antioxidant activity (IC_50_ = 0.379 mg/mL). In hyaluronidase enzyme inhibition tests, a significant anti-inflammatory effect was observed, with a 10.17% reduction in enzyme activity for the formulated gel. Additionally, the gel demonstrated antimicrobial activity against *Staphylococcus aureus*, *Klebsiella pneumoniae*, and *Candida albicans*, with a minimum inhibitory concentration (MIC) of 2.50 mg/mL for *S. aureus*, suggesting its potential for infection control in wounds. In vivo assays revealed that treatment with the gel promoted skin re-epithelialization within 21 days, facilitating tissue regeneration and dense collagen formation.

In addition, organogels, a variant of gels, have been extensively explored in the biomedical field, particularly for prolonged drug release, dermal substitutes, and tissue engineering. Organogels (OGs) are viscoelastic semi-solid materials composed of structuring molecules that form a continuous network, in which an apolar liquid phase (usually a natural oil) is immobilized by the three-dimensional network of the structuring agent, resulting in self-sustaining systems [221]. Unlike conventional polymeric hydrogels, however, many organogels are structured by low-molecular-weight organogelators rather than biopolymers. Therefore, their relevance to this review lies primarily in illustrating how the chemical composition of an Amazonian oil can affect molecular self-assembly, crystallization, network organization, and consequently the physicochemical properties of the resulting material. Sanches et al. [20] developed an organogel based on açai oil (*Euterpe oleracea*), structured with 12-hydroxy stearic acid (12-HSA) and incorporated with hyaluronic acid (HA), targeting anti-aging cosmetic applications. In this system, the structural role of the Amazonian oil differs from that observed in hydrophilic polymeric gels because açai oil constitutes the continuous nonpolar phase, whereas 12-HSA forms the three-dimensional organogel network and HA is incorporated as a hydrophilic component. Thus, the study provides evidence of interactions among the oil, organogelator, and HA that influence the organization and mechanical behavior of the system. FTIR and polarized light microscopy indicated the incorporation of HA without disruption of the organogel structure, suggesting interactions involving hydroxyl groups within the structured phase. HA incorporation increased the mechanical strength of the system, with the maximum compression force increasing from approximately 15 to 20 N in the first cycle and from 8 to 15 N in the second cycle. The authors also reported HA retention within the three-dimensional network, with 11.22 µg/mL of cross-linked HA detected. During 90 days of storage, HA concentration remained between 5.19 and 9.08 µg/mL, indicating the retention and stability of HA within the organogel rather than demonstrating a specific release mechanism.

Another study, conducted by Narvaez et al. [37], developed organogels from Amazonian oils of buriti (*Mauritia flexuosa*) and cacay (*Caryodendron orinocense*), focusing on topical applications and investigating how oil composition affects organogel formation, crystallization, and structural properties. Because these organogels were structured with cetostearyl alcohol rather than a biopolymer, the study is better interpreted as evidence of an oil–structuring agent structure–property relationship rather than a direct bioactive–polymer interaction. The organogel formulated with buriti oil demonstrated greater thermal and mechanical stability, with smaller and more uniformly distributed crystals, requiring a lower concentration of gellant (9%) and achieving a high oil retention capacity (99.4%). In contrast, the gel with cacay oil required a higher gellant concentration (11%) and exhibited larger crystals with lower structural cohesion, indicating potential use as a diffusion-controlled release system.

### 3.2. Scaffolds

Scaffolds are three-dimensional structures commonly fabricated using techniques such as particle leaching, lyophilization, and rapid prototyping, aiming to create porous architectures that replicate the extracellular matrix. In tissue engineering, these systems provide both structural and biochemical support for tissue regeneration and are widely used in bone repair, cartilage regeneration, and wound healing [222]. Beyond providing structural support, scaffolds can also regulate cell–material interactions, degradation, and the local availability of bioactive compounds, depending on their composition, architecture, and processing route [218].

Scaffolds are three-dimensional structures widely used in tissue engineering, especially in strategies focused on bone regeneration. These matrices play a fundamental role in various applications, including the induction of endochondral osteogenesis, the stimulation of neovascular formation, controlled drug release, and the delivery of growth factors, even in oncological contexts. Guedes et al. [25]. developed scaffolds based on chitosan, hydroxyapatite, and magnetic particles, demonstrating that this combination can offer the appropriate physicochemical characteristics for biointegration, osteoinduction, and site-specific functionalization. The incorporation of inorganic and magnetic components into the chitosan matrix provides an example of how scaffold composition can be used to modify the physicochemical and functional characteristics of the polymeric network.

To further expand scaffold functionality, Lima et al. [23] developed poly(ε-caprolactone) (PCL) structures via rotary jet spinning (RJS), combined with alginate hydrogels (2, 4, and 6% *w*/*v*) and subsequently immersed in pracaxi oil (*Pentaclethra macroloba*), with a focus on tissue regeneration. The alginate layer was formed through ionic gelation with Ca^2+^, in which hydroxyl and carboxyl groups of alginate interact with the divalent ions to produce the hydrogel network. The materials exhibited a porous and fibrillar morphology, with pore diameters ranging from 9.27 to 37.57 µm. The addition of alginate led to partial pore closure and denser, rougher surfaces, while the incorporation of pracaxi oil reduced crystallinity and altered wettability, indicating changes in the organization of the PCL/alginate system. As a result, multizonal scaffolds were achieved, characterized by hydrophobicity/hydrophilicity gradients across the layers, capable of modulating specific functions in different tissues. This relationship between composition and wettability is particularly relevant for tissue engineering, as differences in surface hydrophilicity can influence hydration and cell–material interactions. However, thermal stability was reduced by the presence of alginate and/or oil (a decrease in the maximum degradation temperature of ~13–59 °C compared to pure PCL), and in vitro assays revealed that although pure PCL was non-cytotoxic, the presence of alginate decreased cell viability (~60–77%). Pracaxi oil did not intensify cytotoxicity but indicated the need for formulation adjustments to ensure greater biocompatibility.

In contrast, Sousa et al. [24] sought to overcome similar limitations by developing bio-based polyurethane (PU) synthesized from polyols derived from buriti oil, combined with hydroxyapatite (HAp) particles, obtaining composite scaffolds via in situ polymerization for bone tissue regeneration. In this system, the role of the Amazonian bioactive phase is fundamentally different from that observed in scaffolds in which an oil is subsequently incorporated. In this synthesis route, buriti oil was chemically modified into a polyol, one of the two essential precursors in polyurethane formation (along with isocyanates), thus becaming part of the polyurethane network itself and contributing to a sustainable polymer network with enhanced biocompatibility. The incorporation of HAp further modifies the organization of the polymer matrix through particle–matrix interactions, affecting pore morphology, density, thermal stability, and degradation behavior. Three formulations were produced (5, 10, and 15% HAp), which exhibited porous structures suitable for osteogenesis, with mean pore diameters ranging from ~135 µm in PU-10, the most uniform, to ~326 µm in PU-15, while PU without HAp showed greater structural irregularity. The incorporation of HAp promoted greater structural uniformity (average pores of ~158 µm in PU-10), increased density (0.424 g cm^−3^ in PU-10 vs. 0.370 g cm^−3^ in PU-0), improved thermal stability (raising the onset of the second degradation stage from ~207 °C in PU-0 to 248–253 °C in the composites), and reduced degradation in PBS after 28 days (32.7% in PU-0 versus 13.8–19.4% in the composites). Thus, increasing HAp content modified the scaffold at multiple structural levels, from pore architecture and density to thermal resistance and degradation rate. Moreover, all systems maintained >95% survival in *Artemia salina* and ≥70% of cell viability, confirming their biocompatibility. The preservation of cell viability despite the incorporation of HAp further indicates that the structural modifications did not compromise the biological compatibility of the composite within the conditions evaluated. Among the formulations, PU-10 stood out as the most promising, combining structural uniformity, thermal resistance, reduced degradation, and maintenance of cell viability.

### 3.3. Particles

Particle-based systems have been widely investigated for the protection and delivery of bioactive compounds, particularly because the composition and structure of the encapsulating matrix can influence bioactive stability, particle formation, and release behavior. Among these, encapsulation is one of the most traditional approaches. The principle of encapsulation lies in isolating the active compound from adverse conditions that could compromise its integrity, either by forming a protective barrier around the substance or by incorporating the bioactive into a continuous polymeric matrix, depending on the technique employed [18,223]. In these systems, the interaction between the bioactive phase and the wall material, together with processing conditions, determines important characteristics such as particle size, morphology, encapsulation efficiency, moisture content, stability, and bioactive release. Several methods can be employed in this process, which are commonly classified into three major groups: physical, chemical, and physicochemical. Physical methods include extrusion, submerged or vibrating nozzles, spray drying, rotary disk, pan coating, air suspension, spray chilling, fluidized bed, co-crystallization, and lyophilization. Chemical methods, in turn, comprise strategies such as interfacial polymerization, molecular inclusion, ionic gelation, and in situ polymerization. Physicochemical methods encompass simple or complex coacervation, liposomes, nanoprecipitation, interfacial deposition, lipospheres, solvent evaporation, and protein self-assembly [224].

In addition to these conventional encapsulation techniques for particle production, an emerging alternative is the green synthesis of nanoparticles, which employs phytochemicals from plant extracts as natural mediators of particle formation, stabilization, and morphological control [225]. In this approach, plant-derived compounds can participate in the reduction and stabilization of precursor materials, thereby influencing particle nucleation, growth, aggregation, and surface characteristics.

**Figure 9:**
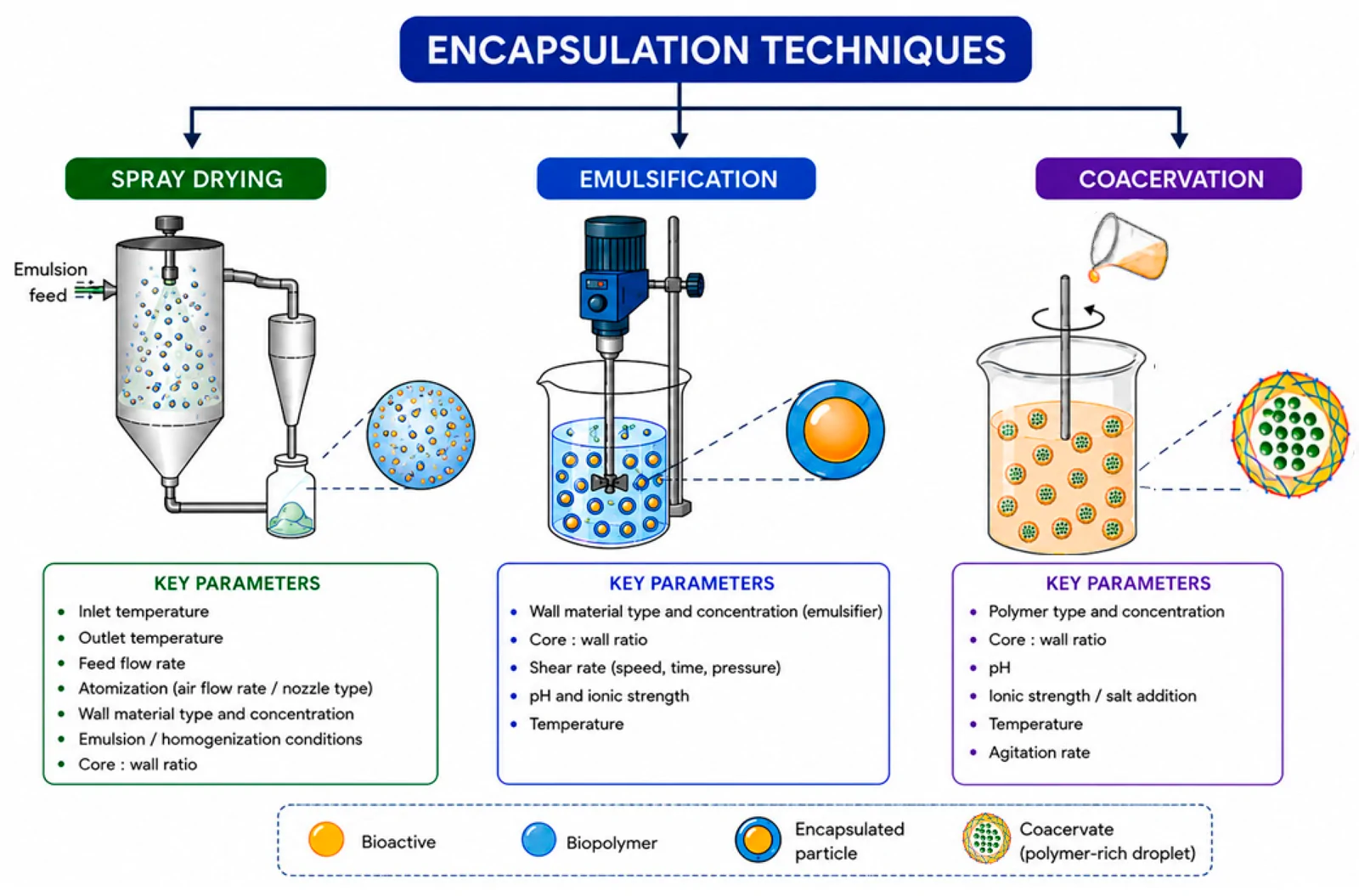
Encapsulation techniques for bioactive compounds. Image generated with assistance of OpenAI ChatGPT-5.6 Luna. See the Acknowledgments for details.

Both approaches expand the range of applications, either by protecting and controlling the release of bioactives or by enabling the production of functional nanoparticles mediated by natural compounds. The following subsections focus on particle-forming strategies (illustrated in Figure 9) in which processing conditions and matrix composition directly influence particle structure and bioactive retention, with emphasis on the relationships among formulation, processing, particle characteristics, and bioactive stability.

Particle spraying techniques encompass well-established methods, such as spray drying, and emerging strategies, such as electrospraying, both aimed at producing micro- or nanoparticles encapsulating bioactive compounds. Spray drying is one of the most established techniques for microencapsulation, distinguished by its relatively low operational cost, high reproducibility, application flexibility, and ease of industrial scale-up, yielding particles of consistent quality [226]. This method is particularly effective for protecting bioactive molecules and probiotics that are highly susceptible to photodegradation, oxidation, and free radical damage [227,228]. The process integrates atomization, drying, and powder collection into a single step, thereby reducing processing time and minimizing the risk of contamination [229]. Typically, the active compound is mixed with the encapsulating agent, and the mixture is atomized into fine droplets that undergo rapid drying to form encapsulated microparticles. During this process, droplet formation and drying kinetics determine the final particle morphology and the distribution of the bioactive within the wall matrix.

Rodríguez-Cortina et al. [230] developed microcapsules of Sacha Inchi oil (*Plukenetia volubilis Linnaeus*), an Amazonian species with high nutritional value. The goal was to protect polyunsaturated fatty acids, particularly omega-3 and omega-6, from oxidation, improving their stability and bioavailability. The study investigated the impact of different homogenization techniques and drying temperatures on the physical and thermal properties, as well as the controlled release of the encapsulated oil. The combination of ultrasound homogenization and a 170 °C inlet temperature provided the most favorable overall characteristics, with high encapsulation efficiency and improved thermal protection, while the microcapsules also exhibited oil release under simulated gastrointestinal conditions. The results highlight the potential of microencapsulation for enhancing Amazonian bioactive compounds, with promising applications in biomaterials.

In a similar study, Acosta-Vega et al. [18] performed the encapsulation of cupuaçu pulp (*Theobroma grandiflorum*) using spray drying, with inulin and maltodextrin as biopolymer matrix. The use of a mixed inulin/maltodextrin is relevant because the composition of the carrier matrix influences both particle formation and the protection of hydrophilic compounds present in the pulp. Under the optimized formulation, the resulting particles exhibited smooth and predominantly spherical morphologies, indicating effective formation of the dried matrix around the pulp components. The formulation showed a yield of 53.29%, reduced moisture content (2.00%), water activity of 0.26, and water solubility index of 88.56%, indicating high stability. Encapsulation preserved 95.52% of ascorbic acid and 62.41% of phenolic compounds, while the gastrointestinal model showed limited ascorbic acid release during the gastric phase (7.1%) followed by a marked increase during the intestinal phase (76.7%). These results indicate that the inulin/maltodextrin matrix influenced the retention and subsequent release of the encapsulated compounds according to the gastrointestinal environment. In the study by Moser et al. [36], buriti oil (*Mauritia flexuosa*) had its stability enhanced through microencapsulation with chickpea protein (CP) and highly methoxylated pectin (HMP) via spray drying. In this system, CP and HMP formed an interfacial complex around the oil droplets, with the protein initially adsorbing at the oil–water interface followed by pectin deposition. The formation of this interfacial layer increased intermolecular interactions within the interface and improved droplet stabilization against coalescence. Consequently, the organization of the CP–HMP interface contributed directly to the encapsulation performance of the spray-dried particles. The CP-HMP formulation showed high encapsulation efficiency (98.4) and preserved the integrity of the bioactives during the process and storage, with minimal oxidation after six months. Thus, the high encapsulation efficiency observed with the CP–HMP system can be related to the formation of a more effective interfacial barrier around buriti oil droplets, which limited exposure of the lipid phase to oxidative conditions during processing and storage.

An alternative approach to the application of spray drying was presented by Santos et al. [27], who combined two atomization processes—spray drying (SD) and spray chilling (SC)—to microencapsulate tucumã oil (*Astrocaryum vulgare* Mart.). In SD, gum arabic was used as the biopolymer matrix, whereas SC employed vegetable fat, resulting in particles with distinct hydrophilic and lipid-based matrices. The combination of both processes (SDC) generated particles with a polymeric core and lipid wall, providing a different structural organization from that obtained by either technique alone. The particles produced by SD exhibited a carotenoid content of 366.0 µg/g with 91% retention, indicating that the lower carotenoid concentration resulted mainly from the lower oil loading of the combined particles rather than extensive carotenoid loss during processing. Importantly, the different particle structures affected their behavior after incorporation into the starch matrix. SEM analysis showed that SDC particles remained intact after extrusion, whereas SD particles were apparently disrupted and more homogeneously integrated into the polymer network. The greater integrity of SDC particles was associated with improved carotenoid stability during storage, suggesting that particle resistance to processing can be an important factor in preserving bioactive compounds during subsequent incorporation into a biopolymer matrix. However, particle integrity also influenced the mechanical response of the final biopolymer matrix. The large SDC particles generated discontinuities and fissures within the cassava starch network, resulting in lower tensile strength and elongation. This finding illustrates that particle stability during processing must be balanced with particle–matrix compatibility, since preservation of the encapsulated bioactive does not necessarily translate into reinforcement of the polymeric material.

Electrospraying, also referred to as electrohydrodynamic spraying, has gained increasing attention as an innovative alternative to conventional drying. In this technique, polymeric solutions are subjected to a strong electric field that overcomes surface tension, forming fine jets which, upon solvent evaporation, generate submicrometer particles, whose size and morphology depend strongly on the electrical and physicochemical properties of the polymeric solution [231]. The applied voltage, solution properties, flow rate, and solvent evaporation rate determine jet stability and therefore influence particle size, morphology, and encapsulation efficiency. Dicastillo et al. [35] applied this approach to the encapsulation of açaí extract (*Euterpe oleracea*) in zein, obtaining submicrometer particles with cavities averaging approximately 924 nm and an encapsulation efficiency of ~72%. FTIR analysis indicated chemical interactions between the açaí polyphenols and zein, supporting the formation of an associated bioactive–protein matrix rather than simple physical mixing. The resulting particle structure provided an effective barrier to thermal degradation: phenolic losses were limited to approximately 5% after sterilization at 121 °C and 20% after oven heating at 180 °C, whereas losses exceeded 40% in the non-encapsulated extract. Moreover, simulated gastrointestinal digestion demonstrated that the bioaccessibility of encapsulated polyphenols was approximately twice as high compared to the free extract. Thus, the combination of zein as the carrier matrix and electrospraying as the particle-forming process influenced both the physicochemical protection of the extract and its subsequent availability during digestion. These findings highlight the potential of electrospraying as a complementary technique to spray drying, particularly when the objective is to combine molecular interactions between bioactive compounds and a protein matrix with rapid particle formation and protection against thermal degradation.

#### 3.3.1. Coacervation

**Figure 10:**
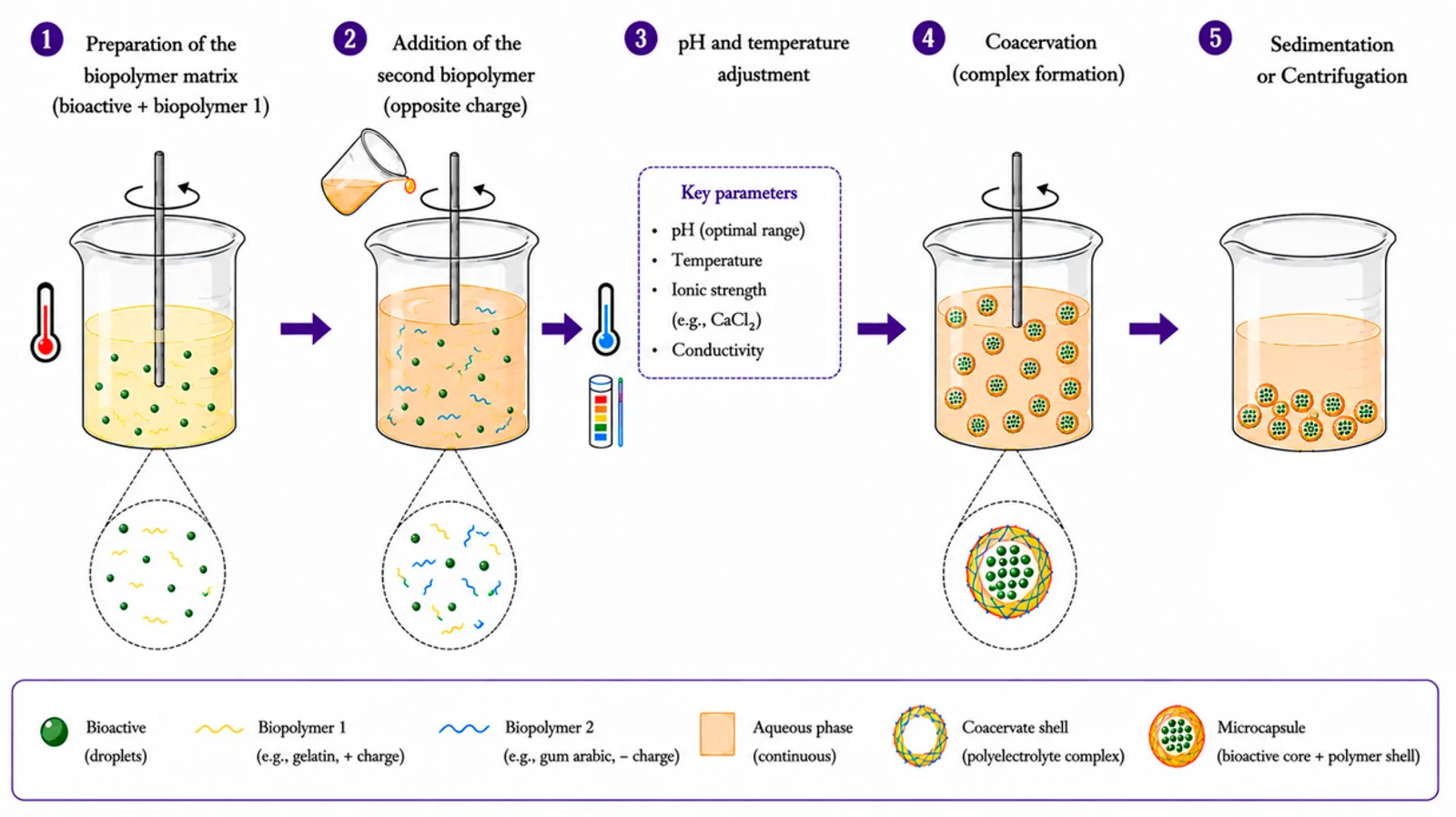
A schematic representation of microencapsulation by complex coacervation in which oppositely charged polymers interact in solution, forming a matrix around the active core and generating microcapsules. Image generated with the assistance of OpenAI ChatGPT-5.6 Luna. See the Acknowledgments for details.

Coacervation is a widely used physicochemical technique for the encapsulation of bioactive compounds, based on the phase separation of colloidal systems induced by electrostatic interactions or by changes in parameters such as pH, temperature, and ionic strength [232]. This methodology can occur in two main forms: simple coacervation, which involves a single polymer and in which phase separation is triggered by the action of an external agent, such as the addition of salts or pH adjustment; and complex coacervation, which arises from the association of at least two oppositely charged polyelectrolytes, such as proteins and polysaccharides. In the latter case, electrostatic interactions between the polymers promote system destabilization and the subsequent formation of a polymer-rich phase, capable of efficiently encapsulating sensitive bioactives [233]. Figure 10 illustrates the process of microencapsulation by complex coacervation.

Although electrostatic interactions play a central role in the association between oppositely charged biopolymers, hydrophobic forces and hydrogen bonding also contribute significantly to the formation of the complexes [234]. The balance among these interactions determines the organization and compactness of the coacervate phase and can consequently affect capsule size, wall structure, encapsulation efficiency, stability, and release behavior. Among the main advantages of coacervation are its high encapsulation efficiency, the absence of organic solvents, and the ability to control the release of bioactives under different physiological conditions, ensuring greater stability and functionality [233].

Teixeira-Costa et al. [38] encapsulated açaí pulp oil (*Euterpe oleracea*) via complex coacervation between chitosan and alginate, using CaCl_2_ as a cross-linking agent, with oil concentrations ranging from 0.25 to 0.75% and salt concentrations from 0 to 1.0%. In this system, the negatively charged carboxylate groups of alginate interact electrostatically with the positively charged amino groups of chitosan, forming a polyelectrolyte complex, while Ca^2+^ provides additional ionic cross-linking of alginate chains. The concentration of both oil and CaCl_2_ therefore influences the organization and properties of the resulting microcapsules. The microcapsules exhibited a gel-like morphology, with an average size above 340 µm and a polydispersity index of up to 1.8, achieving extremely high encapsulation efficiency, above 99%, in all tested formulations. The high encapsulation efficiency indicates that the CS/alginate matrix effectively retained the hydrophobic oil phase, while changes in formulation composition affected the dimensions and rheological characteristics of the capsules. The optimized encapsulated formulation exhibited higher measured antioxidant activity, which rose from approximately 70.5% in the free oil to ~78% in the optimized system (0.5% oil and 0.5% CaCl_2_), whereas the control without oil showed only ~37%. In addition, encapsulation provided greater thermal stability to açaí oil, as evidenced by the shift in the onset of degradation from approximately 220 °C in the free oil to ~250 °C in the encapsulated system, indicating that the polyelectrolyte matrix provided a protective environment for the oil during thermal exposure.

Complementarily, Silva et al. [198] investigated the co-encapsulation of *Paullinia cupana* (guaraná) peel extracts and probiotics using complex coacervation, aiming to increase probiotic viability while simultaneously releasing bioactive compounds in a simulated gastrointestinal tract. The coacervate matrix provided differential retention of the encapsulated compounds during gastrointestinal simulation, with the release profile varying according to the physicochemical characteristics of the bioactive fraction and the digestive environment. The co-encapsulation of probiotics and guaraná extracts in coacervates resulted in a sequential release of bioactive compounds during the simulated digestive process. The release of phenolic compounds was 60% in simulated gastric fluid (SGF), while the release of carotenoids was approximately 13% in SGF and increased significantly after transitioning to simulated intestinal fluid (SIF), reaching 90% release. This difference between phenolics and carotenoids indicates that the matrix can provide distinct retention behavior for compounds with different physicochemical characteristics. Moreover, the viability of probiotics was significantly enhanced in the presence of guaraná peel extract, with a release of approximately 7.2 log CFU/mL at the end of the test, compared to 5–6 log CFU/mL in the nanoemulsions without the extract.

Another relevant example is the study by Barbosa et al. [32], who developed an encapsulation system for sacha inchi oil (*Plukenetia volubilis* L.) through complex coacervation between carboxymethylcellulose (CMC) and lactoferrin (Lf), identifying pH 5.0 and a CMC:Lf ratio of 1:14 as optimal conditions. The formation of the CMC–Lf complex was driven initially by electrostatic attraction between the oppositely charged polymers, as demonstrated by zeta-potential and turbidimetric analyses. Isothermal titration calorimetry further indicated a two-stage interaction process, with electrostatic attraction dominating the initial association and additional interactions, including hydrogen bonding and conformational contributions, becoming relevant during complex formation. The resulting capsules exhibited a spherical morphology with a well-defined core and high encapsulation efficiency, reaching up to 97.24% in the system with 1.5% biopolymers and a core-to-wall ratio of 1:1. The formation of the CMC–Lf complex therefore generated a defined polymer-rich shell around the oil phase, providing a structural barrier capable of retaining the lipophilic core. The release profile of β-carotene followed a Fickian diffusion model, with approximately 30% release within the first 2 min, 61% at 41 min, and stability maintained up to 120 min. This behavior indicates that diffusion through the coacervate matrix was the dominant mechanism under the evaluated conditions, with the polymer network regulating the mobility of β-carotene. During simulated gastrointestinal digestion, β-carotene showed ~70% preservation in the gastric phase, followed by a marked release in the intestinal phase, reaching 84.31% release and 67.86% bioaccessibility. Furthermore, the encapsulated compound remained 87.42% stable after digestion, showing that the pH-dependent behavior of the CMC–Lf matrix contributed to differential retention during gastric conditions and greater release under intestinal conditions.

More recently, coacervation has also been applied to the development of capsules containing copaiba oil (*Copaifera* spp.). In this case, the technique was combined with an initial nanoemulsification step, followed by complex coacervation between chitosan and gelatin. This sequential strategy separates particle formation into two structural stages: first, nanoemulsification reduces the oil phase to submicrometric droplets and promotes their stabilization by gelatin; subsequently, chitosan interacts with the gelatin-stabilized droplets to form the coacervate layer. Thus, droplet size and surface charge generated during nanoemulsification become important determinants of the subsequent coacervation process. The nanoemulsion exhibited an average droplet diameter of 281 nm, PdI of 0.29, and zeta potential of –25.6 mV, ensuring adequate dispersion and colloidal stability. Subsequent coacervation produced spherical polynucleated polymeric capsules with an average size between 20 and 35 µm and high encapsulation yields of ~89–90%. The transition from submicrometric oil droplets to micrometer-scale coacervate capsules illustrates how the initial interfacial organization of the oil phase can be transferred to a larger polymeric structure during complex coacervation. The chitosan:gelatin ratio was also a critical parameter governing coacervate formation and yield. Encapsulation enhanced thermal stability, shifting the onset of degradation from ~180–200 °C in the free oil to ~230–240 °C in the encapsulated system, in addition to improving photo-stability, with 85–90% of the chemical composition preserved after light exposure, in contrast to the pronounced losses observed in the free oil. These results indicate that the coacervate shell provided an effective physical barrier against thermal and light-induced degradation of the oil constituents.

#### 3.3.2. Emulsions

Emulsions play a crucial role in the incorporation of bioactives in biomedical and pharmaceutical systems, enhancing stability, bioavailability, and controlled release of bioactive compounds [10,235]. As vehicles for drugs [236], cosmetics, and biomaterials, they enable the homogeneous dispersion of oily extracts and compounds in aqueous media, facilitating their absorption and therapeutic efficacy [237,238]. This occurs because emulsions overcome the limitations of other systems, offering ease of spreading, simple removal, emollient properties, water solubility, and providing a pleasant feel and attractive esthetic appearance. Furthermore, emulsions have a long shelf life and are effective in drug permeation through the skin, facilitating controlled delivery of active substances [204,239].

Given their versatility, various studies have explored emulsions and nanoemulsions formulated with bioactive extracts, with notable examples including those based on andiroba oil (*Carapa guianensis Aubl*.) [178], buriti (*Mauritia flexuosa*), pequi (*Caryocar brasiliense*) [240], passion fruit (*Passiflora edulis*), açaí (*Euterpe oleracea*) [200], among others. These systems illustrate how emulsification can modify the physical environment of Amazonian oils and extracts by reducing the dispersed-phase dimensions and creating an interfacial layer between the bioactive phase and the continuous medium. Consequently, droplet size, interfacial composition, and colloidal stability can influence the protection, distribution, and availability of the incorporated compounds. These studies have shown positive impacts on wound healing, tissue regeneration, and inflammation control, although the contribution of the emulsion structure to these biological effects depends on the specific formulation and application.

In contrast, Soares et al. [241] evaluated the effects of a 3% andiroba oralbase emulsion, formulated with pectin, gelatin, nipagin, carboxymethylcellulose, and purified water, on the symptoms and progression of oral mucositis in children with leukemia undergoing chemotherapy, comparing it to treatment with low-level laser therapy. The formulation combined gelatin and pectin with carboxymethylcellulose, providing a polymer-containing aqueous vehicle for the dispersion and topical application of andiroba oil. The results indicated that the group treated with andiroba showed better clinical outcomes, with a significant reduction in pain by the eighth day and complete absence of symptoms by the ninth day of follow-up. This positive effect was attributed to the analgesic and antimicrobial properties of andiroba oil, which exerts inhibitory action on the microbiota and modulates the inflammatory process, as well as the presence of tetranortriterpenoids (TNTPs) extracted from *C. guianensis* seeds.

Moraes et al. [214] evaluated nanoencapsulation of buriti oil (*Mauritia flexuosa* L.F.) in porcine gelatin, investigating its cytotoxicity, antioxidant potential, and effect on the modulation of antibiotic activity. Nanoencapsulation was carried out using the oil/water emulsification technique followed by lyophilization, and the resulting nanoparticles were characterized in terms of particle size, encapsulation efficiency, and thermal stability. The emulsification step generated the dispersed oil phase within the gelatin-containing aqueous phase, while subsequent lyophilization converted the emulsion into a dry particulate system. Thus, droplet formation during emulsification and the organization of gelatin around the oil phase are important for the resulting particle structure and protection of the encapsulated compounds. The results demonstrated that the nanoformulation enhanced the antioxidant potential and showed no cytotoxicity in CHO (Chinese Hamster Ovary) cells. Regarding the modulation of antibiotic activity, the nanoencapsulated buriti oil exhibited a synergistic effect with norfloxacin and gentamicin, reducing the minimum inhibitory concentration (MIC) against *Escherichia coli* and *Pseudomonas aeruginosa* by 98% and 99%, respectively. For *S. aureus*, the combination with norfloxacin reduced the MIC by 50%, suggesting that buriti oil may act as a modulator of bacterial resistance.

In the treatment and control of vectors, emulsions are also utilized. The study by Sarquis et al. [172] investigated the use of andiroba oil (*Carapa guianensis*) combined with silk fibroin to prepare emulsions with larvicidal activity against *Aedes aegypti*. The emulsions were made with andiroba oils and fatty acid derivatives, using silk fibroin as an emulsifier. The use of silk fibroin as the emulsifying component is relevant because the protein is positioned at the oil–water interface, helping to disperse the hydrophobic oil phase in the aqueous medium. The resulting interfacial organization can influence droplet stability and the availability of oil-derived compounds in the surrounding aqueous phase. The results showed that the emulsion with ethyl fatty acid esters and silk fibroin had the lowest lethal concentration (LC_50_ = 16.79 mg/mL) after 48 h of exposure. Additionally, emulsions with silk fibroin exhibited lower interfacial stability but improved the distribution and bioactivity of the bioactive compounds. Thus, although greater interfacial stability was not necessarily achieved, the emulsified state improved the distribution of the hydrophobic components, which was reflected in the biological activity of the formulation. These findings indicate that silk fibroin could be an efficient and eco-friendly alternative for encapsulating vegetable oils, expanding the use of natural oils as biopesticides.

In addition to nanoencapsulation approaches, another promising strategy is co-encapsulation, which involves the combination of different bioactive compounds in a single system, allowing for the simultaneous protection and potential modulation of their release. This technique has been explored in various studies to optimize the stability and therapeutic efficacy of bioactives. One such example is the study by Comunian et al. [242], which investigated the co-encapsulation of pequi (*Caryocar brasiliense*) and buriti (*Mauritia flexuosa*) oils in oil-in-water emulsions, followed by lyophilization, using whey protein isolate (WPI), both in its natural and heated forms, as an emulsifier. In this system, WPI acted at the oil–water interface, and its structural state influenced the organization and stability of the dispersed droplets. The results demonstrated that the emulsions exhibited adequate stability, with an average droplet diameter ranging from 0.88 to 2.33 µm, with formulations containing heated WPI showing the lowest instability indices. The smaller droplet size and improved physical stability obtained with the optimized formulations increased the protection of the carotenoid-rich oil phase during storage. After 30 days of storage at 37 °C, the co-encapsulated formulations retained 50 and 48% of the total carotenoids, while the free oils showed significantly lower retention (31%). Additionally, the oxidative stability indices were 51 h and 46 h for the oils co-encapsulated with natural and heated WPI, respectively, compared to 20.5 h for the free oils. These results demonstrate a direct relationship between interfacial stabilization, emulsion structure, and the preservation of oil-derived bioactives during storage.

#### 3.3.3. Physicochemical Encapsulation Methods

Physicochemical encapsulation methods involve processes in which molecular recognition, self-assembly, precipitation, or interfacial deposition led to the formation of organized structures capable of carrying bioactive compounds. One example is liposomes, vesicles formed by phospholipid bilayers that can simultaneously incorporate both hydrophilic and lipophilic molecules. In the study with guaraná powder, obtained by the reverse-phase evaporation method, a low initial encapsulation efficiency of caffeine (17.02%), a hydrophilic compound, was observed; however, this value gradually increased to 30.13% after 90 days of storage. In contrast, lipophilic compounds such as catechin and epicatechin exhibited high encapsulation efficiencies (74.34% and 87.53%, respectively), which remained significant even after 90 days [31].

Another physicochemical approach is molecular inclusion using cyclodextrins, cyclic oligosaccharides whose hydrophobic cavities can accommodate lipophilic compounds while their hydrophilic outer surfaces favor dispersion in aqueous environments. This host–guest organization can protect hydrophobic bioactives from environmental degradation and modify their accessibility to the surrounding medium. Cid-Samamed et al. [235] reported that cyclodextrin could protect açai oil (*Euterpe oleracea*) from photo-oxidation under gastrointestinal conditions. Similarly, Magalhães et al. [10] evaluated inclusion complexes between açai oil and cyclodextrin and found greater antimicrobial activity than that of the isolated oil, with lower MIC values against *S. aureus*, *E. coli*, *E. faecalis*, and *P. aeruginosa*. The inclusion complexes also showed a synergistic effect with ampicillin against *E. coli*, whereas their combination with piperacillin/tazobactam did not significantly modify antimicrobial activity.

Similarly, the nanoprecipitation of preformed polymers is another widely used strategy, in which the diffusion of miscible solvents promotes the controlled precipitation of the polymer and the formation of nanocapsules. Nascimento et al. [243] developed and characterized nanocapsules of tucumã oil (*Astrocaryum vulgare*), evaluating its antioxidant activity, phytochemical composition, and antiproliferative effect on mammary adenocarcinoma cells (MCF-7). The nanoencapsulation was performed by precipitation of the preformed polymer, using poly(ε-caprolactone) (PCL) as the wall polymer, and the resulting nanocapsules had an average size of 206 nm, a zeta potential of −15.4 mV, and a pH of 6.43, indicating colloidal stability. Antioxidant assays demonstrated high free radical scavenging activity in the DPPH (IC50 = 8.59 µg/mL) and ABTS (TEAC = 2.99 µmol trolox/mg) tests. In cytotoxicity tests, tucumã oil nanocapsules exhibited an antiproliferative effect superior to free oil, with an IC50 of 50 µg/mL for the nanocapsules and 130 µg/mL for the free oil in MCF-7 cells. The increased efficacy was attributed to the improved permeability and cellular retention of the nanoencapsulated oil, allowing for the sustained release of bioactive compounds.

Another related physicochemical technique is the interfacial deposition of preformed polymers, which differs from classical emulsification. This method relies on the diffusion of miscible solvents, followed by the deposition of the polymer at the oil/water interface, leading to the formation of nanocapsules or nanoparticles. Barbalho et al. [19] demonstrated this process by encapsulating *Theobroma grandiflorum* (cupuaçu) seed extract into chitosan-coated nanocapsules. The system not only ensured the safety of the extract but also stimulated cell proliferation and the expression of genes associated with tissue repair in human dermal fibroblasts, highlighting its potential for formulations aimed at wound healing and skin regeneration.

Finally, noteworthy is the self-assembly of amphiphilic proteins, such as silk fibroin, which also falls within physicochemical encapsulation methods. Marinho et al. [26] explored the bioactive potential of andiroba (*Carapa guianensis*) and Brazil nut (*Bertholletia excelsa*) oils. The authors incorporated butyl esters derived from these oils into silk fibroin nanoparticles to evaluate their effectiveness in controlling the *Aedes aegypti* vector, a major arbovirus transmitter of significant epidemiological importance. The nanoparticles exhibited particle sizes ranging from 207 nm to 540.8 nm, with zeta potentials between −37.9 mV and −62.9 mV, indicating good colloidal stability. The formulation with the nanoparticles demonstrated high larvicidal efficacy against *Aedes aegypti*, with an LC50 of 27.45 µg/mL for the nanoparticles with andiroba oil and an LC50 of 21.14 µg/mL for the nanoparticles with Brazil nut oil after 48 h of exposure. Moreover, the nanoparticles exhibited repellent effects, inhibiting mosquito oviposition, with a reduction in oviposition rates of up to 93%, suggesting that the incorporation of Amazonian oils into silk fibroin nanoparticles enhances biological activity and could be an effective strategy for vector control, as well as for applications in biotechnology and public health.

#### 3.3.4. Green Synthesis of Particles

In addition to conventional encapsulation methods, a promising alternative for the development of nanometric systems is the green synthesis of metallic nanoparticles. In this approach, plant extracts act as natural mediators, promoting ionic reduction, nucleation, and particle stabilization [225]. Various plant parts—leaves, stems, roots, flowers, fruit pulp, and seeds—as well as associated endophytic microorganisms, represent abundant sources of phytoconstituents capable of functioning as reducing agents [29]. During this process, phytochemicals present in the extracts can participate in the reduction of metal ions and subsequently adsorb onto the nanoparticle surface, contributing to particle stabilization and influencing nucleation, growth, aggregation, and morphology.

Recent studies have demonstrated that this strategy is simple, cost-effective, and environmentally sustainable, while also showing high efficiency in nanoparticle production [244]. Phytochemicals such as polyphenols, flavonoids, and alkaloids not only influence particle growth and morphology but also confer intrinsic biological functionalities, including antioxidant, antimicrobial, and antiproliferative activities. Therefore, the biological response of green-synthesized nanoparticles may arise from the combined effects of the inorganic nanoparticle and the phytochemical compounds associated with its surface. Thus, green synthesis provides an interface between nanotechnology and green chemistry, standing out over conventional physicochemical methods by eliminating or reducing the need for some synthetic reducing and stabilizing agents and leveraging abundant natural molecules as eco-friendly stabilizers [30].

Within the context of Amazonian biodiversity, this approach becomes even more relevant, as plant species from the region have proven to be potential sources of metabolites capable of mediating the formation of functional nanoparticles, thereby broadening the prospects for applications in biomedicine, cosmetics, and other bioactive delivery systems.

In this context, the study by Vieira et al. [245] involved the synthesis of zinc oxide (ZnO) nanoparticles from açai (*Euterpe oleracea*) seed waste extract, aiming to investigate their antioxidant, antimicrobial, and anticancer properties. The phytochemical profile of the seed extract identified flavonoids, alkaloids, terpenoids, and other metabolites, which were proposed to participate in the reduction and stabilization of ZnO during synthesis. In particular, the authors proposed that hydroxyl groups of flavonoids interact with Zn(II), contributing to the formation of an intermediate zinc–phytochemical complex prior to ZnO formation during calcination. FTIR analysis supported the involvement of functional groups from the plant extract in the synthesis, while XPS detected a small carbon fraction (2.01%) on the nanoparticle surface, indicating the presence of residual plant-derived components after synthesis. The resulting ZnO exhibited a hexagonal wurtzite structure, spherical morphology, an average particle size of approximately 60 ± 5.4 nm by TEM, a hydrodynamic diameter of 75 ± 12 nm, and a zeta potential of −26.58 ± 8.2 mV, indicating a negatively charged aqueous dispersion. These structural characteristics were accompanied by measurable antioxidant and antimicrobial activities. The ZnO NPs showed 56% DPPH radical scavenging activity at 1000 µg/mL (EC50 = 868.67 ± 90.25 µg/mL) and activity against both Gram-positive and Gram-negative bacteria. In vitro cytotoxicity was also observed against A431 squamous carcinoma cells and HaCaT keratinocytes, with IC50 values of 59.50 and 57.58 µg/mL, respectively; therefore, these results demonstrate biological activity but do not by themselves establish therapeutic anticancer efficacy.

### 3.4. Films

The incorporation of bioactives into biopolymer films has gained prominence in the development of functional materials for various applications, particularly in the pharmaceutical, cosmetic, and food industries [246,247]. These films, composed of biopolymers, provide a matrix in which the incorporation of bioactive compounds can modify hydration, mechanical behavior, barrier properties, and mass transport [248]. The final properties depend not only on the intrinsic characteristics of the bioactive, but also on its concentration, dispersion within the polymeric matrix, and the interactions established with polymer chains. Thus, films can be designed to balance structural integrity with the diffusion or retention of incorporated compounds, depending on the intended application.

Biopolymers such as starch, pectin, gelatin, chitosan, and alginate provide chemically and structurally distinct matrices for incorporating Amazonian extracts and oils. Their different hydrophilicities, chain structures, and intermolecular interactions can influence the dispersion of bioactives and, consequently, the hydration, mechanical behavior, barrier properties, and release of the resulting films [201,249].

In this sense, the study by Ferreira et al. [21] investigated the synthesis and characterization of biopolymeric films based on chitosan, green banana peel extract, and andiroba oil (*Carapa guianensis*) for application in epithelial lesions. The films exhibited good swelling capacity and high moisture retention, with hydrophilic properties. Furthermore, they demonstrated significant antimicrobial activity against *S. aureus* and *E. coli*, with MIC values of 2.50 mg/mL and 2.00 mg/mL, respectively. In vivo assays showed that the films promoted re-epithelialization and dense collagen formation within 21 days, providing in vivo evidence of tissue response to the formulated material. The study by Paranhos et al. [17] evaluated the effect of incorporating different concentrations of copaiba oil (*Copaifera langsdorffii*) (0.1–5% *v*/*v*) into chitosan membranes for potential application in wound healing. The results showed that oil addition significantly altered the structural and physicochemical properties of the polymeric matrix. The effect was concentration-dependent: at low concentrations (0.1% and 0.5%), the membranes exhibited higher fluid absorption capacity, reaching 214% and 230% in phosphate-buffered saline (PBS) after 14 days, along with good handling properties. At higher oil concentrations, however, the formation of dispersed oil droplets within the chitosan matrix (up to 123.8 µm at 1%) was accompanied by reduced fluid absorption (90% and 86% at 1% and 5%, respectively) and structural fragility, indicating that excessive incorporation can disrupt matrix organization. Contact angle analysis revealed variations in hydrophilicity, with the 1% oil formulation being more hydrophilic (contact angle—38.3°) compared to the control (contact angle—66.7°), while moisture content decreased from 21.3% in the control to approximately 9.9% in the film with 1% oil. These changes indicate that incorporation of the oil altered both surface wettability and water retention, properties that are relevant to the interaction of the film with aqueous wound environments. Furthermore, XRD analyses indicated that the presence of copaiba oil promoted a transition from a semicrystalline to an amorphous structure.

A similar trend was reported by Gomes et al. [135], who incorporated copaiba oleoresin (0.5%, 1%, and 3.5%) into chitosan-based films and likewise observed marked alterations in physicochemical behavior. The incorporation of the oleoresin increased film mass (0.025–0.041 g) without substantially affecting thickness (0.27–0.33 mm) and progressively reduced moisture content (15.24–20.23%), consistent with the enhanced hydrophobicity conferred by the sesquiterpene-rich extract. Swelling behavior was formulation-dependent: the 1% COR film exhibited the highest water uptake in the initial hours, whereas the 3.5% formulation showed the lowest swelling and greatest resistance to hydration. Solubility remained extremely high across all formulations (94.1–96.7%), indicating rapid aqueous disintegration. Together, the studies by Paranhos et al. [17] and Gomes et al. [135] demonstrate that the concentration and physical dispersion of hydrophobic Amazonian oils within chitosan matrices can substantially modify hydration and surface properties, with intermediate compositions providing a different balance between water uptake and matrix integrity than higher oil concentrations.

Pectin, widely studied in the production of biopolymeric films, is a polysaccharide found in the cell wall and intercellular regions of plants and fruits [250]. Pectin films offer good oxygen barrier properties, hardness, and adhesiveness, but have limitations such as brittleness, rigidity, water sensitivity, and high water vapor permeability [251]. The incorporation of hydrophobic bioactives through emulsification provides an alternative to direct incorporation by modifying their dispersion within the hydrophilic polymer matrix. In this context, the incorporation of bioactives in pectin films by emulsification can improve the properties of these films by enhancing their stability, flexibility, and resistance to moisture [252]. In this context, the work by Norcino et al. [216] developed pectin films containing copaíba oil nanoemulsions, which conferred notable bioactive and functional properties to the material. The films exhibited enhanced flexibility (elongation at break increased from 1.7% to 2.4%) and reduced water vapor permeability by about 60%, indicating improved barrier performance. Additionally, the presence of the oil increased the contact angle, suggesting higher hydrophobicity, and imparted antimicrobial activity against *E. coli* and *S. aureus*. Although the incorporation of copaíba oil slightly decreased the thermal stability (onset temperature from 215 °C to 200 °C), the films remained biodegradable and maintained their functional integrity. These results illustrate how the use of a nanoemulsified oil phase can simultaneously modify mechanical, surface, and barrier properties of a pectin matrix, rather than simply transferring the biological activity of the oil to the film.

Still in the field of therapeutic applications, the study by Silva et al. [177] investigated hybrid films of poly(ε-caprolactone) (PCL) incorporated with andiroba oil for wound treatment applications. The results showed that the incorporation of andiroba oil increased the thermal stability of the films, with higher degradation temperatures compared to pure PCL films, indicating that andiroba oil acts as a barrier, improving thermal stability. Additionally, the hybrid films exhibited greater hydrophobicity, with contact angles ranging from 80.26° (control) to 91.39° (PCL-2.7). The hybrid samples demonstrated good biocompatibility, showing no toxicity in L929 cells, and exhibited good fluid absorption, indicating that oil incorporation altered thermal and interfacial properties without compromising the evaluated cellular response.

In the study by Pires et al. [45], flexible and porous membranes composed of chitosan and alginate, incorporated with standardized Verlot extract (*Arrabidaea chica*), were developed for the treatment of skin lesions. The membranes showed good physical and mechanical stability, with the addition of *A. chica* extract not negatively affecting the properties of the films. The membranes with the extract showed an incorporation efficiency of over 87% and controlled release of the extract, with release kinetics characterized by Fickian diffusion (n values below 0.5 in the Korsmeyer–Peppas model), indicating that diffusion through the polymeric matrix was the predominant mechanism under the tested conditions.

Continuing this research, Concha et al. [33] evaluated chitosan/alginate membranes incorporated with 10% (*w*/*w*) of standardized *Arrabidaea chica* extract, demonstrating that matrix architecture directly affected mass transport. Dense membranes (CA and CAS) showed higher cumulative release of the extract (up to 100% within 24 h), whereas porous membranes (CAP and CAPS) exhibited faster release but in lower total amounts. Kinetic analysis indicated that all systems followed a Fickian diffusion mechanism, with Korsmeyer–Peppas n values below 0.5 and Weibull β parameters associated with diffusion in fractal matrices or highly disordered structures. Thus, even when the same bioactive and polymeric components were used, changes in membrane architecture altered both the rate and extent of release, demonstrating that release behavior can be tuned through matrix morphology rather than solely through bioactive concentration.

In addition to plant extracts, Matos et al. [253] also investigated cupuaçu (*Theobroma grandiflorum*) extracts in microbial films, reporting changes in film micromorphology following extract incorporation. This study further illustrates that bioactive incorporation can modify film architecture, although the available evidence is more limited regarding the relationship between these structural changes and biological performance. In line with this finding, Romani et al. [215] reported that incorporation of whole açaí seed flour (*Euterpe oleracea*) into rice starch films imparted antioxidant activity to the resulting films. At concentrations up to 15% (*w*/*w*), the incorporation increased ABTS radical scavenging capacity and reducing power, while lower concentrations (5%) already provided measurable antioxidant activity and improved distribution and cohesion within the polymer matrix.

The chemical nature of Amazonian bioactives can also influence the structural organization and functional properties of biopolymer matrices. Moura et al. [201], in a review focused on *Acmella oleracea* (jambu) and biodegradable films, compiled evidence on compounds occurring in jambu and related film systems. Among the compounds identified in jambu, β-sitosterol, limonene, ascorbic acid, phenolic compounds, and carotenoids have been associated with distinct effects on polymeric materials. For example, β-sitosterol has been reported to decrease water vapor permeability while increasing the proportion of ordered crystalline domains, contributing to a more compact film structure. Similarly, limonene can increase elongation at break while improving resistance to water penetration, indicating a plasticizing effect associated with the incorporation of a hydrophobic compound. Ascorbic acid, in turn, has been associated with increased crystallinity and hydrophilicity in starch-based films. These observations illustrate that the effect of a bioactive on a polymer matrix is not determined solely by its biological activity, but also by its chemical structure and affinity for the polymeric phase, which can modify chain organization, crystallinity, hydration, mechanical behavior, and barrier properties.

In addition to stability, release behavior is a critical parameter in the design of biomedical films. Garcia et al. [217] developed oral disintegrating films (ODFs) based on gelatin and starch incorporating camu-camu powder (*Myrciaria dubia* McVaugh). Although the films exhibited rapid in vitro disintegration, 10.3 s for the formulation with higher starch content (80% starch/20% gelatin) compared with 20.4 s for films composed of 100% gelatin, the rapid disintegration indicates that the formulation was designed for prompt rather than sustained release. Nevertheless, films with a higher starch content showed improved vitamin C retention, with approximately 44% reduction after 39 days under adverse storage conditions (75% relative humidity and 40 °C), showing that polymer composition affected both disintegration behavior and vitamin C stability during storage.

Bioactives can be incorporated into biopolymeric films either through direct incorporation or via encapsulation, including nanoencapsulation and nanoemulsions, [252] among others. Encapsulation is one strategy used to modulate the release and stability of bioactives incorporated into biopolymeric films. Encapsulation can modify the spatial distribution of the bioactive within the film and introduce an additional diffusion barrier between the compound and the surrounding medium. In this context, Alkilani et al. [254] demonstrated that emulsification was crucial to improving the drug release profile in hydroxypropylmethylcellulose and chitosan films, resulting in an approximately 2.5-fold increase in permeation flux and nearly a 3-fold higher permeability coefficient compared to films prepared through direct incorporation. Furthermore, the use of emulsification reduced the initial burst release effect and promoted a more gradual and sustained release over 24 h. Reinforcing the argument of matrix synergy, Barbosa et al. [32] confirmed that the incorporation of encapsulated drug (nanoemulsion) resulted in a 2.4-fold increase in the diffusion coefficient and nearly a threefold increase in permeation flux compared to films with direct drug incorporation. In addition, the nanoemulsion-based films exhibited a more sustained release profile, extending beyond 24 h. Together, these studies demonstrate that the physical organization of the bioactive within the film can substantially modify mass transport: compared with direct incorporation, emulsified or encapsulated forms altered diffusion and permeation while reducing the initial burst release.

Candido et al. [255] employed the synergy of bionanocomposites and nanoemulsions as an effective strategy for incorporating pracaxi oil (*Pentaclethra macroloba*) into biopolymeric films. Pracaxi oil, being rich in phenolic compounds and fatty acids, is highly susceptible to oxidation and degradation when directly incorporated; however, its inclusion in a nanoemulsion significantly reduced instability, resulting in greater retention of total phenolic compounds (an increase of approximately 35%) and enhanced antioxidant activity, as measured by DPPH, β-carotene/linoleic acid, and phosphomolybdenum assays. At the same time, the combination with pectin–montmorillonite bionanocomposites promoted improved thermal stability, a reduction of about 22% in water vapor permeability, and increased mechanical strength of the films. Thus, the nanoemulsion provided a more protective form of oil incorporation, while the bionanocomposite matrix contributed to barrier and mechanical performance, illustrating the complementary roles of bioactive organization and polymer architecture.

In the same perspective, Santos et al. [27] encapsulated tucumã oil (*Astrocaryum vulgare* Mart.) and incorporated the microparticles into cassava starch extruded films. The films enriched with the encapsulated oil exhibited significant improvements in mechanical properties, with up to a 42% increase in tensile strength and an approximate 28% reduction in water vapor permeability compared to control films without oil. Moreover, the presence of the microparticles promoted greater retention of carotenoids and phenolic compounds, while preserving the antioxidant activity during storage. Pinto et al. [256] investigated chitosan films (CSF) incorporated with copaiba essential oil (CO) for the treatment of bacterial skin infections. To overcome the limitations of direct oil incorporation, CO was previously encapsulated into polymeric nanocapsules (CO-NC) obtained via nanoprecipitation. These nanocapsules exhibited diameters below 250 nm, low polydispersity (PdI < 0.3), a negative surface charge, and encapsulation efficiency above 70%. Direct incorporation of CO into the films (CO-CSF) compromised the homogeneity and surface properties of the material. In contrast, incorporation of CO-NC (CO-NC-CSF) preserved film quality and showed a plasticizing effect in mechanical analyses. Together, these studies show that encapsulation can alter not only bioactive retention but also the compatibility of hydrophobic compounds with polymer matrices, improving film homogeneity and, in some cases, mechanical and barrier properties.

Regarding the use of microbial-derived extracts from the Amazon region, Falcão et al. [16] developed cassava starch-based films incorporating phenolic compounds extracted from the Amazonian endophytic fungus *Aspergillus niger*, isolated from *Myrcia guianensis*. The extract exhibited a high total phenolic content (1311 mg GAE/100 g) and significant antioxidant activity (89.88 mmol Q/g in the FRAP test), maintaining bioactivity after film release. Approximately 74% of the compounds were released within 25 min at 37 °C and pH 6.8. The films demonstrated 86.4% cellular antioxidant activity, higher than the positive control (quercetin, 85.7%), and showed no significant cytotoxicity in human fibroblasts (cell viability of 85.4%). The mechanical, thermal, and solubility properties of the film were not significantly altered by the incorporation of the extract. This study therefore provides evidence at both the material and cellular levels, although the rapid release observed under the tested conditions indicates that the system was not designed for prolonged delivery.

## 4. Future Trends and Challenges

Although the incorporation of Amazonian bioactives into biopolymeric matrices has shown significant advances in recent years, several scientific and technological challenges still need to be addressed to enable the large-scale development of functional biomaterials with clinical and industrial applicability. One of the main challenges is related to the instability of Amazonian bioactive compounds, such as polyphenols, carotenoids, tocopherols, and unsaturated fatty acids, which are highly sensitive to light, temperature, and oxidation [18,36,230]. This chemical instability requires the use of controlled encapsulation techniques, such as spray drying, emulsification, and complex coacervation, which do not always ensure full preservation of biological activity, especially under conditions of high temperature or extreme pH [35,230].

In addition, biopolymers commonly used as matrices, including chitosan, alginate, pectin, gelatin, and starch, although biodegradable and biocompatible, often exhibit mechanical, thermal, and processing limitations, which restrict their direct application [107,207,213]. Their natural heterogeneity also leads to batch-to-batch variability, since properties such as molecular weight, degree of crystallinity, and substitution depend on the biological source and extraction method [207]. These factors directly affect the stability, uniformity, and release efficiency of hybrid systems.

The development of surface modification, cross-linking, and multilayer encapsulation strategies has been identified as a promising approach to overcome these limitations and expand the use of biopolymers as functional carriers for Amazonian bioactives [107,207]. Nevertheless, chemical interactions between phenolic or lipidic compounds and polymeric chains may alter critical properties such as viscosity, porosity, and crystallinity, leading to undesirable release profiles [19,43]. Furthermore, the difference in polarity between hydrophobic compounds and hydrophilic matrices can reduce incorporation efficiency and release control, ultimately compromising bioavailability [19,21].

Moreover, although numerous studies have explored the phytochemical diversity of Brazilian plant species, knowledge about the microorganisms associated with these plants, particularly Amazonian endophytic fungi, remains limited [257]. These microorganisms have demonstrated the ability to synthesize a wide range of bioactive metabolites, including alkaloids, flavonoids, quinones, lignans, and terpenoids [3,258,259].

However, their technological applications remain scarce. Notably, this review identified only one study that incorporated extracts from Amazonian endophytic fungi into biopolymeric films [16], highlighting a significant gap between the discovery of these compounds and their functional application in biomaterials. In this context, it becomes essential to promote interdisciplinary integration among microbiology, biotechnology, process engineering, and materials science to establish optimized routes for the extraction, characterization, and incorporation of microbial metabolites into biopolymeric matrices. According to Singh et al. [259], endophytic fungi function as ‘intracellular chemical factories’, producing a wide variety of natural compounds with efficacy comparable to, and sometimes exceeding, that of synthetic antibiotics—whose clinical effectiveness has been increasingly compromised by microbial resistance. Moreover, fungal-derived metabolites represent a more ecologically sustainable alternative, with reduced environmental impact and lower toxicological risks to human and animal health [260].

Additionally, the mechanisms by which bioactive compounds interact with biopolymeric matrices remain only partially understood, particularly regarding molecular compatibility, release kinetics, and degradation pathways under physiological conditions. Advanced analytical and modeling tools, such as molecular dynamics simulations [261] and multi-omics integration [262], are expected to play a crucial role in elucidating these interactions and optimizing material design. From a technological standpoint, emerging techniques such as 3D printing have enabled the fabrication of structures with controlled architecture and tunable functionality, promoting significant advances in tissue engineering and targeted drug delivery [263,264].

In the regulatory landscape, the lack of standardized international guidelines for biomaterials containing natural bioactive compounds remains one of the main barriers to their clinical translation. To advance these materials from laboratory development to human applications, it is essential to establish rigorous protocols for assessing their safety, therapeutic efficacy, and long-term stability, including standardized in vitro, in vivo, and eventually clinical trials. The absence of specific requirements related to toxicity, sterilization processes, labeling, and traceability poses significant obstacles, particularly for complex and controlled release systems [265,266].

Despite these challenges, the synergy between Amazonian biodiversity and biopolymer science represents a promising strategy for the development of multifunctional and sustainable biomaterials. The adoption of stabilization and controlled release techniques has enabled significant advances in preserving the biological activity of Amazonian compounds while simultaneously promoting the valorization of Brazilian biodiversity and strengthening an environmentally responsible biotechnology.

## 5. Conclusions

This review critically compiled and analyzed the available evidence on Amazonian bioactive compounds and their incorporation into functional biopolymer-based materials. The botanical families addressed in this review were organized according to their botanical classification, chemical composition, and reported biological properties demonstrating clear growth in phytochemical publications in recent years. However, this increase in chemical knowledge has not translated into comparable growth in functional biomaterials.

The subset of studies that do cross into materials development show that the biological function of Amazonian bioactives is stable enough to support the transition from extract to device. Many papers demonstrated that the retention of bioactive compounds depends on the processing route, the chemistry and morphology of the matrix, the loading level, and the physiological environment in which the incorporation occurs. The practical corollary is that the bioactivity of an extract, however thoroughly characterized in solution, does not by itself substantiate a biomaterial claim: activity must be demonstrated after incorporation, alongside the release kinetics that determine whether it reaches its target at all. Therefore, studies must treat characterization in solution as a starting point, since what indicates the suitability for biomedical applications is the activity that persists in the finished system, assessed together with the release kinetics that govern its availability.

Future progress will depend primarily on the systematic engineering of the bioactive–biopolymer interface and elucidation of its interactions through molecular modeling and advanced characterization. Other priority directions include the standardization of raw materials and extracts to overcome batch-to-batch variability, the development of biopolymer matrices incorporating underexplored sources of bioactives, particularly amazonian endophytic fungi, and the development of regulatory and safety protocols for natural-compound-loaded biomaterials. Addressing these points would convert a well-documented chemical inventory of bioactive compounds into a technological platform, simultaneously advancing multifunctional biomaterials and the sustainable valorization of Amazonian biodiversity.

## Figures and Tables

**Table 1:** Applications of biopolymer-based systems incorporating Amazonian bioactives across different administration routes and matrices.

Matrix	Amazonian Source	Biopolymer(s)	Reported Interaction	Evidence Supporting Interaction	Route of administration /Application	Reference
Gels	Buriti oil (*Mauritia flexuosa*)	Chica gun and chitosan	Interactions between buriti oil constituents and the chicha gum/chitosan matrix, associated with changes in thermal behavior and molecular organization	TGA: shifts in initial and final degradation temperatures; FTIR: changes in characteristic absorption bands.	Dermal dressing	[213]
	Açaí oil (*Euterpe oleracea*)	Hyaluronic acid	Hydrogen bonding and molecular association between açaí oil constituents and hyaluronic acid were suggested	FTIR: shift in the amide I carbonyl band of HA after incorporation of açaí oil.	Anti-aging cosmetic	[20]
Scaffolds	Pracaxi oil (*Pentaclethra macroloba*)	Alginate	Hydrogen bonding and crystallinity reduction suggested by FTIR/XRD	Disappearance of diffraction peak at 2*θ* = 31.5° and reduction in OH band intensity	Topical/dermal	[23]
	Buriti oil (*Mauritia flexuosa*)	Polyurethane and Hydroxyapatite	Urethane bond formation during in situ polymerization	FTIR: N–H stretching at 3319 cm^−1^ and C=O stretching at 1693 cm^−1^, supporting polyurethane formation.	Bone tissue	[24]
Particles	Sacha Inchi oil (*Plukenetia volubilis* L.)	Maltodextrin and sodium caseinate	Thermal protection of the bioactive core ensured by the wall material	TGA (corroboration of oil volatilization at specific stages and thermal stability)	Biomaterials	[126]
	Cupuaçu Pulp (*Theobroma grandiflorum*)	Inulin and maltodextrin	Solute stabilization	Increase in glass transition temperature (Tg) observed via response surface methodology	Oral	[18]
	Tucumã oil (*Astrocaryum vulgare* Mart.)	Gum Arabic, Maltodextrin, Dextrin (Capsul^®^) and Modified Starch (SnowFlake^®^)	The interaction of water with each biopolymer depends on the molecular properties of each encapsulation matrix	Analysis of sorption isotherms and water adsorption kinetics	Oral	[94]
	Açaí Pulp oil (*Euterpe oleracea*)	Chitosan and Alginate	Polyelectrolyte complex (PEC) formation via ionic interactions	Shift of the C-N stretching band from 1535 to 1555 cm^−1^ in FTIR	Oral	[38]
	Guaraná (*Paullinia cupana*)	Gum arabic	Electrostatic interaction between polymers	Dissociation of coacervates at pH 7 due to repulsive forces between negatively charged polymers	Oral	[198]
	Buriti oil (*Mauritia flexuosa* L.F)	Gelatin	Hydrophobic interactions between buriti oil and the nonpolar amino acids of gelatin	FTIR (attenuation/shifting of oil bands and appearance of new bands at 1556, 1348, and 1097 cm^−1^)	Oral/pharmaceutical	[214]
	Andiroba (*Carapa guianensis*) and Brazil nut (*Bertholletia excelsa*)	Silk fibroin	Attractive interactions between esters and amino acids suggested by interfacial behavior	Reduction in surface tension and formation of a viscoelastic/gelatinous layer at the oil/water interface	Topical	[26]
	Cupuaçu (*Theobroma grandiflorum*)	Chitosan	Bioadhesive properties suggested by cationic coating	Enhanced marker penetration into injured skin models compared to intact models	Topical/dermal	[19]
	Andiroba (*Carapa guianensis*)	Chitosan	Structural stability reduction suggested by thermal enthalpy	Reduction in enthalpy on DSC curves compared to plain chitosan	Topical/dermal	[215]
Films	Copaíba oil (*Copaifera langsdorffii*)	Chitosan	Crystalline structure modification suggested by XRD	Broadening and disappearance of chitosan diffraction peaks (2*θ* 10–23°)	Topical/dermal	[17]
	Copaíba oil (*Copaifera langsdorffii*)	Pectin	Strengthening of hydrogen bonds between pectin hydroxyl groups and polar compounds (polyphenols) from the oil	FTIR (reduction in -OH stretching) and XRD (peak narrowing and appearance of a new peak at 20.6°)	Topical/dermal	[216]
	Verlot extract (*Arrabidea chica*)	Chitosan and Alginate	Spontaneous PEC formation via electrostatic attraction	Differential association with ionic dyes (Rose Bengal/Methylene Blue) indicating free reactive groups	Topical/dermal	[45]
	Camu-camu powder (*Myrciaria dúbia*)	Gelatin and starch	Interaction between the hydroxyl, ether, and ketone groups of ascorbic acid and the starch and gelatin matrix	Mechanical analysis: reduction in tensile strength and elongation compared with films without the bioactive	Oral	[217]
	Pracaxi oil (*Pentaclethra macroloba*)	Pectin	Hydrogen and covalent interactions between pectin and functional groups/phenolic compounds of pracaxi oil	Reduction in water vapor permeability and increase in permeation activation energy	Topical/dermal	[218]
	*Copaifera officinalis* oleoresin	Chitosan	Molecular interactions between components suggested by Raman and FTIR	Raman and FTIR: significant shifts in bands associated with functional groups of malic acid and glycerin	Mucoadhesive/oral	[135]

## Data Availability

No new data were created or analyzed in this study. Data sharing is not applicable to this article.

## Supplementary Materials

The following supporting information can be downloaded at: https://www.mdpi.com/article/10.3390/ma19163559/s1, Table S1: Main bioactive compounds, biological activities from amazonian plants, and respective nanoencapsulation system developed. Refs. [267,268,269,270,271,272,273,274,275,276,277,278] are included in Supplementary Materials.
