# Supplementary material for: Amazonian Bioactives in Biopolymer-Based Systems for Biomedical Applications: Current Advances and Future Perspectives
Source: Materials (Basel). 2026 Aug 21;19(16):3559. doi: 10.3390/ma19163559 (PMC13514864; doi:10.3390/ma19163559)
Supplement: Supplementary file 1 [file materials-19-03559-s001.zip › materials-4462639-supplementary.pdf]

Table S1. Main bioactive compounds, biological activities from amazonian plants, and respective nanoencapsulation system developed.

Plant source	Plant part used	Compound class	Identified compounds	Biological activity	Biopolimer/ encapsulation	Reference
Mauritia flexuosa (Buriti)	Pulp, pulp oil	Carotenoids	β-carotene, α-carotene, lutein, di-γ-carotene, trans-γ-carotene, di-δ-carotene, di-α-carotene, all-trans-β-carotene, 9-di-β-carotene	Natural pigments in the food industry	Methoxyl pectin	[1,2]
		Tocopherols	α-tocopherol, β-tocopherol, δ-tocopherol, γ-tocopherol	Antiplatelet and antithrombotic activity		[3]
		Phenolic acids	4-hydroxymethyl benzoic acid, caffeic acid, chlorogenic acid, ellagic acid, ferulic acid, gallic acid, mandelic acid, p-anisic acid, p-coumaric acid, p-hydroxybenzoic acid, protocatechuic acid, quinic acid, salicylic acid, sinapic acid, syringic acid, and vanillic acid.	Antioxidant potential, cytotoxicity activity		[4,5]
		Polyphenols (Flavonoids)	(+)-catechin, (−)-epicatechin, 4'-hydroxy-5,7-dimethoxyflavan, 5,4'-dihydroxy-7-methoxy-6-methylflavan, 6,7,4'-trihydroxy-5-methoxyflavan, 7,4'-dihydroxy-5-methoxyflavan, 7,8,4'-trihydroxy-5-methoxyflavan, apigenin, apigenin-7-glucoside, aromadendrin, catechin, chrysin, cyanidin-3-glucoside, cyanidin-3-rutinoside, eugenol, epicatechin, flavanols (or flavan-3-	Antioxidant, antimicrobial, prebiotic, anti-diabetes, and anti-cancer properties		[6–8]

			ols), fustin, galangin, hesperetin, hispidulin, isoorientin, isoquercetin, isoschaftoside, kaempferol, kaempferol-3-O-glucoside, kaempferol-3-O-glucuronide, luteolin, luteolin-7-glucoside, myricetin, naringenin, naringenin-7-glucoside, naringin, nicotiflorine, orientin, pinocembrin, quercetin, quercetin-3-arabinoside, quercetin-3-O-glucuronide, rutin, scoparin, taxifolin, tricine-7-O-rutinoside, vitexin.			
		Anthocyanins	Cyanidin-3-rutinoside, Cyanidin-3-glucoside			[9,10]
		Fatty acids	Myristic acid, Palmitic acid, Palmitoleic acid, Heptadecanoic acid, Stearic acid, Elaidic acid, Oleic acid, Linoleic acid, Linolelalidic acid, Eicosanoic acid, Henicosanoic acid, Behenic acid	Acaricidal, antimicrobial and antioxidant activity	Gelatin, sodium alginate, gum arabic	[11–13]
	Roots	Terpenes	Mauritic acid	Cytotoxicity, antimicrobial activity		[7]
Theobroma grandiflorum (Cupuaçu)	Seeds	Fatty acids	Behenic acid, Ethyl oleate, Galadoleic acid, Lauric acid, Linoleic acid, Linolenic acid, Margaric acid, Margaroleic acid, Methyl (11E)-11-icosenoate, Myristic acid, Oleic acid, Palmitic acid, Palmitoleic acid, Stearic acid	Skin regenerative potential; Cancer immunotherapy enhancement,	Polycaprolactone with chitosan coating	[14–16]

	Pulp	Acids (present in the aroma)	Acetic acid, Propanoic acid, Butanoic acid, Hexanoic acid, Octanoic acid, Nonanoic acid, Isobutyric acid, Isovaleric acid, 2-Hydroxypropanoic acid, 2-Methylbutanoic acid, (*E*)-Hex-3-enoic acid, 2-Ethylhexanoic acid	Antioxidant, anti-inflammatory, and antimicrobial activity		[15,16]
		Alcohols (present in the aroma)	3-Methyl-2-heptanol, 1,3-Butanediol, 2-Phenylethanol, Butanol, Pentanol, Hexanol, Octanol, 2-Propanol, Ethanol, 1-Penten-3-ol, 3-Methylbutanol, 3-Methyl-2-buten-1-ol, (3*Z*)-Hexenol, Benzyl Alcohol, Butane-2,3-diol, Octan-2,3-diol, (*Z*)-Non-6-en-1-ol, 2-Methyl-3-buten-2-ol, 2-Methylpent-1-en-3-ol, 2-Ethylhexan-1-ol, 3-Pentan-2-yloxybutan-2-ol			[16–18]
		Esters (present in the aroma)	Ehtyl 2-methylpentanoate, Butyl hexanoate, Ethyl sorbate, Methyl 3-hidroxybutanoate, Ethyl 3-hydroxybutanoate, Ethyl 3-hydroxyhexanoate, Cinnamyl acetate			[16]
		Ketones (present in the aroma)	3-Hydroxybutan-2-one, 1-Phenyl-2-pentanone, Pentane-2,3-dione, 4-Methypentan-2-one, 3-Methylpentan-2-one, 4-Hydroxy-4-methylpentan-2-one, 6-Methylhept-5-en-2-one, 1-Phenylethanone, 1-Hydroxydecan-3-one			[16]

		Pyrazines (present in the aroma)	Piperazine, 2,3-Dimethylpyrazine, 2-Methylpyrazine, 2,5-Dimethylpyrazine, 2,6-Dimethylpyrazine, 2,3,5-Trimethylpyrazine, 2-Ethyl-3,5-dimethylpyrazine, 2,3,5,6-Tetramethylpyrazine, 1-(3-Methylpyrain-2-yl) ethenone			[16]
		Terpenes (present in the aroma)	Myrcene, (*E*)-b-Ocimene, Terpinolene, (*Z*)-Linalool oxide (furanoid), (*E*)-Linalool oxide (furanoid), Linalool, b-Caryophyllene, a-Terpineol, Linalyl propanoate, Germacrene D, Nerol, Gerianol, Eugenol, g-Decalactone, Cedryl acetate, Limonene, (*Z*)-ocimene, (*Z*)Linalool pyranoxide, (*E*)-Linalool pyranoxide, Geranyl cetone, 2,6-Dimetyl-octa-3,7-dien2,6-diol, 2,6-Dimethyl-oct-7-en-2,6-diol, (*E*)-2,6-Dimethyl-octa-2,7-dien-1,6-diol, (*Z*)-2,6-Dimethyl-octa-2,7-dien-1,6-diol			[16]
		Flavonoids	Apigenin 8-hexoside, Quercitin, Quercitin-3-*O*-b-D-pentoside, Quercitin-3-*O*-b-D-glucoronide, Quercitin-3-*O*-b-D-glucoronide-6¨-methyl ester, Hypolaetin-8-*O*-b-D-flucoronide, Teagrandin II, Hypolaetin-3-methyl ether 8-*O*-b-D-glucoronide, Hypolaetin-3-methyl ether 8-*O*-b-D-glucoronide 3-sulfate, Isoscutellarein-8-*O*-b-D-glucoronide, Isoscutellarein-8-*O*-b-D-	Antioxidant, anti-inflammatory, and immunomodulatory properties, as well as antimicrobial and antihypertensive activities.	Maltodextrin	[19]

			glucoronide 6-methyl ester, Teagrandin I, Apigenin 6-C-glucoside 8-carabinoside, Caffeoyl aspartic acid, -*p*-Coumaryl aspartic acid, Kaempferol, Clovamide, *p*-Coumaryl tyrosine, Cyanidin-3-galactoside, Cyanidin-3-arabinoside			
		Methylxanthines	Caffeine, Theacrine, Theobromine, Theophylline	Psycho-stimulant, anti-inflammatory, anti-cancer, anti-diabetic, and antioxidative		[20]
Myrciaria dubia (Camu-camu)	Pulp, Pulp and peelings without seeds	Anthocyanins	Cyanidin 3-glucoside, cyanidin 3-O-glucoside, delphinidin-3-O-glucoside, and malvidin-3,5-diglucoside.	Antioxidant activities		[21]
		Polyphenols (Non-Flavonoids)	Ellagic acid, trans-Resveratrol			[22]
		Polyphenols (Flavonoids)	apigenin-O-hexoside, (+)-catechin, (−)-epicatechin, epigallocatechin 3-gallate, gallocatechin, quercetin, quercetin-3-O-α-D-arabinofuranoside, quercetin 3-O-hexoside, quercetin 3-O-pentoside, quercetin-3-rutinoside, myricetin, myricetin 3-O-hexoside, myricetin 3-O-pentoside, myricetin-3-O-beta-D-xylopyranoside, myricitrin, (iso)liquiritigenin-O-hexoside, proanthocyanidin A2	Anti-obesity, antidiabetic, anticancer, and anti-inflammatory activities	Maltodextrin, inulin, and oligofructose; gelatin	[23–25]

		Phenolic acids	Caffeic acid, 4-O-caffeoylquinic acid, 5-O-caffeoylquinic acid, Chlorogenic acid, p-Coumaric acid, p-Coumaroyl hexoside, Ferulic acid, Ferulic acid hexoside, Gallic acid, Ellagic acid, Ellagic acid glycoside, Ellagic acid hexoside, Ellagic acid pentoside, Ellagic acid rhamnoside, 2,4-Dihydroxybenzoic acid, 2,5-Dihydroxybenzoic acid, 3,4-Dihydroxybenzoic acid, Rosmarinic acid, Syringic acid, 2-Hydroxycinnamic acid, Valoneic acid dilactone	Antioxidant, anti-inflammatory, antihypertensive, antibacterial, cardioprotective, and neuroprotective activities		[23,25,26]
		Carotenoids	β-carotene			[23]
		Oxygenated organic acids	Ascorbic acid, (S)-4-butoxy-2-hydroxy-4-oxo-butanoic acid and (S)-4-butoxy-3-hydroxy-4-oxo-butanoic acid	Antihyperglycemic activities	Teff starch film; pectin	[26–28]
Euterpe oleracea (açai)	Pulp, oil pulp, seeds	Carotenoids	Lutein, Zeinoxanthin			[29]
		Phenolic acids	Gallic acid, Syringic acid, Chlorogenic acid, Caffeic acid, *p*-Hydroxybenzoic acid, Protocatechuic Acid, p-coumaric, Ellagic acid, Vanillic acid	Anti-inflammatory activities; antimicrobial activity	β-cyclodextrin and HP-β-cyclodextrin	[29–31]
		Anthocyanins	Cyanidin 3-rutinoside, Peonidin 3-rutinoside, Cyanidin 3-glucoside,	Antioxidant properties		[31]

		Phytosterols	β-sitosterol	Hypocholesterolemic properties		[32]
		Fatty acids	Oleic acid, linoleic acid, Palmitoleic acid, Palmitic acid, Stearic acid, monounsaturated fatty acid (MUFA), polyunsaturated fatty acid (PUFA), saturated fatty acid (SFA)	Antioxidant and antimicrobial properties; cytotoxic effects	Hydrophobic surfactant for nanoemulsion; Tween 80, Hyaluronic acid; Chitosan/alginate polyelectrolyte complexes (PECs); desonide and sclerotium gum	[33–36]
Paullinia cupana (guarana)	Seeds	Phenolic acids	Chlorogenic acid, Caffeic acid	Antioxidant potential		[37]
		Flavonols	Quercetin, Kaempferol 7-glucoside, Kaempferol 3-galactoside, Kaempferol 3-rutinoside, Kaempferol 3-rhamnoside, Myricetin 3-glucoside, Quercetin 3-rhamnoside, catechin, epicatechin, procyanidin B2	Hepatoprotective effect	Gelatin and gum Arabic	[37–39]
		Methylxanthines	Theobromine, caffeine			[40]

	Peels	Carotenoids	trans-β-carotene, cis-β-carotene, lutein	Antioxidant capacity	Gum arabic	[40]
Astrocaryum vulgare and *A. aculeatum* (Tucumã)	Pulp	Phenolic acids	Gallic acid, Caffeic acid, Ellagic acid, Chlorogenic acid	Antioxidant potential	Fucoxanthin (FUCO)	[41,42]
		Flavonoids	Catechin, Rutin, Quercetin, Kaempferol	Anti-inflammatory potential		[43]
		Other phenolic compounds	Tannin	Cytoprotective activity		[44]
		Carotenoids	all-trans-β-carotene, all-trans-δ-carotene, all-trans-γ-carotene, cis-γ-carotene, all-trans-α-carotene, all-trans-α-cryptoxanthin, 15-cis-β-carotene, 5,8-epoxy-β-carotene, 13-cis-β-carotene, all-trans-ζ-carotene, all-trans-lutein, all-trans-β-cryptoxanthin, zeinoxanthin, cis-β-zeacarotene 2, cis-β-zeacarotene 1, all-trans-β-zeacarotene, all-trans-neoxanthin, cis-violaxanthin, cis-neoxanthin, all-trans-zeaxanthin, cis-lutein	Antioxidant and provitamin A activities	Spray-dried oil powders encapsulated with gum arabic; Lyophilized pulp powder encapsulated with polymers such as gum arabic, maltodextrin, dextrin and modified starch	[13,45,46]
	Pulp oil	Fatty acids	Palmitic acid, Stearic acid, Oleic acid, cis-vaccenic acid, Linoleic acid, α-linolenic acid	Anti-inflammatory properties, Genotoxicity/antigenot	Nanoemulsions with PPG-5-	[47–49]

				oxicity activity; Antidiabetic activity	Ceteth-20 and sorbitan oleate	
Caryodendron orinocense (cacay)	Oil	Phenolic compounds and polyunsaturated fatty acids	Linoleic acid	Antioxidant, antimicrobial		[50]
Plukenetia volubilis (Pracaxi, *sacha inchi*)	Seeds	Phenolic acids	Protocatechuic acid, p-Coumaric acid, Azaleic acid,	Antioxidant properties		[51]
		Flavonoids	Phloretin-glucoside, Alpinumisoflavone, Luteolin, Myricetin, Apigenin, Isorhamnetin glucoside,			[52]
		Other phenolic compounds	Cinnamic acid, Hydroxycinnamic acid, Hydroxytyrosol, Tyrosol, Bergenin, Pinoresinol, Hydroxy-pinoresinol, Syringaresinol, Oleuropeic acid, Oleuropein aglycone, Ligstroside-aglycone, Oleuropein aglycone- decarboxymethyl dialdehyde	Antioxidative, anti-inflammatory, antiproliferative, analgesic and immunomodulatory activities		[53]
	Oil from seed and leaves	Fatty acids	Palmitic acid, Stearic acid, Oleic acid, Vaccenic acid, Gadoleic acid, Linoleic acid, α-Linolenic acid	Antibacterial, anti-inflammatory, skin tightening and anti-aging effects		[54,55]
		Tocopherols	α-Tocopherol, ß-Tocopherol, γ-Tocopherol, δ-Tocopherol	Antiproliferative activities		[56]

		Phytosterols	Campesterol, Stigmasterol, β-Sitosterol	Antidyslipidaemic and hypoglycemic properties		[57]
Pentaclethra macroloba (Pracaxi)	Seeds	Fatty acids	Caproic acid, Palmitic acid, Stearic acid, Oleic acid, Linoleic acid, Linolenic acid, Behenic acid, Lignoceric acid, Lauric acid, Myristic acid, Arachidic acid, and Erucic acid.	Oxidative stability, healing and insect repellent effects	Dentin cylinders (Ultradent Ultrapack ®)	[58–60]
		Volatile carboxylic acids	Formic acid, Acetic acid, Butyric acid,			[61]
		Tocopherols	α-Tocopherol, β/γ-Tocopherols, δ-Tocopherol, α-Tocotrienol, γ-Tocotrienol			[61]
Virola surinamensis (ucuuba)	Resin	Neolignans	—	leishmanicide activity		[62]
	Leaves	Neolignans and lignans	(-)-5-Demethoxygrandisin B	leishmanicide activity		[63]
Oenocarpus bacaba (Bacaba)	Oil from pulp	Fatty acids	Myristic acid, Palmitic acid, Palmitoleic acid, Stearic acid, Oleic acid, Linoleic acid, Linolenic acid, Arachidic acid, Behenic acid.	Antioxidant potential	System microemulsionated	[64,65]

		Phenolic acids	Gallic acid, Protocatechuic acid, p-Hydroxybenzoic acid, Vanillic acid, Ferulic acid, Sinapic acid	Anti-cancer potential	Acetylated cashew gum polysaccharide and chitosan	[66,67]
		Flavonoids	Quercetin 3-O-rhamnoside, Myricetin 3-rhamnoside, Kaempferol	Anti-inflammatory potential		[68,69]
		Other phenolic compounds	Epicatechin, Procyanidin, Rutin, Catechin	Cardioprotective, and neuroprotective activities		[70]
	Oil	Carotenoids	Lutein, α-carotene, β-carotene, cis lutein, cis α-carotene, cis β-carotene, violaxanthin, antheraxanthin, mutatoxanthin, cis lutein, β-cryptoxanthin			[71,72]
	Oil	Major sterols	β-S + sit, Δ5-A + Δ7-S, Campesterol, Stigmasterol			[73]
*Carapa guianensis (*andiroba)	Seeds	Fatty acids	9-octadecenoic, heneicosanoic, 6,9,12-octadecatrienoic, tricosanoic, 13-docosene, 11,14-eicosadienoic, 7,9-12-octadecadienoic, octadecanoic, docosanoic, 7,9-octadecenoic, decanoic, hexadecanoic, 6-octadecenoic, tetracosanoic, tetradecanoic, dodecanoic, c-10-heptadecenoic, pentadecanoic, heptadecanoic, 9-hexadecenoic, c-10-	Larvicidal activity; Insecticidal and repellent effect; Antiplasmodial activity	Nanoemulsion with surfactant mixture Tween 80:Span 80; incorporated in PCL	[74–77]

			pentadecenoic, tridecanoic, 6,9,12-octadecatrienoic, undecanoic, 11,14,17-eicosatrienoic.		(polycaprolactone)	
		Volatile organic compounds	Hexanal, ethyl butanoate, propane propanoate, 2,4-dimethyl heptane, methyl pentanoate, butan-2-yl propanoate, propan-2-yl butanoate, propyl butanoate, pentanoic acid ethyl ester, propanoic acid butyl ester, butanoic acid 1-methylpropyl ester, pentanoic acid propyl ester, pentanoic acid butyl ester, nonanal, tetradecane, n-hexadecanoic acid.			[78]
	Flowers, seeds	Terpenoids	Gedunin, 6α-Acetoxygedunin, 1,2-Dihydro-3β-Hydroxy-7-Deacetoxy-7-Oxogedunin, Methyl Angolensate, 17β-Hydroxyazadiradione, 11β-Hydroxygedunin, 6α,11β-Diacetoxygedunin, Deoxygedunin, 17-Glycolyldeoxygedunin, Deacetylgedunin, 7-Deacetoxy-7-Oxogedunin, 6α-Hydroxygedunin, Andirobin, 6α-Acetoxyepoxyazadiradione, Andirolide H, Carapanosin C, 17-Epi-17-Hydroxyazadiradione, Carapanolide J, 6-Hydroxymethyl Angolensate, Epoxyazadiradione, Carapanin A, Xyloccensin K, Carapanolide A, B, C, D, E,	Antiproliferative effect; Hepatoprotective effect; cytotoxicity in cell lines; antimalarial activity		[79–81]

			F, G, H, I, K, L, M, N, O, P, Q, R, S, T, U, V, W, X, Carapanosin D, E, F, Carapanin B, C, Guianolide A, B, Carapanosin A, B, Swietephragmin D, G, Guianofruit E, F, G, H, I, A, B, C, D, Guianolactone A, B.			
Bertholletia excelsa (Brazil nut)	Brazil nut cake	Phenolic compounds	Gallic acid, Protocatechuic acid, 2,4 dihydrobenzoic acid, p-hydrobenzoic acid, p-coumaric acid, sinapic acid and catechin.	Antioxidant activity	(OSAstarch) and inulin	[82]
Copaifera L. (copaíba)	Barks	Sesquiterpenes	δ-Elemene, α-Cubebene, α-Ylangene, α-Copaene, β-Elemene, α-Gurjunene, β-Caryophyllene, trans-α-Bergamotene, γ-Elemene, α-Humulene, Alloaromadendrene, α-Curcumene, α-Amorphene, Germacrene D, β-Selinene, α-Muurolene, Bicyclogermacrene, γ-Cadinene, δ-Cadinene,trans-Cadina-1,4-diene, Selina-3,7(11)-diene, Germacrene B	Anti-inflammatory, antimicrobial, antioxidant, and wound healing properties		[83,84]
Acmella oleracea (jambu)	Leaves extract	Alkamides	Spilanthol, N- isobutyl-2,4-undecadiene-8,10-diynamide, N-phenethyl-2-nonene- 6,8-diynamide [6], N-(2-methylbutyl)-2-undecene-8,10-diynamide and N-(2-methylbutyl)- 2,6,8-decatrienamide	Wound healing		[73]
	Leaves extract	Phenolic compounds	Vanilic acid and quercetin	Wound healing		[73]

	Essential oils	Sesquiterpenes	E-caryophyllene	Cytotoxic effect		[85]
	Essential oils	Alkamides	Spilanthol	Muscle relaxing effect		[86]
Arrabidaea chica (crajiru)	Leave extract	—	—	Wound healing		[87]
	Leave extract	Phenolic compounds	Apigenin and luteonin	Antioxidant		[88]
	Leave extract	Phenolic compounds	Kaempferol	Antibacterial		[89]
Pequi (*Caryocar brasiliense*)	Pulp, pulp oil, seed oil	Fatty acids	SFAs (Saturated Fatty Acids): Myristic acid, palmitic acid, margaric acid, stearic acid, arachidic acid, behenic acid, and lignoceric acid<br>MUFAs (Monounsaturated Fatty Acids): Palmitoleic acid, heptadecenoic acid, oleic acid, and gondoic acid.<br>PUFAs (Polyunsaturated Fatty Acids): Linoleic acid and α-linolenic acid	Cardioprotective properties, immune system support, pro- and anti-inflammatory modulation.	Polysorbate 80® (Tween 80), Sodium carboxymethylcellulose (CMC)	[90–94]
		Carotenoids	β-Cryptoxanthin, α-Carotene, Neoxanthin, Violaxanthin, Lutein, Antheraxanthin, Zeaxanthin, β-Carotene	Antioxidant potential, anti-cancer properties	Gelatin, Arabic gum	[95,96]

		Phenolic Acids	Gallic acid, Caffeic acid, p-Coumaric acid, p-Hydroxybenzoic acid	Cardioprotective protective	cashew gum/chitosan;	[95]
		Hydrolyzable Tannins	Gallotannins: Monogalloyl hexoside, Digalloyl hexoside<br>Ellagitannins: HHDP hexoside, HHDP dhexoside, Galloyl-HHDP hexoside	Anti-inflammatory, antioxidant, anti-diabetic, and anti-cancer properties		[95]
		Ellagic Acid and Derivatives	Ellagic acid hexoside, Ellagic acid pentoside, Ellagic acid deoxyhexoside, Ellagic acid, Methyl ellagic acid pentoside, Methyl ellagic acid deoxyhexoside, Dimethyl ellagic acid pentoside	Anti-diabetes, and anti-cancer properties		[95]
		Flavonoids	Coumaroyl quinic acid, Vanillin	Antioxidant and anti-inflammatory properties		[95]
	Peel	Terpenes	Monoterpenes, Sesquiterpenes, Diterpenes, triterpenes, Oxygenated terpenes (terpenoids)			[97]
		Ketones	1-(2,8,8-trimethyl-4,5,6,7-tetrahydrocyclohepta[b]furan-5-yl)ethanone, (E)-3-methyl-4-(1,3,3-trimethyl-7-oxabicyclo[4.1.0]heptan-2-yl)but-3-en-2-one			[97]

		Ester	Pentyl octanoate, Ethyl hexanoate, 2-cyclohexenyl-3-[1-(2-oxopropyl)ethenyl]-2,4,4-trimethyl acetate, 2-ethylhexyl salicylate, Hexyl salicylic acid			[97]
		Aldehyde	(E)-5-(4,5-dimethyl-7a-prop-1-en-2-yl-2,3,3a,5,6,7-hexahydro-1H-inden-4-yl)-3-methylpent-2-enal, Benzeneacetaldehyde			[97]

REFERENCES

1. Moser, P.; Locali-Pereira, A.R.; Nicoletti, V.R. Optimization of Spray Drying Process of Buriti Oil-Loaded Bilayer Emulsions. *Colloids and Surfaces A: Physicochemical and Engineering Aspects* **2024**, *698*, doi:10.1016/j.colsurfa.2024.134516.

2. Cândido, T.L.N.; Silva, M.R.; Agostini-costa, T.S. Bioactive Compounds and Antioxidant Capacity of Buriti (Mauritia Flexuosa L . f .) from the Cerrado and Amazon Biomes. *Food Chemistry* **2015**, *177*, 313–319. 10.1016/j.foodchem.2015.01.041

3. Fuentes, E.; Rodríguez-Pérez, W.; Guzmán, L.; Alarcón, M.; Navarrete, S.; Forero-Doria, O.; Palomo, I. Mauritia Flexuosa Presents in Vitro and in Vivo Antiplatelet and Antithrombotic Activities. *Evidence-based Complementary and Alternative Medicine* **2013**, *2013*, doi:10.1155/2013/653257.

4. Pelosi, M.S.; Leite, I.; Passos, L.; Ferreira, C.; Sabaa-Srur, A.U.O.; Fialgo, E. Physico-Chemical Evaluantion and Antioxidant Capacity Os Burity Extract and Antitumor Activity in Breast Cancer Cell Line MDA_MB-231 2020. 10.34117/bjdv6n4-064

5. Bataglion, G.A.; da Silva, F.M.A.; Eberlin, M.N.; Koolen, H.H.F. Simultaneous Quantification of Phenolic Compounds in Buriti Fruit (Mauritia Flexuosa Lf) by Ultra-High Performance Liquid Chromatography Coupled to Tandem Mass Spectrometry. *Food Research International* **2014**, *66*, 396–400. 10.1016/j.foodres.2014.09.035

6. Abreu-Naranjo, R.; Paredes-Moreta, J.G.; Granda-Albuja, G.; Iturralde, G.; González-Paramás, A.M.; Alvarez-Suarez, J.M. Bioactive Compounds, Phenolic Profile, Antioxidant Capacity and Effectiveness against Lipid Peroxidation of Cell Membranes of Mauritia Flexuosa L. Fruit Extracts from Three Biomes in the Ecuadorian Amazon. *Heliyon* **2020**, *6*, e05211, doi:10.1016/j.heliyon.2020.e05211.

7. Koolen, H.H.F.; da Silva, F.M.A.; Gozzo, F.C.; de Souza, A.Q.L.; de Souza, A.D.L. Antioxidant, Antimicrobial Activities and Characterization of Phenolic Compounds from Buriti (Mauritia Flexuosa L. f.) by UPLC-ESI-MS/MS. *Food Research International* **2013**, *51*, 467–473, doi:10.1016/j.foodres.2013.01.039.

8. Miyasaka, K.; Takeda, S.; Nakamura, S.; Matsuda, H.; Shimoda, H. Estrogenic and Antiandrogenic Activities of Methoxyflavans Isolated from the Fruit of Mauritia Flexuosa (Moriche Palm). *Journal of Food Biochemistry* **2021**, *45*, 2–11, doi:10.1111/jfbc.13583.

9. Barboza, N.L.; dos Anjos Cruz, J.M.; Corrêa, R.F.; Lamarão, C. V; Lima, A.R.; Inada, N.M.; Sanches, E.A.; de Araújo Bezerra, J.; Campelo, P.H. Buriti (Mauritia Flexuosa L. f.): An Amazonian Fruit with Potential Health Benefits. *Food Res. Int.* **2022**, *159*, 111654. 10.1016/j.foodres.2022.111654

10. Koolen, H.H.F.; Soares, E.R.; Da Silva, F.M.A.; De Oliveira, A.A.; De Souza, A.Q.L.; De Medeiros, L.S.; Rodrigues-Filho, E.; Cavalcanti, B.C.; Pessoa, C.O.; Moraes, M.O.; et al.

Mauritic Acid: A New Dammarane Triterpene from the Roots of Mauritia Flexuosa L.f. (Arecaceae). *Natural Product Research* **2013**, *27*, 2118–2125, doi:10.1080/14786419.2013.793685.

11. Castro, G.M.M.A.; Passos, T.S.; Nascimento, S.S. da C.; Medeiros, I.; Araújo, N.K.; Maciel, B.L.L.; Padilha, C.E.; Ramalho, A.M.Z.; Sousa Júnior, F.C.; de Assis, C.F. Gelatin Nanoparticles Enable Water Dispersibility and Potentialize the Antimicrobial Activity of Buriti (Mauritia Flexuosa) Oil. *BMC Biotechnology* **2020**, *20*, 1–13, doi:10.1186/s12896-020-00649-4.

12. Köhn, C.R.; Fagundes, L. da R.; Brandelli, A.; Flôres, S.H.; Rios, A. de O. Microcapsules with Amazonian Buriti Fruit Oil (Mauritia Flexuosa L.) on Maintaining Oxidative Stability of Ground Beef. *Food Hydrocolloids* **2025**, *164*, doi:10.1016/j.foodhyd.2025.111201.

13. Santos, G.S.C.; Morais-Costa, F.; Figueiredo, J.C.G.; Cruz, J.P.; Arrudas, S.R.; Nunes, Y.R.F.; Duarte, E.R.; Vasconcelos, V. de O. Chemical Composition and Acaricidal Activity of Seed Oils of the Palms Mauritia Flexuosa and Mauritiella Armata in Rhipicephalus Microplus (Ixodidae). *Research, Society and Development* **2021**, *10*, e167101321078, doi:10.33448/rsd-v10i13.21078.

14. Barbalho, G.N.; Matos, B.N.; da Silva Brito, G.F.; da Cunha Miranda, T.; Alencar-Silva, T.; Sodré, F.F.; Gelfuso, G.M.; Cunha-Filho, M.; Carvalho, J.L.; da Silva, J.K. do R.; et al. Skin Regenerative Potential of Cupuaçu Seed Extract (Theobroma Grandiflorum), a Native Fruit from the Amazon: Development of a Topical Formulation Based on Chitosan-Coated Nanocapsules. *Pharmaceutics* **2022**, *14*, 207, doi:10.3390/pharmaceutics14010207.

15. Westheim, A.J.F.; Stoffels, L.M.; Dubois, L.J.; Bergenhenegouwen, J. Van; Helvoort, A. Van; Langen, R.C.J. Fatty Acids as a Tool to Boost Cancer Immunotherapy Efficacy. *Frontiers in nutrition* **2022**, *9*, 1–11, doi:10.3389/fnut.2022.868436.

16. da Silva, C.V.A.; Salimo, Z.M.; de Souza, T.A.; Reyes, D.E.; Bassicheto, M.C.; de Medeiros, L.S.; Sartim, M.A.; de Carvalho, J.C.; Goncalves, J.F.C.; Monteiro, W.M. Cupuaçu (Theobroma Grandiflorum): A Multifunctional Amazonian Fruit with Extensive Benefits. *Food Research International* **2024**, *192*, 114729. 10.1016/j.foodres.2024.114729

17. Tauchen, J.; Bortl, L.; Huml, L.; Miksatkova, P.; Doskocil, I.; Marsik, P.; Villegas, P.P.P.; Flores, Y.B.; Van Damme, P.; Lojka, B.; et al. Phenolic Composition, Antioxidant and Anti-Proliferative Activities of Edible and Medicinal Plants from the Peruvian Amazon. *Revista Brasileira de Farmacognosia* **2016**, *26*, 728–737, doi:10.1016/j.bjp.2016.03.016.

18. Ziegleder, G. *Flavour Development in Cocoa and Chocolate*; 2017; ISBN 9781118923597. 10.1002/9781118923597.ch8

19. Cardona, J.E.C.; Carrillo, M.P.; Lares, M.; Gutiérrez, R.H.; Hernández, M.S.; Fernández-Trujillo, J.P. Bioactive Compounds in New Food Products from Amazonic Fruits. *Foods* **2013**, *1*, 1–8. 10.3390/bpqn2013-01193

20. Kim, J.; Kim, J.; Shim, J.; Lee, C.Y.; Lee, K.W.; Kim, J.; Kim, J.; Shim, J.; Lee, C.Y.; Won, K. Cocoa Phytochemicals : Recent Advances in Molecular Mechanisms on Health Cocoa Phytochemicals : Recent Advances in Molecular Mechanisms. *Food Science and Nutrition* **2014**, *8398*, doi:10.1080/10408398.2011.641041.

21. Rodriguez-Cortina, A.; Hernandez-Carrion, M. Amazonian Fruits in Colombia: Exploring Bioactive Compounds and Their Promising Role in Functional Food Innovation. *Journal of Food Composition and Analysis* **2025**, *137*, 106878. 10.1016/j.jfca.2024.106878

22. García-Chacón, J.M.; Rodríguez-Pulido, F.J.; Heredia, F.J.; González-Miret, M.L.; Osorio, C. Characterization and Bioaccessibility Assessment of Bioactive Compounds from Camu-Camu (Myrciaria Dubia) Powders and Their Food Applications. *Food Research International* **2024**, *176*, doi:10.1016/j.foodres.2023.113820.

23. Nowak, D.; Gośliński, M.; Przygoński, K.; Wojtowicz, E. Averrhoa Carambola L., Cyphomandra Betacea, Myrciaria Dubia as a Source of Bioactive Compounds of Antioxidant Properties. *Foods* **2023**, *12*, 753. 10.3390/foods12040753

24. Figueiredo, J. de A.; Teixeira, M.A.; Campelo, P.H.; Lago, A.M.T.; Souza, T.P. de; Yoshida, M.I.; Oliveira, C.R. de; Pereira, A.P.A.; Pastore, G.M.; Sanches, E.A.; et al. Encapsulation of Camu-Camu Extracts Using Prebiotic Biopolymers: Controlled Release of Bioactive Compounds and Effect on Their Physicochemical and Thermal Properties. *Food Research International* **2020**, *137*, doi:10.1016/j.foodres.2020.109563.

25. Fidelis, M.; Santos, J.S.; Escher, G.B.; Vieira do Carmo, M.; Azevedo, L.; Cristina da Silva, M.; Putnik, P.; Granato, D. In Vitro Antioxidant and Antihypertensive Compounds from Camu-Camu (Myrciaria Dubia McVaugh, Myrtaceae) Seed Coat: A Multivariate Structure-Activity Study. *Food and Chemical Toxicology* **2018**, *120*, 479–490, doi:10.1016/j.fct.2018.07.043.

26. Santos, I.L.; Miranda, L.C.F.; da Cruz Rodrigues, A.M.; da Silva, L.H.M.; Amante, E.R. Camu-Camu [Myrciaria Dubia (HBK) McVaugh]: A Review of Properties and Proposals of Products for Integral Valorization of Raw Material. *Food Chemistry* **2022**, *372*, 131290, doi:10.1016/j.foodchem.2021.131290.

27. García-Chacón, J.M.; Tello, E.; Coy-Barrera, E.; Peterson, D.G.; Osorio, C. Mono- n-Butyl Malate-Derived Compounds from Camu-Camu (Myrciaria Dubia) Malic Acid: The Alkyl-Dependent Antihyperglycemic-Related Activity. *ACS Omega* **2022**, *7*, 39335–39346, doi:10.1021/acsomega.2c05551.

28. Ju, A.; Song, K. Bin Development of Teff Starch Films Containing Camu-Camu (Myrciaria Dubia Mc. Vaugh) Extract as an Antioxidant Packaging Material. *Industrial Crops and Products* **2019**, *141*, 111737, doi:10.1016/j.indcrop.2019.111737.

29. Coyago-cruz, E.; Valenzuela, D.; Guachamin, A.; Gabriela, M.; Heredia-moya, J.; Vera, E. Bioactive Compound Profiling and Antioxidant Activity of Phytelephas Tenuicaulis and Other Amazonian Fruits. *foods* **2024**, 1–15. 10.3390/foods13132151

30. Magalhães, T.S.S. de A.; Macedo, P.C. de O.; Pacheco, S.Y.K.; Silva, S.S.; Barbosa, E.G.; Pereira, R.R.; Costa, R.M.R.; Silva Junior, J.O.C.; Silva Ferreira, M.A.; Almeida, J.C.; et al. Development and Evaluation of Antimicrobial and Modulatory Activity of Inclusion Complex of Euterpe Oleracea Mart Oil and β-Cyclodextrin or HP-β-Cyclodextrin. *International Journal of Molecular Sciences* **2020**, *21*, doi:10.3390/ijms21030942.

31. Neri-numa, I.A.; Sancho, R.A.S.; Paula, A.; Pereira, A.; Pastore, G.M. Small Brazilian Wild Fruits : Nutrients , Bioactive Compounds , Health-Promotion Properties and Commercial Interest. *Food Research International* **2018**, *103*, 345–360, doi:10.1016/j.foodres.2017.10.053.

32. Martins, I.C.V.S.; Borges, N.A.; Stenvinkel, P.; Lindholm, B.; Rogez, H.; Pinheiro, M.C.N.; Nascimento, J.L.M.; Mafra, D. The Value of the Brazilian Açai Fruit as a Therapeutic Nutritional Strategy for Chronic Kidney Disease Patients. *International Urology and Nephrology* **2018**, *50*, 2207–2220, doi:10.1007/s11255-018-1912-z.

33. Borges, K.R.A.; Wolff, L.A.S.; da Silva, M.A.C.N.; de Carvalho Silva, A.K.; Campos, C.D.L.; Souza, F.S.; Teles, A.M.; Vale, A.Á.M.; Pascoa, H.; Lima, E.M.; et al. Açaí (Euterpe Oleracea Mart.) Seed Oil and Its Nanoemulsion: Chemical Characterisation, Toxicity Evaluation, Antioxidant and Anticancer Activities. *Current Issues in Molecular Biology* **2024**, *46*, 3763–3793, doi:10.3390/cimb46050235.

34. Monge-Fuentes, V.; Muehlmann, L.A.; Longo, J.P.F.; Silva, J.R.; Fascineli, M.L.; Azevedo, R.B.; de Souza, P.; Faria, F.; Degterev, I.A.; Rodriguez, A.; et al. Photodynamic Therapy Mediated by Acai Oil (Euterpe Oleracea Martius) in Nanoemulsion: A Potential Treatment for Melanoma. *Journal of Photochemistry and Photobiology B: Biology* **2017**, *166*, 301–310, doi:10.1016/j.jphotobiol.2016.12.002.

35. Sanches, S.C. da C.; Ré, M.I.; Silva-Júnior, J.O.C.; Ribeiro-Costa, R.M. Organogel of Acai Oil in Cosmetics: Microstructure, Stability, Rheology and Mechanical Properties. *Gels* **2023**, *9*, 150, doi:10.3390/gels9020150.

36. Teixeira-Costa, B.E.; Silva Pereira, B.C.; Lopes, G.K.; Tristão Andrade, C. Encapsulation and Antioxidant Activity of Assai Pulp Oil (Euterpe Oleracea) in Chitosan/Alginate Polyelectrolyte Complexes. *Food Hydrocolloids* **2020**, *109*, 106097, doi:10.1016/j.foodhyd.2020.106097.

37. Dabulici, C.M.; S rbu, I.; Vamanu, E. The Bioactive Potential of Functional Products and Bioavailability of Phenolic Compounds. *Foods* **2020**, *9*, doi:10.3390/foods9070953.

38. Kober, H.; Tatsch, E.; Torbitz, V.D.; Cargnin, L.P.; Sangoi, M.B.; Bochi, G.V.; Da Silva, A.R.H.; Barbisan, F.; Ribeiro, E.E.; Da Cruz, I.B.M.; et al. Genoprotective and Hepatoprotective Effects of Guarana (Paullinia Cupana Mart. Var. Sorbilis) on CCl4-Induced Liver Damage in Rats. *Drug and Chemical Toxicology* **2016**, *39*, 48–52, doi:10.3109/01480545.2015.1020546.

39. Silva, A.P.G. da; Sganzerla, W.G.; Jacomino, A.P.; Silva, E.P. da; Xiao, J.; Simal-Gandara, J. Chemical Composition, Bioactive Compounds, and Perspectives for the Industrial Formulation of Health Products from Uvaia (Eugenia Pyriformis Cambess – Myrtaceae): A Comprehensive Review. *Journal of Food Composition and Analysis* **2022**, *109*, doi:10.1016/j.jfca.2022.104500.

40. Pinho, L.S.; da Silva, M.P.; Thomazini, M.; Cooperstone, J.L.; Campanella, O.H.; da Costa Rodrigues, C.E.; Favaro-Trindade, C.S. Guaraná (Paullinia Cupana) by-Product as a Source of Bioactive Compounds and as a Natural Antioxidant for Food Applications. *Journal of Food Processing and Preservation* **2021**, *45*, 1–13, doi:10.1111/jfpp.15854.

41. Cordenonsi, L.M.; Santer, A.; Sponchiado, R.M.; Wingert, N.R.; Raffin, R.P.; Schapoval, E.E.S. Amazonia Products in Novel Lipid Nanoparticles for Fucoxanthin Encapsulation. *AAPS PharmSciTech* **2020**, *21*, 1–10, doi:10.1208/s12249-019-1601-y.

42. Nascimento, J.V.; Silva, K.A.; Giuliangeli, V.C.; Mendes, A.L.D.; Piai, L.P.; Michels, R.N.; Dal Bosco, T.C.; Ströher, G.R.; Shirai, M.A. Starch-PVA Based Films with Clitoria Ternatea Flower Extract: Characterization, Phenolic Compounds Release and Compostability. *International Journal of Biological Macromolecules* **2024**, *255*, 128232, doi:10.1016/j.ijbiomac.2023.128232.

43. Cabral, F.L.; Bernardes, V.M.; Passos, D.F.; Oliveira, J.S. De; Doleski, P.H.; Silveira, K.L.; Horvarth, M.C.; Bremm, J.M.; Barbisan, F.; Azzolin, V.F.; et al. Astrocaryum Aculeatum Fruit Improves in Fl Ammation and Redox Balance in Phytohemagglutinin-Stimulated Macrophages. *Journal of Ethnopharmacology* **2020**, *247*, 112274, doi:10.1016/j.jep.2019.112274.

44. Sagrillo, M.R.; Garcia, L.F.M.; De Souza Filho, O.C.; Duarte, M.M.M.F.; Ribeiro, E.E.; Cadoná, F.C.; Da Cruz, I.B.M. Tucumã Fruit Extracts (Astrocaryum Aculeatum Meyer) Decrease Cytotoxic Effects of Hydrogen Peroxide on Human Lymphocytes. *Food Chemistry* **2015**, *173*, 741–748, doi:10.1016/j.foodchem.2014.10.067.

45. Amorim, I.S.; Amorim, D.S.; Godoy, H.T.; Mariutti, L.R.B.; Chisté, R.C.; da Silva Pena, R.; Bogusz Junior, S.; Chim, J.F. Amazonian Palm Tree Fruits: From Nutritional Value to Diversity of New Food Products. *Heliyon* **2024**, *10*, 1–17, doi:10.1016/j.heliyon.2024.e24054.

46. Silva, R.S.; de L. Santos, C.; Mar, J.M.; Kluczkovski, A.M.; de A. Figueiredo, J.; Borges, S. V.; Bakry, A.M.; Sanches, E.A.; Campelo, P.H. Physicochemical Properties of Tucumã (Astrocaryum Aculeatum) Powders with Different Carbohydrate Biopolymers. *Lwt* **2018**, *94*, 79–86, doi:10.1016/j.lwt.2018.04.047.

47. Almeida, F.; Corrêa, M.; Zaera, A.M.; Garrigues, T.; Isaac, V. Influence of Different Surfactants on Development of Nanoemulsion Containing Fixed Oil from an Amazon Palm Species. *Colloids and Surfaces A: Physicochemical and Engineering Aspects* **2022**, *643*, doi:10.1016/j.colsurfa.2022.128721.

48. Baldissera, M.D.; Souza, C.F.; Doleski, P.H.; Grando, T.H.; Sagrillo, M.R.; da Silva, A.S.; Leal, D.B.R.; Monteiro, S.G. Treatment with Tucumã Oil (Astrocaryum Vulgare) for Diabetic Mice Prevents Changes in Seric Enzymes of the Purinergic System: Improvement of Immune System. *Biomedicine and Pharmacotherapy* **2017**, *94*, 374–379, doi:10.1016/j.biopha.2017.07.113.

49. Carneiro, A.B.A.; Pinto, E.J.S.; Ribeiro, I.F.; Magalhães, M.R.G.; Neto, M.D.A.B.M. Effect of Astrocaryum Aculeatum (Tucumã) on Doxorubicin Toxicity: In Vivo Experimental Model. *ACTA Paulista de Enfermagem* **2017**, *30*, 233–239, doi:10.1590/1982-0194201700036.

50. Azevedo, W.M. De; Ferreira, L.; Oliveira, R. De; Alves, M.; Maria, A.; Magalh, T. De; Suzanne, K.; Arau, K. De; Assis, C.F. De; Damasceno, S.C. Physicochemical Characterization , Fatty Acid Profile , Antioxidant Activity and Antibacterial Potential of Cacay Oil , Coconut Oil and Cacay Butter. *PLoS ONE* **2020**, 1–11, doi:10.1371/journal.pone.0232224.

51. Chirinos, R.; Necochea, O.; Pedreschi, R.; Campos, D. Sacha Inchi (Plukenetia Volubilis L.) Shell: An Alternative Source of Phenolic Compounds and Antioxidants. *International Journal of Food Science and Technology* **2016**, *51*, 986–993, doi:10.1111/ijfs.13049.

52. Goyal, A.; Tanwar, B.; Kumar Sihag, M.; Sharma, V. Sacha Inchi (Plukenetia Volubilis L.): An Emerging Source of Nutrients, Omega-3 Fatty Acid and Phytochemicals. *Food Chemistry* **2022**, *373*, 131459, doi:10.1016/j.foodchem.2021.131459.

53. Štěrbová, L.; Hlásná Čepková, P.; Viehmannová, I.; Huansi, D.C. Effect of Thermal Processing on Phenolic Content, Tocopherols and Antioxidant Activity of Sacha Inchi Kernels. *Journal of Food Processing and Preservation* **2017**, *41*, e12848. 10.1111/jfpp.12848

54. Gonzalez-Aspajo, G.; Belkhelfa, H.; Haddioui-Hbabi, L.; Bourdy, G.; Deharo, E. Sacha Inchi Oil (Plukenetia Volubilis L.), Effect on Adherence of Staphylococus Aureus to Human Skin Explant and Keratinocytes in Vitro. *Journal of Ethnopharmacology* **2015**, *171*, 330–334, doi:10.1016/j.jep.2015.06.009.

55. Schiessel, D.L.; Yamazaki, R.K.; Kryczyk, M.; Coelho, I.; Yamaguchi, A.A.; Pequito, D.C.T.; Brito, G.A.P.; Borghetti, G.; Fernandes, L.C. α-Linolenic Fatty Acid Supplementation Decreases Tumor Growth and Cachexia Parameters in Walker 256 Tumor-Bearing Rats. *Nutrition and Cancer* **2015**, *67*, 839–846, doi:10.1080/01635581.2015.1043021.

56. Rodzi, N.A.R.M.; Lee, L.K. Corrigendum to “Sacha Inchi (Plukenetia Volubilis L.): Recent Insight on Phytochemistry, Pharmacology, Organoleptic, Safety and Toxicity Perspectives” [Heliyon 8, (9), (September 2022), (2022), E10572] (Heliyon (2022) 8(9), (S2405844022018606), (10.1016/j. *Heliyon* **2022**, *8*, doi:10.1016/j.heliyon.2022.e11436.

57. Ambulay, J.P.; Rojas, P.A.; Timoteo, O.S.; Barreto, T. V.; Colarossi, A. Effect of the Emulsion of Sacha Inchi (Plukenetia Huayabambana) Oil on Oxidative Stress and

Inflammation in Rats Induced to Obesity. *Journal of Functional Foods* **2020**, *64*, 0–8, doi:10.1016/j.jff.2019.103631.

58. Fasciotti, M.; Monteiro, T.V.C.; Rocha, W.F.C.; Morais, L.R.B.; Sussulini, A.; Eberlin, M.N.; Cunha, V.S. Comprehensive Triacylglycerol Characterization of Oils and Butters of 15 Amazonian Oleaginous Species by ESI-HRMS/MS and Comparison with Common Edible Oils and Fats. *European Journal of Lipid Science and Technology* **2020**, *122*, doi:10.1002/ejlt.202000019.

59. Gioster-Ramos, M.L.; Pedrinha, V.F.; Barros, M.C.; Bezerra, R.M.; Andrade, F.B.; Kuga, M.C.; Vaz, L.G. Antimicrobial Effect of Pentaclethra Macroloba Plant Extract against Enterococcus Faecalis. *Brazilian Journal of Biology* **2023**, *83*, 1–7, doi:10.1590/1519-6984.272095.

60. Teixeira, G.L.; Maciel, L.G.; Mazzutti, S.; Gonçalves, C.B.; Ferreira, S.R.S.; Block, J.M. Composition, Thermal Behavior and Antioxidant Activity of Pracaxi (Pentaclethra Macroloba) Seed Oil Obtained by Supercritical CO2. *Biocatalysis and Agricultural Biotechnology* **2020**, *24*, doi:10.1016/j.bcab.2020.101521.

61. Souza, I. De; Ramos, C.; Lemos, M.; Carneiro, B.; Silva, S.; Lauane, P.; Fernandes, T.; Anselmo, G. Effects of Andiroba Oil (Carapa Guianensis Aublet) on the Immune System in Inflammation and Wound Healing : A Scoping Review Aim ˆ. *Journal of ethnopharmacology* **2024**, *327*. https://doi.org/10.1016/j.jep.2024.118004

62. Veiga, A.; Albuquerque, K.; Corrêa, M.E.; Brigido, H.; Silva e Silva, J.; Campos, M.; Silveira, F.; Santos, L.; Dolabela, M. Leishmania Amazonensis and Leishmania Chagasi: In Vitro Leishmanicide Activity of Virola Surinamensis (Rol.) Warb. *Experimental Parasitology* **2017**, *175*, 68–73, doi:10.1016/j.exppara.2017.02.005.

63. Paes, S.S.; Silva-Silva, J.V.; Portal Gomes, P.W.; Silva, L.O. da; Costa, A.P.L. da; Lopes Júnior, M.L.; Hardoim, D. de J.; Moragas-Tellis, C.J.; Taniwaki, N.N.; Bertho, A.L.; et al. (-)-5-Demethoxygrandisin B a New Lignan from Virola Surinamensis (Rol.) Warb. Leaves: Evaluation of the Leishmanicidal Activity by In Vitro and In Silico Approaches. *Pharmaceutics* **2023**, *15*, doi:10.3390/pharmaceutics15092292.

64. Maria, V.; Cunha, B.; Paiva, M.; Henrique, S.; Sousa, B. De; Bezerra, N.; Gama, E.; Menezes, O.; Janaina, N.; Ariane, D.; et al. The Journal of Supercritical Fluids Bacaba-de-Leque (Oenocarpus Distichus Mart .) Oil Extraction Using Supercritical CO 2 and Bioactive Compounds Determination in the Residual Pulp. *The Journal of Supercritical Fluids* **2019**, *144*, 81–90, doi:10.1016/j.supflu.2018.10.010.

65. Torres, M. de P.R.; Esprendor, R.V. de F.; Bonaldo, S.M.; Ribeiro, E.B.; Valladão, D.M. de S. Development, Characterization and Stability of Microemulsionated Formulations of Bacaba, Oenocarpus Bacaba Oil. *Acta Amazonica* **2019**, *49*, 246–255, doi:10.1590/1809-4392201802702.

66. Abadio Finco, F.D.B.; Kloss, L.; Graeve, L. Bacaba (Oenocarpus Bacaba) Phenolic Extract Induces Apoptosis in the MCF-7 Breast Cancer Cell Line via the Mitochondria-Dependent Pathway. *NFS Journal* **2016**, *5*, 5–15, doi:10.1016/j.nfs.2016.11.001.

67. ALVES, V.M.; AMORIM, K.A.; ASQUIERI, E.R.; da Silva ARAÚJO, E.; de SOUZA, A.R.M.; MELO, A.A.M.; de Almeida MURADIM, L.B.; DAMIANI, C. Chemical, Nutritional, Anti-Nutritional, and Technological Profile of Bacaba Fractions (Oenocarpus Bacaba Mart.): Promising Fruit from the Brazilian Legal Amazon. *Food Science and Technology* **2025**, *45*. 10.5327/fst.00396

68. Corrêa, B.S.; Costa, M.S.; Cabrera-Pasca, G.A.; Sena, C.; Holanda Pinto, R.H.; Silva, A.P.S.; Carvalho Junior, R.N.; Ishida, L.; Ramon, J.G.A.; Freitas, R.S.; et al. High-Saturation Magnetization in Small Nanoparticles of Fe3O4 Coated with Natural Oils. *Journal of Nanoparticle Research* **2020**, *22*, doi:10.1007/s11051-020-4761-5.

69. Pinto, R.H.H.; Sena, C.; Santos, O. V; Da Costa, W.A.; Rodrigues, A.M. da C.; Junior, R.N.C. Extraction of Bacaba (Oenocarpus Bacaba) Oil with Supercritical CO 2: Global Yield Isotherms, Fatty Acid Composition, Functional Quality, Oxidative Stability, Spectroscopic Profile and Antioxidant Activity. *Grasas y aceites* **2018**, *69*, e246–e246. 10.3989/gya.0883171

70. Nascimento, R.A. do; Andrade, E.L.; Santana, E.B.; Ribeiro, N. da F.P.; Costa, C.M.L.; Faria, L.J.G. de Bacaba Powder Produced in Spouted Bed: An Alternative Source of Bioactive Compounds and Energy Food Product. *Brazilian Journal of Food Technology* **2019**, *22*, 1–15, doi:10.1590/1981-6723.22918.

71. Santos, M.F.G.; Alves, R.E.; Roca, M. Carotenoid Composition in Oils Obtained from Palm Fruits from the Brazilian Amazon. *Grasas y Aceites* **2015**, *66*, doi:10.3989/gya.1062142.

72. Soares, S.; Tom, A.B.; Garcia-rojas, E.E. Microencapsulation of Curcumin by Complex Coacervation of Lactoferrin and Carboxymethyl Tara Gum for Incorporation into Edible Films. *Food Hydrocolloids for Health* **2024**, *5*, doi:10.1016/j.fhfh.2024.100178.

73. Fajardo, J.B.; Vianna, M.H.; Polo, A.B.; Cordeiro Comitre, M.R.; de Oliveira, D.A.; Ferreira, T.G.; de Oliveira Lemos, A.S.; Souza, T. de F.; Campos, L.M.; de Lima Paula, P.; et al. Insights into the Bioactive Potential of the Amazonian Species Acmella Oleracea Leaves Extract: A Focus on Wound Healing Applications. *Journal of Ethnopharmacology* **2025**, *337*, doi:10.1016/j.jep.2024.118866.

74. Melo, K.M.; Oliveira, L.F.S.; da Rocha, R.M.; Ferreira, M.A.P.; Fascineli, M.L.; Milhomem-Paixão, S.S.R.; Grisolia, C.K.; Santos, A.S.; Salgado, H.L.C.; Muehlmann, L.A.; et al. Andiroba Oil and Nanoemulsion (Carapa Guianensis Aublet) Reduce Lesion Severity Caused by the Antineoplastic Agent Doxorubicin in Mice. *Biomedicine and Pharmacotherapy* **2021**, *138*, doi:10.1016/j.biopha.2021.111505.

75. Morikawa, T.; Nagatomo, A.; Kitazawa, K.; Muraoka, O.; Kikuchi, T.; Yamada, T.; Tanaka, R.; Ninomiya, K. Collagen Synthesis-Promoting Effects of Andiroba Oil and Its Limonoid Constituents in Normal Human Dermal Fibroblasts. *Journal of Oleo Science* **2018**, *67*, 1271–1277, doi:10.5650/jos.ess18143.

76. Sarquis, I.R.; Sarquis, R.S.F.R.; Marinho, V.H.S.; Neves, F.B.; Araújo, I.F.; Damasceno, L.F.; Ferreira, R.M.A.; Souto, R.N.P.; Carvalho, J.C.T.; Ferreira, I.M. Carapa Guianensis Aubl. (Meliaceae) Oil Associated with Silk Fibroin, as Alternative to Traditional Surfactants, and Active against Larvae of the Vector Aedes Aegypti. *Industrial Crops and Products* **2020**, *157*, doi:10.1016/j.indcrop.2020.112931.

77. Silva, D.F.; Lima, K.T.; Bastos, G.N.T.; Oliveira, J.A.R.; Do Nascimento, L.A.S.; Costa, C.E.F.; Filho, G.N.R.; Concha, V.O.C.; Passos, M.F. PCL/Andiroba Oil (Carapa Guianensis Aubl.) Hybrid Film for Wound Healing Applications. *Polymers* **2021**, *13*, 1–15, doi:10.3390/polym13101591.

78. Leal, A. de B.; Hackbart, H.C. dos S.; Macueia, F.B.E.D.; Rombaldi, C.V. Andiroba Oil (Carapa Guianensis): Descriptive Analysis and Applicability. *Industrial Crops and Products* **2023**, *202*, doi:10.1016/j.indcrop.2023.117004.

79. Ninomiya, K.; Miyazawa, S.; Ozeki, K.; Matsuo, N.; Muraoka, O.; Kikuchi, T.; Yamada, T.; Tanaka, R.; Morikawa, T. Hepatoprotective Limonoids from Andiroba (Carapa Guianensis). *International Journal of Molecular Sciences* **2016**, *17*, doi:10.3390/ijms17040591.

80. Pereira da Silva, V.; de Carvalho Brito, L.; Mesquita Marques, A.; da Cunha Camillo, F.; Raquel Figueiredo, M. Bioactive Limonoids from Carapa Guianensis Seeds Oil and the Sustainable Use of Its By-Products. *Current Research in Toxicology* 2023, *4*. 10.1016/j.crtox.2023.100104

81. Tanaka, Y.; Sakamoto, A.; Inoue, T.; Yamada, T.; Kikuchi, T.; Kajimoto, T.; Muraoka, O.; Sato, A.; Wataya, Y.; Kim, H.S.; et al. Andirolides H-P from the Flower of Andiroba (Carapa Guianensis, Meliaceae). *Tetrahedron* **2012**, *68*, 3669–3677, doi:10.1016/j.tet.2011.12.076.

82. Gomes, S.; Torres, A.G. Optimized Extraction of Polyphenolic Antioxidant Compounds from Brazil Nut (Bertholletia Excelsa) Cake and Evaluation of the Polyphenol Profile by HPLC. *Journal Science Food Agric* **2015**, doi:10.1002/jsfa.7448.

83. Barbosa, P.C.S.; Wiedemann, L.S.M.; Medeiros, R.S.; Sampaio, P.D.T.B.; Vieira, G.; Veiga-Junior, V.F. Da Phytochemical Fingerprints of Copaiba Oils (Copaifera Multijuga Hayne) Determined by Multivariate Analysis. *Chemistry and Biodiversity* **2013**, *10*, 1350–1360, doi:10.1002/cbdv.201200356.

84. Caputo, L.S.; Campos, M.I.C.; Dias, H.J.; Crotti, A.E.M.; Fajardo, J.B.; Vanelli, C.P.; Presto, Á.C.D.; Alves, M.S.; Aarestrup, F.M.; Paula, A.C.C.; et al. Copaiba Oil Suppresses

Inflammation in Asthmatic Lungs of BALB/c Mice Induced with Ovalbumin. *International Immunopharmacology* **2020**, *80*, 106177, doi:10.1016/j.intimp.2019.106177.

85. Jerônimo, L.B.; de Araújo, J.A.C.; da Silva, J.K.R.; Mourão, R.H. V.; Setzer, W.N.; Figueiredo, P.L.B. Seasonality's Effects on the Chemical Composition and Antiradical Capacity of the Floral Essential Oil of Acmella Oleracea (L.) R.K. Jansen Cultivated in the Brazilian Amazon. *Horticulturae* **2024**, *10*, doi:10.3390/horticulturae10090925.

86. Sampaio, I.M.G.; Teixeira, B.J.B.; Bittencourt, R.F.P. de M.; Pinheiro, M.S.S.; de Oliveira, E.S.; Rogez, H.L.G.; da Silva Júnior, M.L. Biomass and Spilanthol Content of Hydroponic Jambu as a Function of Nitrogen Supply. *Revista Brasileira de Engenharia Agricola e Ambiental* **2024**, *28*, doi:10.1590/1807-1929/agriambi.v28n12e277584.

87. Jorge, M.P.; Madjarof, C.; Ruiz, A.L.T.G.; Fernandes, A.T.; Rodrigues, R.A.F.; de Oliveira Sousa, I.M.; Foglio, M.A.; de Carvalho, J.E. Evaluation of Wound Healing Properties of Arrabidaea Chica Verlot Extract. *Journal of Ethnopharmacology* **2008**, *118*, 361–366, doi:10.1016/j.jep.2008.04.024.

88. Amaral, R.R.; Santos, A.A.D.; Saravia, A.; Botas, G.; Cruz, R.A.S.; Fernandes, C.P.; Rocha, L.; Boylan, F. Biological Activities of Arrabidaea Chica (Bonpl.) B. Verl. Leaves. *Latin American Journal of Pharmacy* **2012**, *31*, 451–455. http://www.latamjpharm.org/resumenes/31/3/LAJOP_31_3_1_15.pdf

89. Mafioleti, L.; da Silva Junior, I.F.; Colodel, E.M.; Flach, A.; de Oliveira Martins, D.T. Evaluation of the Toxicity and Antimicrobial Activity of Hydroethanolic Extract of Arrabidaea Chica (Humb. & Bonpl.) B. Verl. *Journal of ethnopharmacology* **2013**, *150*, 576–582. 10.1016/j.jep.2013.09.008

90. Brito, V.P.; Souza Ribeiro, M.M.; Viganó, J.; Moraes, M.A.; Veggi, P.C. Silk Fibroin Hydrogels Incorporated with the Antioxidant Extract of Stryphnodendron Adstringens Bark. *Polymers* **2022**, *14*, 4806, doi:10.3390/polym14224806.

91. Cicero, N.; Albergamo, A.; Salvo, A.; Daniel, G.; Bartolomeo, G.; Mangano, V.; Rotondo, A.; Di, V.; Di, G.; Dugo, G. Chemical Characterization of a Variety of Cold-Pressed Gourmet Oils Available on the Brazilian Market. *Food Research International journal* **2018**, *109*, 517–525. 10.3390/foods14010046

92. Olteanu, G.; Mititelu, M.; Lupuliasa, D.; Neacs, S.M.; Busnatu, S.; Mihai, A.; Popovici, V.; Nicoleta, M.; Mihai, S.; Ionit, C. Polyunsaturated Fatty Acids and Human Health : A Key to Modern Nutritional Balance in Association with Polyphenolic Compounds from Food Sources. *foods* **2025**.

93. da Silva, D.A.; da Cruz Rodrigues, A.M.; dos Santos, A.O.; Salvador-Reyes, R.; da Silva, L.H.M. Physicochemical and Technological Properties of Pracaxi Oil, Cupuassu Fat and Palm Stearin Blends Enzymatically Interesterified for Food Applications. *LWT* **2023**, *184*, 114961. https://doi.org/10.1016/j.lwt.2023.114961

94. Pereira, T.F.; Borchardt, H.; Wanderley, W.F.; Vasconcelos, U.; Leite, I.F. Pequi Pulp (Caryocar Brasiliense) Oil-Loaded Emulsions as Cosmetic Products for Topical Use. **2025**. https://doi.org/10.3390/polym17020226

95. Nascimento-Silva, N.R.R. Do; Naves, M.M.V. Potential of Whole Pequi (Caryocar Spp.) Fruit—Pulp, Almond, Oil, and Shell—as a Medicinal Food. *Journal of Medicinal Food* **2019**, *22*, 952–962. https://doi.org/10.1089/jmf.2018.0149

96. Justi, P.N.; Sanjinez-Argandoña, E.J.; Macedo, M.L.R. Microencapsulation of Pequi Pulp Oil by Complex Coacervation. *Revista Brasileira de Fruticultura* **2018**, *40*, e-874. 10.1590/0100-29452018874

97. Santos, R.S. dos; Vecchi, C.F.; Rosseto, H.C.; Bassi da Silva, J.; Dano, M.E.L.; de Castro-Hoshino, L.V.; Baesso, M.L.; Bruschi, M.L. Emulgels Containing Carbopol 934P and Different Vegetable Oils for Topical Propolis Delivery: Bioadhesion, Drug Release Profile, and Ex Vivo Skin Permeation Studies. *AAPS PharmSciTech* **2020**, *21*, 1–10, doi:10.1208/s12249-020-01748-3.
